# Dynamic allostery drives autocrine and paracrine TGF-β signaling

**DOI:** 10.1016/j.cell.2024.08.036

**Published:** 2024-09-16

**Authors:** Mingliang Jin, Robert I. Seed, Guoqing Cai, Tiffany Shing, Li Wang, Saburo Ito, Anthony Cormier, Stephanie A. Wankowicz, Jillian M. Jespersen, Jody L. Baron, Nicholas D. Carey, Melody G. Campbell, Zanlin Yu, Phu K. Tang, Pilar Cossio, Weihua Wen, Jianlong Lou, James Marks, Stephen L. Nishimura, Yifan Cheng

**Affiliations:** 1 Department of Biochemistry and Biophysics, University of California San Francisco (UCSF), San Francisco, CA, USA; 2 Department of Pathology, UCSF, San Francisco, CA, USA; 3 Department of Bioengineering and Therapeutic Sciences, UCSF, San Francisco, CA, USA; 4 Department of Medicine and UCSF Liver Center, UCSF, San Francisco, CA, USA; 5 Center for Computational Mathematics, Flatiron Institute, 10010 NY, USA; 6 Center for Computational Biology, Flatiron Institute, 10010 NY, USA; 7 Department of Anesthesia and Perioperative Care, UCSF, San Francisco, CA, USA; 8 Howard Hughes Medical Institute, UCSF, San Francisco, CA, USA Current address:; 9 Division of Respiratory Diseases, Department of Internal Medicine, Jikei University School of Medicine, Tokyo, Japan; 10 Quantoom France, 4 rue Pierre Fontaine, 91000 Evry-Courcouronnes, France; 11 Basic Sciences Division, Fred Hutchinson Cancer Center, Seattle, WA, USA; 12 Porter Neuroscience Research Center, NINDS, National Institutes of Health, Bethesda, MD 20892, USA; 13 Senior authors; 14 Lead Contact

## Abstract

TGF-β, essential for development and immunity, is expressed as a latent complex (L-TGF-β) non-covalently associated with its prodomain and presented on immune cell surfaces by covalent association with GARP. Binding to integrin αvβ8 activates L-TGF-β1/GARP. The dogma is that mature TGF-β must physically dissociate from L-TGF-β1 for signaling to occur. Our previous studies discovered that αvβ8-mediated TGF-β autocrine signaling can occur without TGF-β1 release from its latent form. Here, we show mice engineered to express TGF-β1 that cannot release from L-TGF-β1 survive without early lethal tissue inflammation of TGF-β1 deficiency. Combining cryogenic electron microscopy with cell-based assays we reveal a dynamic allosteric mechanism of autocrine TGF-β1 signaling without release where αvβ8 binding redistributes intrinsic flexibility of L-TGF-β1 to expose TGF-β1 to its receptors. Dynamic allostery explains the TGF-β3 latency/activation mechanism and why TGF-β3 functions distinctly from TGF-β1, suggesting it broadly applies to other flexible cell surface receptor/ligand systems.

## In Brief

Genetically engineered mice survive with only autocrine but no paracrine TGF-β1 signaling. Structural and functional studies reveal a mechanism for TGF-β1 autocrine signaling driven by conformational entropy redistribution from αvβ8 binding to latent TGF-β1/GARP complex. Integrin-mediated entropy redistribution also underlies TGF-β3 activation suggesting a general mechanism of cell-cell communication.

## Graphical Abstract

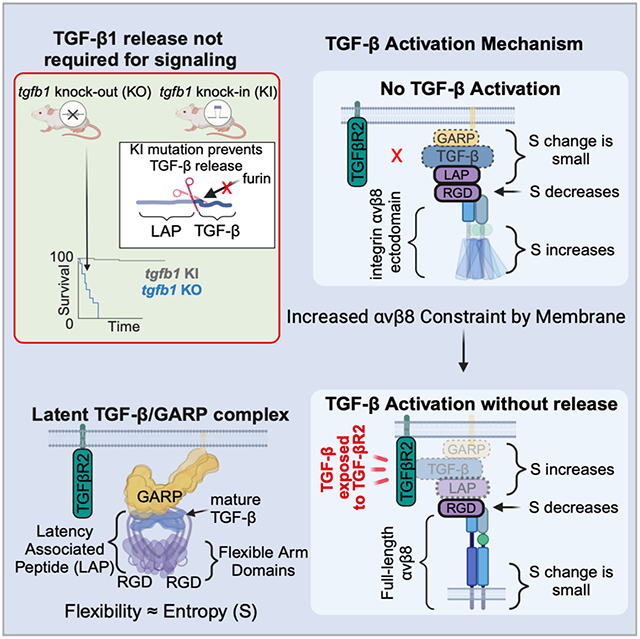

## Introduction:

Transforming growth factor-β (TGF-β1) is a multifunctional cytokine with key roles in development, immunity, cancer, and fibrosis^1–3^. TGF-β has three distinct gene products (TGF-β1, -β1, and -β3) all expressed in inactive (latent) forms (L-TGF-β) and “activation” is essential for function^4^. Most therapeutic TGF-β targeting strategies have not focused on specific latency and/or activation mechanisms, but rather on global inhibition of TGF-β signaling and have significant toxicities^3^. Improved understanding of latency and activation may facilitate better therapeutic approaches targeting TGF-β.

Latency of mature TGF-β is determined by non-covalent association with its N-terminal prodomain cleaved by furin during biosynthesis^5,6^. The prodomain encircles mature TGF-β homodimer in a ring-shaped disulfide linked homodimer “straitjacket”, (latency-associated peptide, LAP), forming L-TGF-β^5^. LAPs serve four essential functions: 1) conferring latency through shielding mature TGF-β from its receptors via the lasso domains of the straitjacket^5^; 2) sequestering L-TGF-β to the matrix or cell-surface through binding to TGF-β milieu molecules such as GARP, which stabilizes and covalently links L-TGF-β to cell surfaces^7–9^; 3) facilitating proper folding and efficient secretion^10^; 4) binding to essential activating proteins, in particular, integrins^1,11^.

Active mature TGF-βs are disulfide-linked homodimers highly conserved in TGF-β receptor (TGF-βR) binding domains, particularly mature TGF-β1 and -β3, which bind with similar affinities to TGF-β receptors (TGF-βR1/TGF-βR2)^12,13^. Despite this conservation, mice deficient in TGF-β1 or TGF-β3 have distinct phenotypes, potentially due to individual mechanisms of latency and/or activation as predicted by overall low homology between LAPs of TGF-β1 and -β3 (Figure S1)^14–17^. Interestingly, both TGF-β1 and -β3 LAPs contain the integrin binding motif RGDLXXL/I and bind to two integrins, αvβ6 and αvβ8, which together account for the majority of TGF-β1, and some of TGF-β3 function, in vivo^11,18,19^. Integrin binding culminates in TGF-β activation leading to autocrine^20^ or paracrine^21^ TGF-β signaling by mechanisms that remain speculative^18,19,22,23^.

Structural and sequence differences between integrins αvβ6 and αvβ8 suggest distinct mechanisms of TGF-β activation likely contributing to context-specific functions of TGF-β^11,18,19,22,24–26^. In the case of αvβ6, global conformational changes transduce force from the actin-cytoskeleton to L-TGF-β disrupting LAP allowing release of mature TGF-β for paracrine signaling^23^. This mechanism requires the highly conserved β6-subunit cytoplasmic domain, which binds the actin cytoskeleton^19^. However, released mature TGF-β1 from αvβ8-mediated activation is difficult to detect, indicating inefficient paracrine TGF-β1 signaling^22,27^. Accordingly, αvβ8 does not undergo global conformational changes^28,29^, αvβ8-mediated TGF-β1 activation does not require actin-cytoskeleton force generation, since β8 cytoplasmic domain is not required for activation, and does not bind to actin^18^. Our previous work revealed that αvβ8 binding induces flexibility in the L-TGF-β1 straitjacket leading us to hypothesize that mature TGF-β1 can be activated without release from the latent complex, which we confirmed in cell-based assays^22^. Thus, we hypothesized that flexibility generated by binding L-TGF-β1 to αvβ8 is sufficient to expose mature TGF-β1 to TGF-βRs for autocrine signaling without being released^22^. Yet, it remains unclear how without mechanical force, αvβ8 binding mechanistically induces L-TGF-β flexibility when L-TGF-β is stabilized by binding to GARP, and whether such a mechanism is physiologically relevant, as it is widely assumed that release and paracrine signaling of TGF-β is required for its function^1^.

In this study, we first validate autocrine signaling without TGF-β1 release is physiologically relevant. We engineer knock-in mice globally expressing only *tgfb1* with a mutated furin cleavage site that cannot release TGF-β1. TGF-β signaling in these mice remains intact as they survive, breed, and are spared from lethal early tissue inflammation of TGF-β1 deficiency, proving that mature TGF-β bound to its latent complex can be activated, bind to its receptors and signal^30^. We next pursue the mechanism allowing TGF-β1 to bind to TGF-βRs without release. We describe a dynamic allosteric model whereby, upon binding to αvβ8, reduction of local conformational entropy around the L-TGF-β RGD binding region increases conformational entropy around distal regions of L-TGF-β/GARP, exposing mature TGF-β to TGF-βRs without release. In support of this model, we determine structures of L-TGF-β3/GARP showing the degree of basal conformational entropy of L-TGF-β1 and -β3 not only determines the basal level of integrin independent TGF-β activation, but also entropy available to drive integrin-dependent TGF-β activation. Higher levels of integrin-mediated entropic change in L-TGF-β3 than -β1 result in paracrine release of mature TGF-β3 but not -β1, indicating isoform-specific mechanisms of autocrine and paracrine TGF-β signaling. Furthermore, the direction of entropy redistribution can be manipulated by stabilizing different flexible domains of αvβ8/L-TGF-β/GARP. Overall, our structural and cell-based approaches reveal a protein dynamic-based allosteric mechanism of redistributing conformational entropy at large distances across protein complexes that is actin cytoskeletal force-independent and determines autocrine and paracrine TGF-β functions. Together, these results advance mechanistic understanding of latency and activation of TGF-β family members providing a roadmap for structural understanding of protein dynamic-mediated signal propagation through flexible cell surface proteins.

## Results:

### Autocrine TGF-β1 signaling without release prevents lethal tissue inflammation caused by global TGF-β1 deficiency

**Figure 1.**
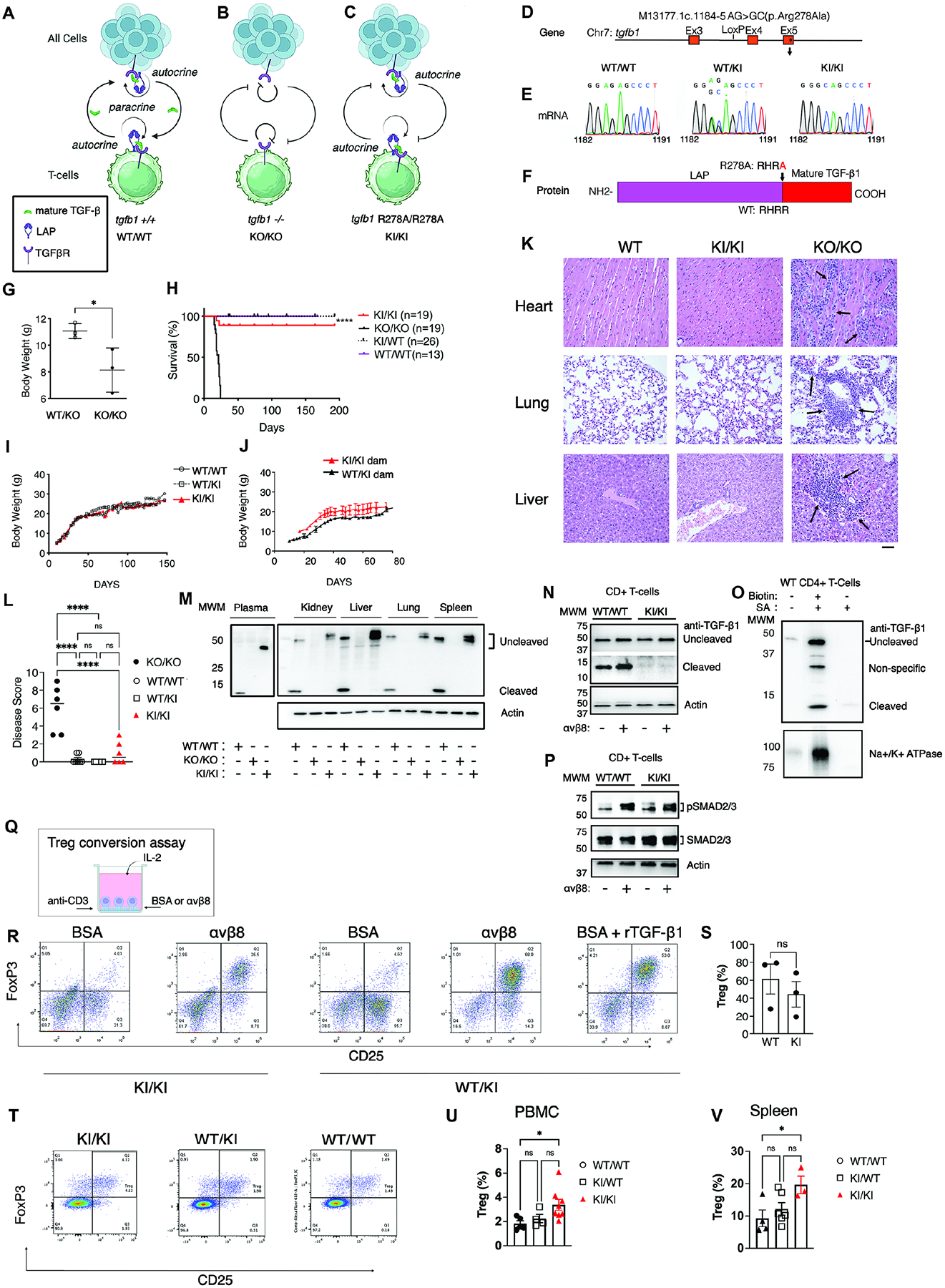
Autocrine TGF-β1 signaling without release prevents lethal tissue inflammation caused by global TGF-β1 deficiency (**A-C**) Cartoons of autocrine and paracrine TGF-β1 signaling in wild type (WT/WT, **A**), *tgfb1*^−/−^ (KO/KO, **B**) and knock-in (KI/KI, **C**) mice with furin cleavage site mutation (*tgfb*^*R278A*/*R278A*^). Inset shows symbol key. KI/KI mice generated as in Figure S2A. **(D)** Schematic showing location of recombined targeted *tgfb1* locus on chromosome 7 (GenBank: M13177.1). **(E)** Sequencing chromatograms of WT/WT, WT/KI and KI/KI mice with corresponding sequence shown above. **(F)** Schematic of one TGF-β1 protein monomer and position of LAP, furin cleavage site, mutated R278A sequence, and mature TGF-β1 peptide. **(G)** Body weights of KO/KO mice at post-natal day 18 compared to littermate WT/KO controls. Shown is the mean and standard error (s.e.m.). *p<0.05 Student’s t-test. **(H)** KI/KI, WT/KI and WT/WT mice survive to adulthood compared to KO/KO mice which die by 24 days of multiorgan inflammation. **** p<0.0001 of all groups compared to KO/KO by Mantel-Cox. **(I)** Body weights over time of KI/KI, WT/KI and WT/WT mice. **(J)** Body weights over time of KI/KI mice born from KI/KI or WT/KI dams. **(K)** Organ histology (heart, upper; lung, middle; liver, lower panels) showing hematoxylin and eosin staining of representative fields of tissue sections from WT/WT (left), KI/KI (middle), KO/KO (right) mice (n ≥ 5 mice). Bar = 30 μm. **(L)** Scatter plots of disease scores from mice KO/KO (n=6), WT/WT (n=6), WT/KI (n=5), KI/KI (n=6) mice, represented by filled circles, open circles, open squares, or red filled triangles respectively. Shown is mean, s.e.m.. ANOVA followed by Tukey’s multiple comparison test, ****p<0.0001. **(M)** Anti-mature TGF-β1 immunoblot using equal amounts of plasma, or organ lysates (kidney, liver, lung, or spleen), under reducing conditions, from WT/WT, KO/KO or KI/KI mice. + or − below indicate respective genotypes. Positions of molecular weight markers (MWM) on left. Expected positions of ~50kDa and 12.5kDa uncleaved and cleaved TGF-β1 bands on right. Below, immunoblot using anti-actin as a protein loading control. **(N)** CD4+ T-cells from WT/WT or KI/KI mice cultured on BSA or immobilized αvβ8tr, as indicated below image. Mature TGF-β1 detected by immunoblotting as in **M**. Upper: shorter exposure; middle: longer exposure to show mature TGF-β1 in WT CD4+ T-cells. Lower panel represents same membrane stripped and reprobed with anti-actin. Shown is a representative experiment (n=3). **(O)** Non-cleaved TGF-β1 is present on the surface of WT CD4+ T-cells. Activated WT mouse CD4+ T-cell surface biotinylation, capture on streptavidin agarose (SA), and detection as above. Below, membrane stripped and reprobed with anti-Na+/K+ ATPase, as a cell membrane marker. Shown is a representative experiment of 3 with similar results. **(P)** Same lysates from activated CD4+T-cells from **N**, demonstrating (upper panel) increased αvβ8-mediated TGF-β signaling detected by anti-phospho-SMAD2/3 (pSMAD2/3). Note pSMAD2 migrates slightly slower than pSMAD3; total SMAD2/3 (middle); actin (lower). Shown is a representative experiment (n=3). **(Q)** Cartoon showing generation of induced-Treg (iTreg). **(R)** CD4+ T-cells in activating conditions from KI/KI compared to controls, WT/WT (or WT/KI) mice plated on BSA (± recombinant TGF-β1 as a positive control) or immobilized αvβ8 ectodomain, as indicated. CD4+ T-cells stained with anti-FoxP3 and anti-CD25 and representative quadrant scatterplots shown. FoxP3+, CD25+ Treg in the upper right quadrant. **(S)** Graphs showing Treg as percentage of activated CD4+ T-cells. Shown is s.e.m. ns = not significant. **(T)** Representative scatterplots of peripheral blood mononuclear cells (PBMC) from KI/KI, and age and littermate matched controls (WT/WT or WT/KI), as indicated, and stained as in **R**. (**U, V**) Treg enumerated from adult mouse PBMC (n=9 WT/WT and WT/KI; n=8 KI/KI) (**U**); or spleen at post-natal day ~18–21, n=6 WT/WT and WT/KI; n=3 KI/KI)(**V**) show no decrease in Treg compared with reduced Treg percentages in KO/KO mice (Figure S2). Shown is s.e.m. ns = not significant. *p<0.05 See also Figure S2.

TGF-β signals through both autocrine and paracrine mechanisms (Figure 1A). TGF-β1 deficient mice lack both autocrine and paracrine TGF-β1 signaling from all cells and die early of widespread tissue inflammation (Figure 1B)^30^. This is attributed to TGF-β signaling in T-cells, since this same phenotype is observed when TGF-β receptors are deleted from T-cells^31,32^. Whether T-cells receive primarily autocrine or paracrine TGF-β1 signals is not well understood. Our recent structural and cell-based studies demonstrated that TGF-β1 release was not required for autocrine TGF-β1 signaling^22^. We test the physiological significance of this finding by creating mice with a mutation in the canonical furin recognition sequence (^275^RXRR^278^↓) in TGF-β1 (*tgfb1*^*R278A/R278A*^) that cannot cleave mature TGF-β1 from LAP and are thus only capable of autocrine but not paracrine signaling (Figure 1C–F, S2). We hypothesize that if non-released mature TGF-β1 productively binds to TGF-βRs and induces autocrine signaling, mutant mice are rescued from universal early lethal tissue inflammation of TGF-β1 deficiency^30^. *Tgfb1*^−/−^ mice begin to show signs of wasting by 10–14 days and die within 24 days (Figure 1G, H). *Tgfb1*^*R278A/R278A*^ mice are phenotypically indistinguishable from *tgfb1*^*R278A/WT*^ and WT littermates up to 240 days (at the time of this manuscript submission) showing similar post-natal survival, and weight gain (Figure 1H–J). Genetic approaches to determine the *in vivo* role of TGF-β1 are confounded by contributions of maternal endocrine TGF-β1 supplied transplacentally during development and after birth through breast milk. Maternal derived TGF-β1 from *tgfb1*^−/−^ dams partially compensates for fetal TGF-β1 deficiency allowing *tgfb1*^−/−^ mice to be born alive and survive until weaning before succumbing to autoimmunity^30,33^. When maternal TGF-β1 is absent, *tgfb1*^−/−^ mice die immediately after birth^34^. We demonstrate endocrine release of cleaved mature TGF-β from maternal sources is dispensable since *tgfb1*^*R278A/R278A*^ mice can be derived from homozygous *tgfb1*^*R278A/R278A*^ dams and show similar post-natal survival, gain weight, and are phenotypically indistinguishable from *tgfb1*^*R278A/R278A*^ mice born from *tgfb1*^*R278A/WT*^ dams (Figure 1J, Movie S1). The organs of *tgfb1*^*R278A/R278A*^ mice are histologically indistinguishable from WT and *tgfb1*^*R278A/WT*^ mice, in contrast with *tgfb1*^−/−^ mice, which display massive immune infiltration of heart, liver and lungs (Figure 1K, L, Table S1). Therefore, autocrine TGF-β1 signaling without release rescues the early lethal tissue inflammation of TGF-β1 deficiency, and endocrine or paracrine release of TGF-β1 is not involved or required for this rescue.

To validate these findings, we performed several controls. We verified *tgfb1*^*R278A/R278A*^ mice show no evidence of mature TGF-β1 cleavage (Figure 1M, N) and found non-cleaved TGF-β1 prominently expressed in WT lysates from organs and CD4+ T-cells (Figure 1M, N) and easily detected on surfaces of WT CD4+ T-cells suggesting autocrine TGF-β1 signaling without release can also occur in WT mice (Figure 1O). We confirmed non-cleaved TGF-β1 induces sufficient TGF-β signaling (Figure 1P) to generate immunosuppressive regulatory T-cells (Tregs) *in vitro* (Figure 1Q–S), and in vivo (Figure 1T–V).

Taken together, our findings support the physiological relevance of autocrine TGF-β1 signaling without release of mature TGF-β.

### Structures of the L-TGF-β1/GARP and αvβ8/L-TGF-β1/GARP complexes

**Figure 2.**
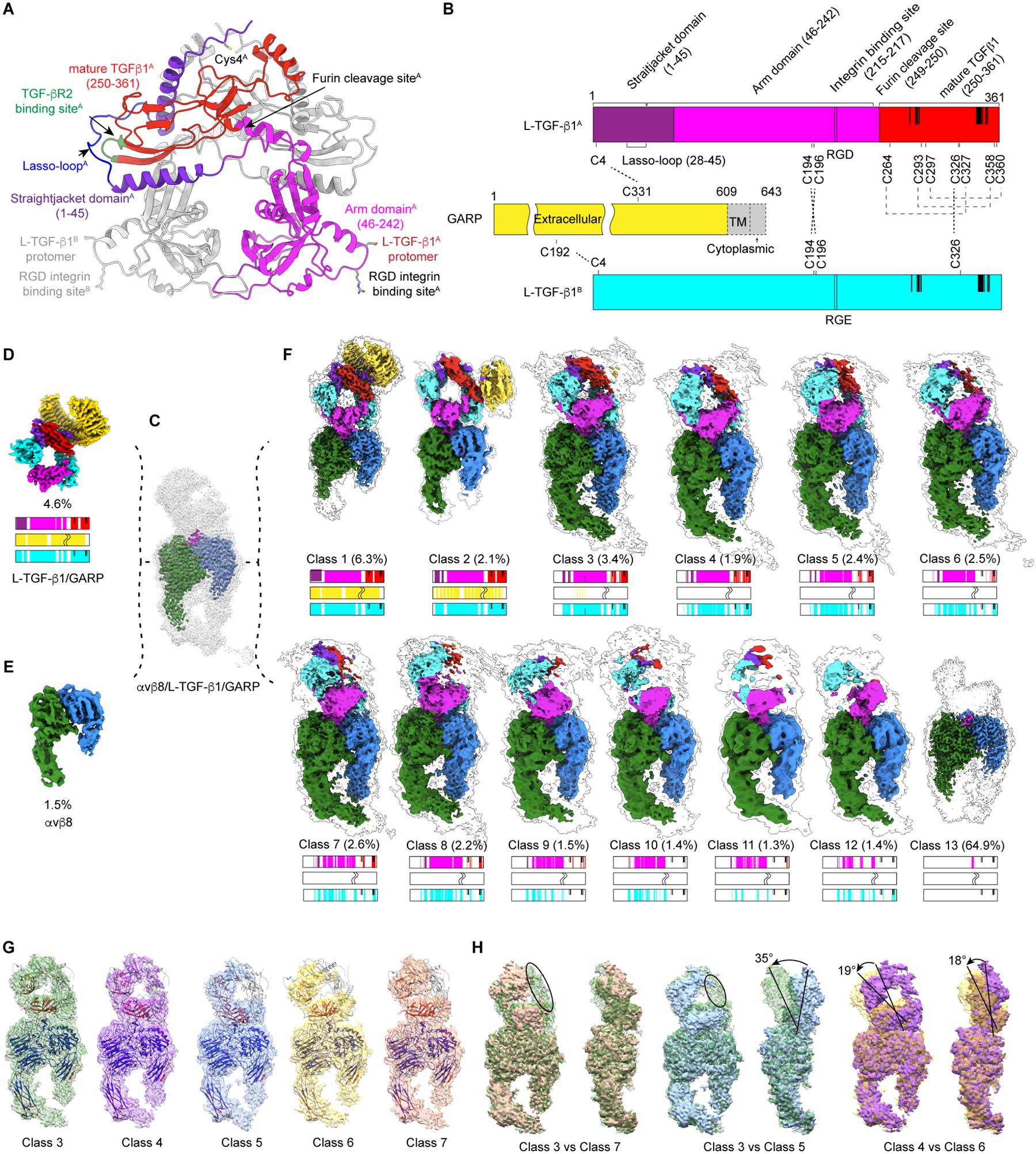
Structures and conformational flexibility of L-TGF-β1/GARP alone and bound with αvβ8 **(A)** Atomic model of L-TGF-β1 dimer with domain in protomer A colored and marked. **(B)** Schematic of L-TGF-β1/GARP constructs. Numbering starts after signal peptide. Two protomers and their respective RGD motif or RGE mutations, resolved cysteines, TGF-βR2 binding sites, and GARP transmembrane truncation indicated. **(C)** Density maps of αvβ8/L-TGF-β1/GARP complex displayed with two thresholds, low in transparent grey, high in solid color. Color code: integrin αv-green, β8-blue. **(D)** and (**E**) Density maps of L-TGF-β1/GARP (**D**) and αvβ8 (**E**) determined from classification of (**C**). **(F)** Density maps of 13 sub-classes of αvβ8/L-TGF-β1/GARP, arranged from best to least resolved L-TGF-β1/GARP. Maps of class 3 −12 displayed at same two thresholds. Color scheme of L-TGF-β1 follows schematic in (**A**). Percentage below each map represents fraction of particles of the class. Bars are colored as in (**A**), with the unresolved regions shown in white. **(G)** Five selected classes from (**F**) illustrating L-TGF-β1 motion relative to αvβ8. Ribbon diagram of αvβ8 and L-TGF-β1 docked within maps, arbitrarily colored. **(H)** Comparison between sub-classes shown in (**G**) illustrate increased flexibility (class 3 vs. 7), degree and direction of motion (class 3 vs. 5, and 4 vs. 6). Structures aligned to each other using αv β-propeller domain showing L-TGF-β/GARP rocking on top of αvβ8. See also Figure S3.

To address mechanisms allowing TGF-β1 to bind to TGF-βRs without release, we use single particle cryogenic electron microscopy (cryo-EM) to study complexes of L-TGF-β1/GARP (Figure 2A, B) and αvβ8/L-TGF-β1/GARP in solution. By mixing L-TGF-β1/GARP with recombinant αvβ8 ectodomain (1:1 molar ratio), we obtained anticipated proportions of 1:1 and 2:1 αvβ8:L-TGF-β1/GARP complexes as revealed by mass photometry (Figure S3A) and single particle cryo-EM (Figures 2C–G, S3). Using a cell-based TGF-β1 activation assay, we demonstrated one αvβ8 is sufficient to activate TGF-β1 from L-TGF-β1/GARP for signaling (Figure S3B). Thus, while we determined structures for both 2:1 (Figure S3E) and 1:1 αvβ8:L-TGF-β1/GARP complexes, we focused on the 1:1 complex obtaining a structure at 2.5Å resolution (Figure 2C, S3E). By further intensive classification, we isolate a small percentage of unbound L-TGF-β1/GARP (4.6% particles, at 3.4Å, Figure 2D) and αvβ8 (1.5% of particles, at 4.5Å, Figure 2E), with remaining particles of the trimeric complex in many different conformations (93.9% of total particles, resolution 2.5Å-8.3Å, Figure 2F–H). In addition, we determined a 3.0Å resolution structure of L-TGF-β1/GARP (Figure S3H) from the purified L-TGF-β1/GARP sample.

**Figure 3.**
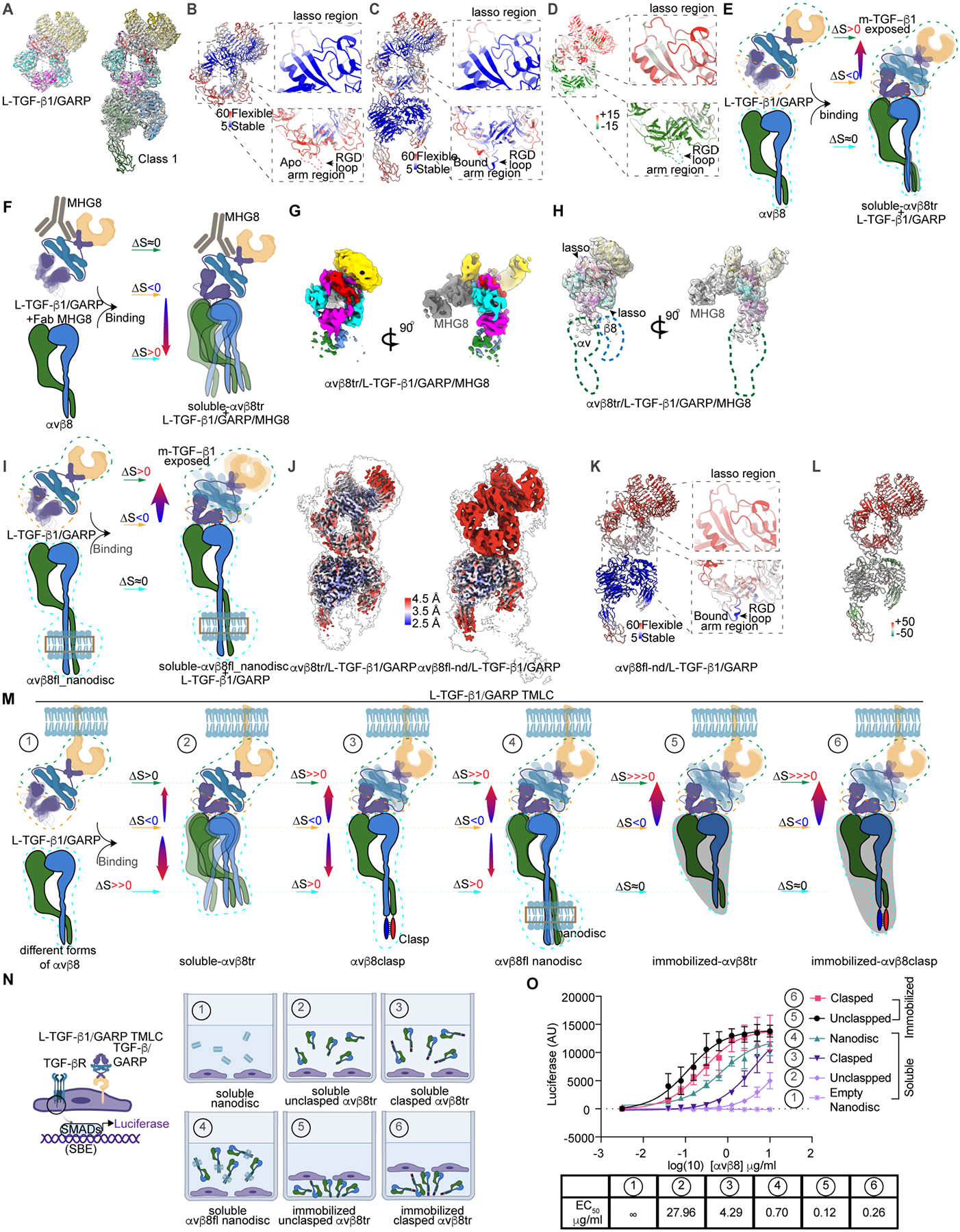
Spatial entropy redistribution upon L-TGF-β1/GARP binding to αvβ8 **(A)** Density maps of L-TGF-β1/GARP (left) independently determined and αvβ8/L-TGF-β1/GARP (right, Class 1 from Figure 2F) docked with refined atomic model. **(B)** and **(C)** Ribbon diagram of L-TGF-β1/GARP (**B**), and αvβ8/L-TGF-β1/GARP (**C**). Residues colored by normalized B-factors with range indicated by scale bar. Two enlarged views show lasso loop (upper) and RGD containing arm domain (lower). Dashed loop, lower panel (**B**), indicates unresolved RGD loop. **(D)** Ribbon diagram of L-TGF-β1 colored with normalized B-factor changes before and after binding to αvβ8, decreased (green), increased (red). Arm domain binding to integrin becomes stabilized (lower dashed box with enlarged view), while straitjacket (containing the lasso, upper dashed box with enlarged view) and GARP become more flexible. **(E)** Model illustrating conformational entropy redistribution upon complex formation. Different regions are circled with colored dashed lines. ΔS<0, reduction of local entropy; ΔS>0, increase of local entropy; ΔS≈0, no change in local entropy. Blurring of ribbon diagrams indicates domain flexibility observed in structures. **(F)** Predicted spatial entropy redistribution upon L-TGF-β1/GARP/MHG8 binding to αvβ8. Labeling nomenclature same as (**E**). **(G)** and (**H**) Two different views of αvβ8/L-TGF-β1/GARP/MHG8 map (**G**) and docked with atomic model (**H**). L-TGF-β1/GARP/MHG8 is almost entirely resolved. Only a very small part of the integrin head domain is resolved. (**I**) Predicted spatial entropy redistribution upon L-TGF-β1/GARP binding to αvβ8fl-nd. (**J**) Comparison of local resolutions between reconstructions of αvβ8tr and αvβ8fl-nd in complex with L-TGF-β1/GARP. Local resolutions color coded by same scale. Both densities are displayed with two density thresholds. **(K)** Ribbon diagram of αvβ8fl-nd/L-TGF-β1/GARP. Residues colored by normalized B-factors with range indicated by scale bar. Enlarged views within dashed boxes show lasso loop (upper) and RGD containing arm domain (lower). **(L)** Ribbon diagram of αvβ8/L-TGF-β1/GARP colored with changes of normalized B-factor between αvβ8fl-nd and αvβ8tr in complex with L-TGF-β1/GARP, decreased (green) and increased (red). **(M)** Predicted entropy redistribution upon binding of soluble αvβ8tr, αvβ8fl-nd and immobilized αvβ8tr to cell membrane bound L-TGF-β1/GARP. Labeling nomenclature same as (**E**). Anchoring in cell membrane presumably increases stability of L-TGF-β1/GARP (panel 1). Upon binding αvβ8tr, conformational entropy is redistributed from L-TGF-β1 arm towards integrin αvβ8 (panel 2). Stabilization of αvβ8tr via clasped and nanodisc reconstitution of αvβ8fl redistributes conformational entropy largely towards straitjacket domain (panel 3 and 4). Immobilizing αvβ8tr drives entropy redistribution almost entirely towards the straitjacket, inducing sufficient flexibility for efficient TGF-β activation (panel 5 and 6). **(N)** Schematics showing design of TMLC reporter cell assays of TGF-β activation without αvβ8 (1), soluble αvβ8tr without (2) or with C-terminal constraint (3), soluble αvβ8fl-nd (panel 4), or globally stabilized immobilized αvβ8tr (5), or clasped αvβ8tr (6). **(O)** Activation of TGF-β by soluble and immobilized αvβ8tr (clasped and unclasped), αvβ8fl-nd and immobilized αvβ8tr using the assay configuration and numbering as in (**N**). See also Figure S4.

Overall, the cryo-EM structure of L-TGF-β1/GARP determined alone is largely consistent with its crystal structure (PDB: 6GFF)^5^, except that we connect the straitjacket domain to the contralateral arm domain on the opposite side of L-TGF-β1. In most of the structure, the resolution is sufficient to resolve sidechains for reliable atomic model building (Figures 3A, left). The local resolution of the density map and the temperature-factor (B-factor) of individual residues obtained from real space refinement of the model are consistent (Figures 3B, S3E). We further subject L-TGF-β1/GARP to 1 μs all-atom molecular dynamics simulations revealing clear correlation between the per-residue root-mean-square-fluctuation (RMSF) and B-factor (Figure S4A). RMSF measures local structural flexibility and dynamics^35^. Thus, local resolution or B-factor provides quantitative measurement of relative flexibility of specific regions, which clearly show half the straitjacket domain (including the lasso) is more flexible (Figure 3B, enlarged view in the upper panel) than the analogous portion of the other straitjacket within the same L-TGF-β1 (Figure 3B enlarged view in lower panel). Our results suggest, in solution, extensive interaction stabilizes the portion of the straitjacket domain in contact with GARP (Figure 3B enlarged view in the upper panel), and exposure of mature TGF-β1 may require disruption of this extensive interaction.

### Structural dynamics and induced flexibility of αvβ8/L-TGF-β1/GARP

Based on structures of L -TGF-β1/GARP alone and in complex with αvβ8, we hypothesized that integrin binding to L-TGF-β1/GARP further induces flexibility of GARP, L-TGF-β1 or both, leading to destabilization of the L-TGF-β1/GARP interface and lasso loops. In all snapshots that reflect motion and flexibility of GARP/L-TGF-β1 relative to αvβ8 (Figures 2F–2H and S3C–E), the domain close to the RGD binding loop is always resolved but density of the remaining part of L-TGF-β1/GARP is progressively weaker (Figures 2F). Despite only being resolved in two snapshots (Figures 2F **class 1 and 2**), GARP is present in all L-TGF-β1 particles, since a disulfide bond forms between GARP and each L-TGF-β1 monomer^9^. Extensive focused classification and alignment, together with 3D variability analysis (3DVA), reveal rocking motions of L-TGF-β1/GARP relative to αvβ8 (Figures 2G and 2H, S3F and S3G, and movie S2). Beyond rocking, we observe progressive loss of density from GARP to the straitjacket domain as range of motions increase (Figures 2G and 2H, S4B). Visualizing both rocking and progressive changes of local resolution in GARP and the straitjacket rules out the possibility that loss of density is caused by particle misalignment rather than increased flexibility. Thus, we conclude that the disappearance of GARP in the reconstructions is caused by the increased flexibility of the straitjacket domain.

In one snapshot (**class 1 in** Figures 2F and 3A, right) where GARP is well resolved, which contains only 6.3% of classified particles, the lasso loop of the straitjacket domain interacting with GARP becomes more flexible after binding to αvβ8, as measured from both local resolution and change of normalized B-factor based on a common reference, while local resolutions of remaining portions of L-TGF-β1/GARP are comparable in structures of αvβ8/L-TGF-β1/GARP and L-TGF-β1/GARP (Figures 3B, 3C, S3). As revealed in this best resolved structure of the trimeric complex, the arm domain of L-TGF-β becomes more stable upon binding to αvβ8, indicated by a reduction of ~15Å^2^ in B-factor from L-TGF-β/GARP alone, but the straitjacket domain, including lasso loop and the interface of GARP with mature TGF-β, becomes more flexible, with a ~15Å^2^ increase in B-factor (Figure 3D). Consequentially, destabilization of the TGF-β/GARP interface leads to progressive disappearance of L-TGF-β/GARP in the reconstructions, also reflected as progressive increase of B-factor (Figure 2F, **class 3 to 8,** S4C–D). Thus, binding to αvβ8 not only stabilizes the RGD loop and part of the arm domain that binds to the integrin, but also allosterically induces more flexibility in distal regions of the L-TGF-β1 ring, particularly the lasso loop and straitjacket. These findings suggest that such induced flexibility activates TGF-β1 (Figures 3D–E).

### Spatial conformational entropy redistribution drives αvβ8 mediated L-TGF-β activation

What drives the allosteric activation of TGF-β1? The changes between L-TGF-β1/GARP and αvβ8/L-TGF-β1/GARP are not consistent with a “classic allostery” model conceptualized as a “domino effect” of conformational changes between stable structural endpoints^36^. In the best resolved structures (Figure 3A, class 1), we observed minimal changes of L-TGF-β1 in its overall conformation after binding to integrin (GARP: RMSD 1.3Å, 3950 atom pairs; L-TGF-β1 non-integrin binding subunit A: RMSD 1.9Å, 2496 atom pairs; L-TGF-β1 integrin binding subunit B: RMSD 2.0Å, 2398 atom pairs). Rather, there are obvious changes in local resolution of reconstructed maps, and per residue B-factor in refined structures (Figure 3A–D). Indeed, conformational flexibility instead of a series of discrete conformational changes is thought to drive dynamic allostery^37,38,39,40^.

Examining allostery through a thermodynamic lens allows connecting ‘classic’ and ‘dynamic’ allostery, where any change to the protein impacts free energy through both entropy and enthalpy. It is hypothesized that dynamic allostery influences free energy, predominantly via entropic contributions^37,41^. Based on the Boltzmann equation, S=k_B_ln(W), where S is entropy, k_B_ is Boltzmann’s constant and W represents the number of microstates^42^, higher conformational dynamics equal higher conformational entropy. It has also been observed that, upon binding small molecules, peptides or DNA, proteins tend to redistribute their conformational entropy, i.e. reduce conformational entropy around the binding site and consequentially increase conformational entropy in a distal region^39,40,43,44^. As proposed previously^45,46^, spatial redistribution of conformational entropy explains dynamic allostery. Applying this concept to explain dynamic allosteric activation of L-TGF-β1, our results lead to a hypothesis whereby, upon binding to αvβ8, conformational entropy in L-TGF-β/GARP is redistributed from the αvβ8 binding site to the L-TGF-β straitjacket domain (Figure 3E), allosterically exposing mature TGF-β to TGF-βR, leading to signaling.

We designed additional experiments to test this dynamic allostery hypothesis. First, we tested whether stabilizing the L-TGF-β/GARP interface in αvβ8/L-TGF-β/GARP would change the direction of spatial conformational entropy redistribution towards αvβ8. Using the inhibitory Fab MHG8, which binds to and stabilizes the L-TGF-β1/GARP interface^9^, we determine αvβ8/L-TGF-β1/GARP/MHG8 structure. Indeed, we find the L-TGF-β1 straitjacket, including the LAP ring and integrin binding site is well-resolved but most of αvβ8, including the head domain, is unresolved confirming redistribution of conformational entropy towards the integrin (Figures 3F–H and S4E).

Following this experiment, we further tested if the direction of spatial conformational entropy redistribution can be altered by stabilizing flexible regions. We determined a cryo-EM reconstruction of L-TGF-β1/GARP in complex with full length αvβ8 (αvβ8fl) reconstituted into lipid nanodisc (αvβ8fl-nd) constraining its otherwise flexible lower legs (Figures 3I, S4F, G). Compared with class 1 of truncated αvβ8 ectodomain (αvβ8tr) bound with L-TGF-β1/GARP, this reconstruction has better resolved αvβ8 leg, but local resolution of L-TGF-β1/GARP is worse with higher B-factor (Figure 3J–L, and S4G). Together, our results suggest conformationally flexible regions in αvβ8/L-TGF-β1/GARP serve as entropic reservoirs that can be regulated or manipulated to alter direction of entropy redistribution.

To further test this directionality of conformational entropy redistribution hypothesis, we constrained L-TGF-β1/GARP into a physiologically relevant membrane and allowed it to bind αvβ8 with various amounts of constraint, ranging from none to global stabilization (Figure 3M). In this system, L-TGF-β1/GARP is expressed in the cell membrane of a transformed mink lung epithelial TGF-β responsive reporter cell (TMLC)^47^ (Figure 3N), and allowed to bind with empty nanodisc as a control (Figure 3N, panel 1), αvβ8tr without constraint (2), C-terminally clasped αvβ8tr (3), αvβ8fl-nd (4), unclasped αvβ8tr globally stabilized by immobilization (5), or C-terminally clasped αvβ8tr globally stabilized by immobilization (6). For αvβ8tr, we predict that the membrane constraint imposed on L-TGF-β1/GARP directs entropy towards αvβ8 leading to inefficient TGF-β1 activation (Figure 3M). Such constraint would be overcome by increasing the constraint imposed on αvβ8, leading to increasing the efficiency of TGF-β1 activation (Figure 3M).

Indeed, with different forms of αvβ8 showing similar binding to L-TGF-β1/GARP (Figure S4H), soluble αvβ8tr without constraint does not efficiently induce TGF-β signaling (Figure 3O). In comparison, soluble C-terminally clasped αvβ8tr, or αvβ8fl-nd more efficiently activates TGF-β signaling (Figure 3O). Global immobilized C-terminally clasped or unclasped αvβ8tr has the highest activation efficiency (Figure 3O). In this assay configuration there is no mechanical force applied to αvβ8 from the actin cytoskeleton. Thus, the mechanism of αvβ8-dependent TGF-β activation favors dynamic allostery. These experiments support the hypothesis that conformational entropy redistribution is not only sufficient but is the primary mechanism driving αvβ8 mediated L-TGF-β activation.

### Intrinsic and induced flexibility of L-TGF-β3 and L-TGF-β3/GARP

L-TGF-β3 presented by GARP is essential during development and may also play a role in immunosuppressive immunity in post-natal life^48–51^. Therefore, we next study the structure and αvβ8 mediated activation of L-TGF-β3 alone and presented by GARP.

**Figure 4.**
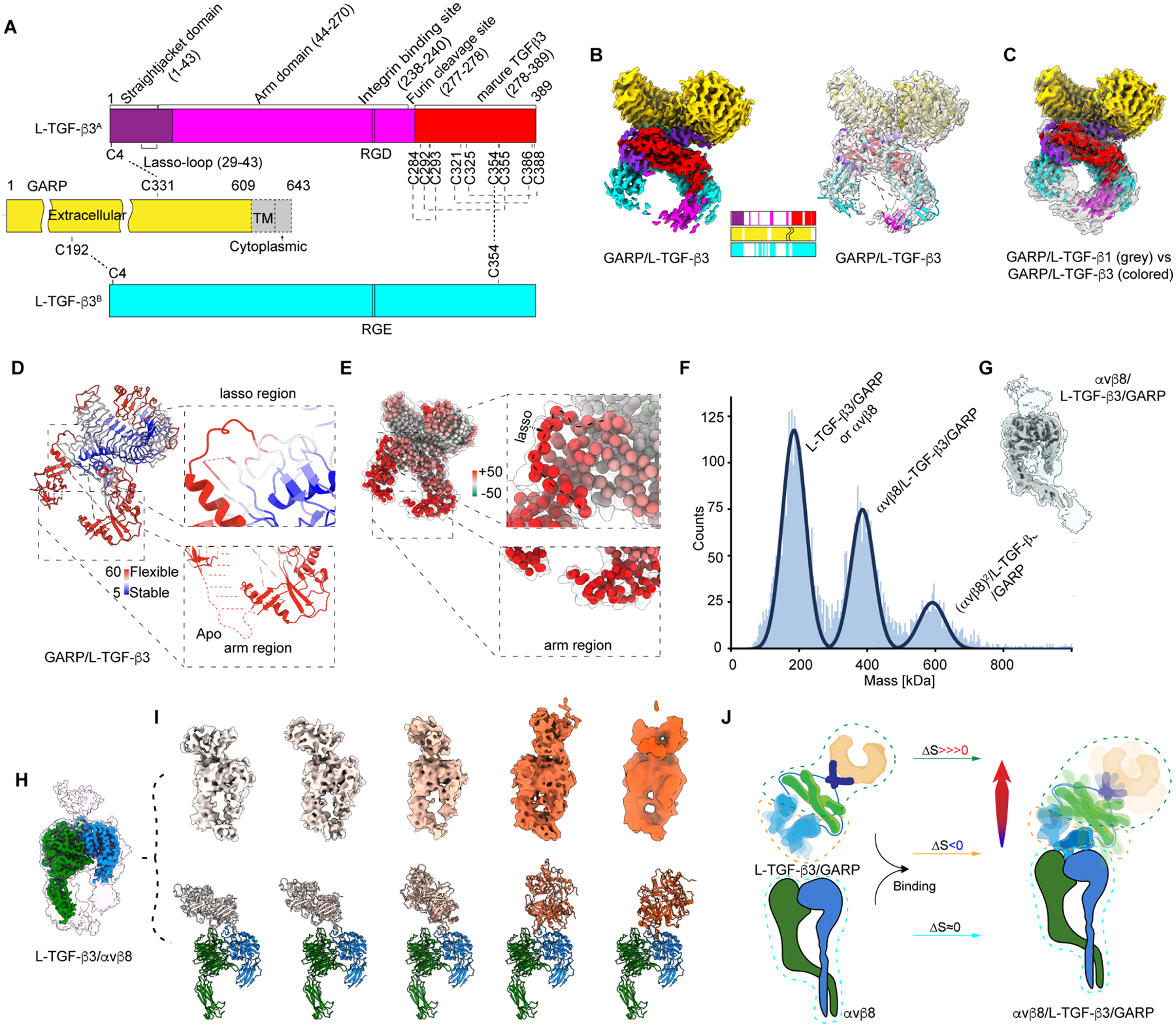
Intrinsic flexibility of L-TGF-β3/GARP leads to high basal activation of TGF-β3 **(A)** Schematic diagram of L-TGF-β3/GARP constructs, with all domains annotated and colored as in Figure 2A. **(B)** Left: Density map of L-TGF-β3/GARP with domains colored as in **(A)**. Bars are colored following convention in **(A)**, with the exception that unresolved regions are shown in white. Right: The same map (transparent) with ribbon diagram of L-TGF-β3/GARP displayed within. **(C)** Comparison of maps of L-TGF-β1/GARP (transparent grey) and L-TGF-β3/GARP (colored solid surface) shows arm domain of L-TGF-β3/GARP is more flexible than L-TGF-β1/GARP. **(D)** Ribbon diagram of L-TGF-β3 with residues colored by normalized B-factors with scale bar. Two enlarged views within dashed boxes show lasso loop (upper) and RGD containing arm domain (lower). **(E)** A consensus model of L-TGF-β1/GARP and L-TGF-β3/GARP with each Cα represented by a ball and colored with difference of normalized B-factors between L-TGF-β1/GARP and L-TGF-β3/GARP. Note B-factors of entire L-TGF-β3, particularly straitjacket (upper dashed box) and arm domains (lower dashed box), are much higher (~50Å^2^) than L-TGF-β1/GARP, indicating L-TGF-β3 is more flexible than L-TGF-β1 presented by GARP. **(F)** Mass photometry histogram: Peaks correspond to L-TGF-β3/GARP or αvβ8 alone (~190kd), L-TGF-β3/GARP with one (~390kd), or two αvβ8 integrins (~590kd). **(G)** Density map of L-TGF-β3/GARP bound with one αvβ8 at two thresholds. Disappearance of major part of L-TGF-β3/GARP indicates extensive flexibility upon binding to αvβ8. **(H)** Density map reconstructed from all particles of L-TGF-β3 bound with one αvβ8. Map displayed at two thresholds. **(I)** Upper row: 3D classification of all particles in **(H)** show flexibility of L-TGF-β3 bound with αvβ8. Bottom row: fitted atomic models of αvβ8 and L-TGF-β3 into corresponding maps shown in upper row. **(J)** Cartoon of mechanistic model of intrinsic (left) versus αvβ8-induced flexibility of L-TGFβ3/GARP (right). See also Figure S5.

Using a similar strategy as for L-TGF-β1, we purified recombinant L-TGF-β3 and the L-TGF-β3/GARP complex (Figure 4A). Single particle cryo-EM studies provided structures of L-TGF-β3/GARP (2.9Å, Figure 4B and S5A), comparable with that of L-TGF-β1/GARP (3.0Å). The portion of the straitjacket in contact with GARP almost identical in both structures (Figures 4C, S5B–D). However, L-TGF-β3 is significantly more flexible in all other regions by B-factor comparison to L-TGF-β1, particularly the arm, which contains the integrin binding site, and the portion of the straitjacket domain, including the lasso loop, that cradles the tip of mature TGF-β containing the receptor binding domain (Figure 4D and E). Such increased intrinsic flexibility suggests that L-TGF-β3 is less constrained and contains higher basal entropy than L-TGF-β1. We hypothesize that increased basal entropy facilitates exposing mature TGF-β3 to TGF-βRs even without binding to αvβ8. After integrin binding, further entropic perturbation would lead to release of mature TGF-β3 from its latent complex.

To test this hypothesis, we determined structures of αvβ8/L-TGF-β3 (2.7Å) and αvβ8/L-TGF-β3/GARP (~ 4.9–7.2Å) using L-TGF-β3 constructs where the furin cleavage site (R277A) was mutated to ensure mature TGF-β3 remained associated with the latent complex (Figure S5E–F). For image processing, we used the same procedure as applied to αvβ8/L-TGF-β1/GARP avoiding potential bias in data interpretation. Although αvβ8/L-TGF-β3/GARP is stably formed (Figure 4F) and αvβ8 density well resolved, only a small portion of L-TGF-β3 but no density of GARP is resolved (Figure 4G). To simplify structural analysis, we focused on αvβ8/L-TGF-β3 without GARP (Figures 4H, and S5F). Further classification reveals L-TGF-β3 rocks over the top of αvβ8 (Figure 4I) in a much larger range than L-TGF-β1 bound to αvβ8^22^. Indeed, in all conformational snapshots, the L-TGF-β3 straitjacket domains, including mature TGF-β3 peptides, are not resolved (Figures 4H and 4I). Overall, our structural studies of αvβ8/L-TGF-β3 and αvβ8/L-TGF-β3/GARP reveal similar but more dramatic redistribution of conformational entropy as seen in the αvβ8/L-TGF-β1/GARP (Figures 2F **class 1**), since we could not isolate any subclass with either GARP or complete L-TGF-β3 (Figure S5E–F). Thus, we conclude that intrinsic flexibility of L-TGF-β3 is further enhanced upon αvβ8 binding by a similar conformational entropy redistribution mechanism as seen with L-TGF-β1/GARP (Figure 4J).

**Figure 5.**
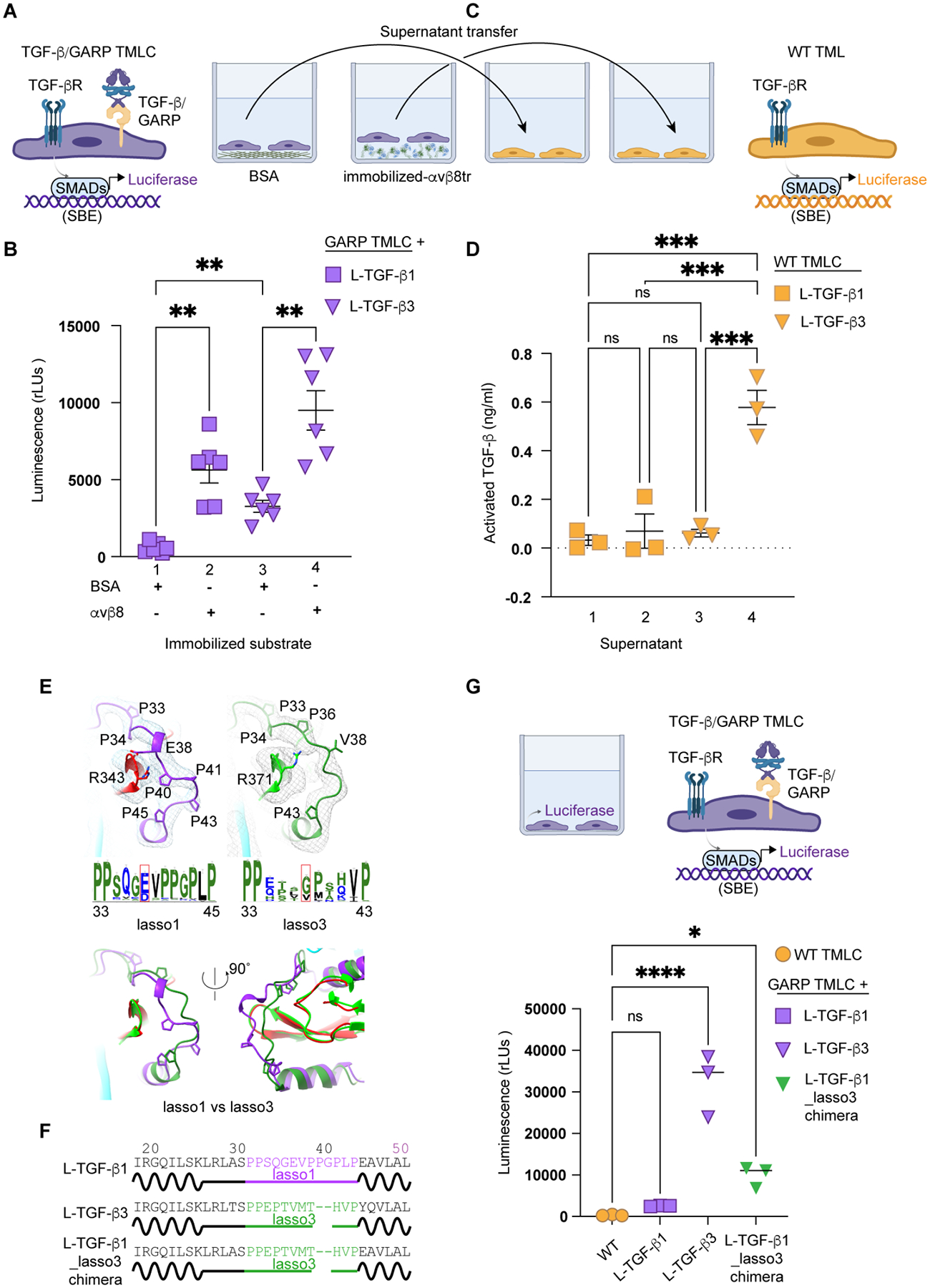
αvβ8 binding to L-TGF-β3 is sufficient to release mature TGF-β3 **(A)** Cartoon of TGF-β activation assay, with TMLC cells transfected and sorted to express equivalent levels of L-TGF-β1/GARP or L-TGF-β3/GARP on cell surfaces, cultured on either BSA or αvβ8 coated wells. **(B)** TMLC cells expressing either L-TGF-β1/GARP (purple squares) or L-TGF-β3/GARP (purple inverted triangles) were cultured overnight on the indicated substrates and luciferase activity detected and reported as luminescence in relative light units (RLU). **p<0.01 by one-way ANOVA followed by Tukey’s post-test. **(C)** Cartoon of TGF-β activation assay showing supernatants **(A)** (L-TGF-β1/GARP (yellow squares) or L-TGF-β3/GARP (yellow inverted triangles)) applied to wild-type (WT) TMLC cells detecting released TGF-β. **(D)** Following overnight culture in format shown in **(C)** luciferase activity was detected. Results (**C**, **D**) shown as active TGF-β (ng/ml). ***p<0.001 by one-way ANOVA followed by Tukey’s post-test. **(E)** Upper panel: Ribbon models and filtered densities for L-TGF-β1 and L-TGF-β3 lasso-loops. Proline residues of lasso loops, used as landmarks, indicated. Middle panel: Sequence position and species conservation (larger fonts indicate higher conservation) below ribbon models. Lower panel: Overlays of L-TGF-β1 and L-TGF-β3 models in two views illustrating lasso3 does not cover the TGF-βR2 binding site of mature TGF-β as effectively as lasso1. **(F)** Sequence alignment showing lasso region of L-TGF-β1, -β3, and chimeric L-TGF-β1 with swapped lasso of L-TGF-β3. **(G)** Lasso3 domain destabilizes L-TGF-β1/GARP. Upper: Cartoon. Lower: TMLC stably expressing GARP transfected with constructs encoding L-TGF-β1 (purple square), L-TGF-β3 (purple inverted triangles), or L-TGF-β1 with swapped lasso3 (TGF-β1_lasso3 chimera, green inverted triangles) and sorted for equivalent expression. WT TMLC (yellow circles) or L-TGF-β/GARP expressing cell lines were cultured overnight and luciferase activity reported as luminescence (RLU). *p<0.05, ****p<0.0001 by one-way ANOVA followed by Sidak’s multiple comparison test. See also Figure S6.

This presents a hypothesis that there is a threshold for flexibility of the straitjacket/lasso to allow mature TGF-β to be exposed to its receptors without being released. If so, increased intrinsic flexibility of L-TGF-β3 presented by GARP allows mature TGF-β3 to be exposed to its receptors, allowing basal activation even without integrin binding. To test this, we expressed L-TGF-β3/GARP and measured TGF-β activation using TMLC reporter cells, which, indeed, has significantly higher detectable basal TGF-β activity than that of L-TGF-β1/GARP with no basal activity (Figure 5A–B, S6). If there is similarly a threshold for flexibility of the straitjacket/lasso allowing mature TGF-β to be released, higher induced flexibility of L-TGF-β3 upon αvβ8 binding could be sufficient to cause release of mature TGF-β3 (Figure 5C). Indeed, analysis of supernatant from L-TGF-β3/GARP TMLC cells cultured on immobilized αvβ8 contained significant amounts of released TGF-β as opposed to supernatant from L-TGF-β1/GARP TMLC cells which did not (Figure 5C–D).

Why mature TGF-β3 as opposed to TGF-β1 is released from its latent complex could be explained by relative differences in intrinsic flexibility of the lasso loop, a critical determinant of latency in all TGF-β superfamily members^5,52^. A detailed comparison of sequence and structure reveals that the lasso loop of L-TGF-β3 (lasso3) is not only shorter than L-TGF-β1 (lasso1) but also less conserved in key residues interacting with mature TGF-β^53^ (Figure 5E). We thus hypothesize that lasso3 has evolved to be more flexible providing less coverage to mature TGF-β3 from exposure to its receptor allowing higher basal activity of L-TGF-β3. To test this hypothesis, we swapped the TGF-β3 lasso into TGF-β1 (L-TGF-β1_lasso3) (Figure 5F) and observed significantly increased basal activation of TGF-β1 although not to the level of wild type L-TGF-β3 (Figure 5G and S6). Together, we conclude that levels of conformational entropy of the arm, straitjacket and lasso domains, as well as the structure of lasso loop, are key to maintaining latency, exposure, or release of mature TGF-β.

### Functional consequences of TGF-β release from αvβ8 mediated L-TGF-β activation.

**Figure 6.**
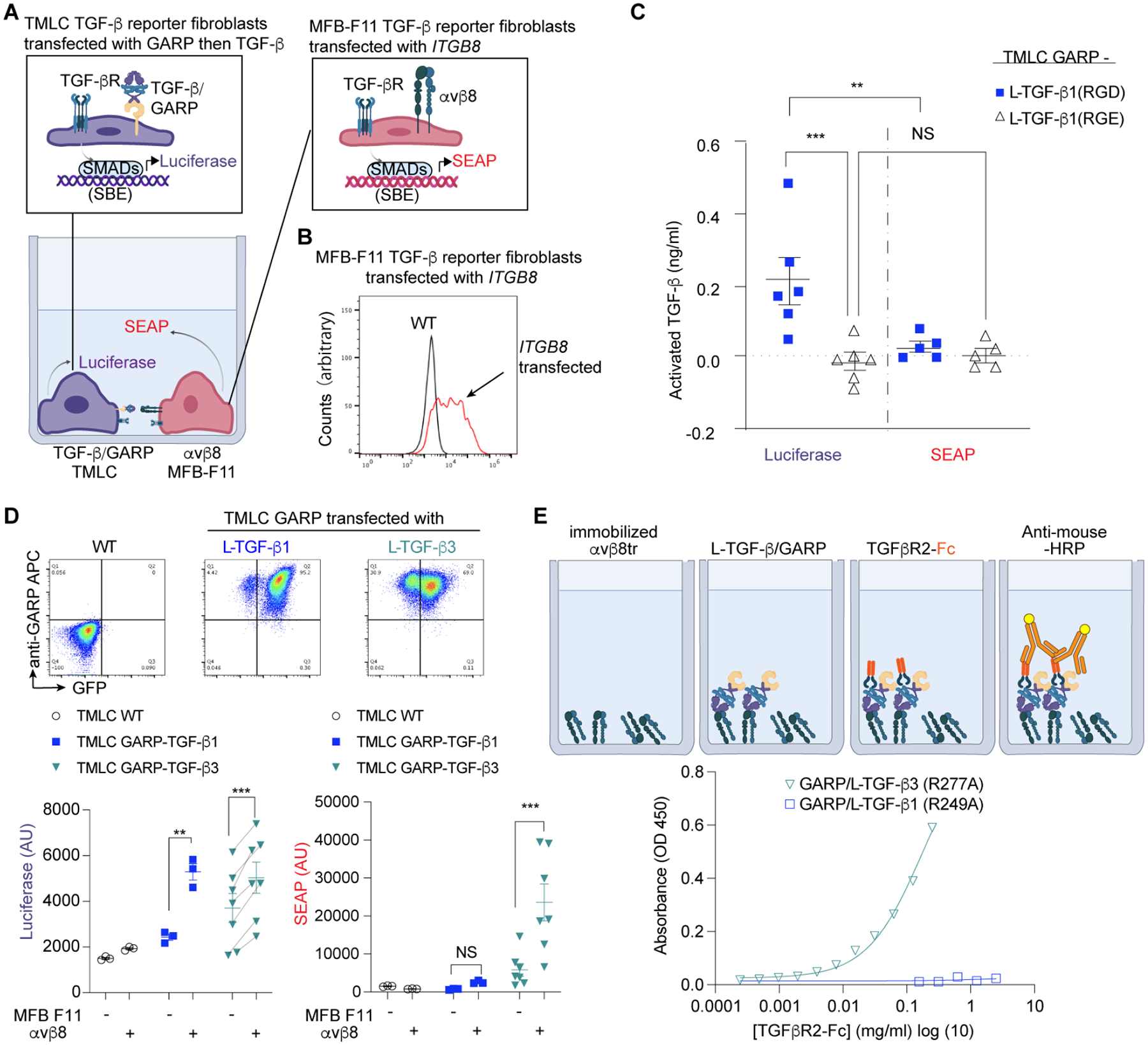
Intrinsic and integrin-induced entropy of L-TGF-β determines signaling directionality **(A)** Carton of design of dual TGF-β reporter system, TMLC (purple) and MFB-F11 (red), are co-cultured (left). Shown are stably expressed reporter constructs with SMAD-binding elements (SBE) driving indicated reporter proteins, luciferase or secreted alkaline phosphatase (SEAP). **(B)** MFB-F11 reporter cells stably transduced with an integrin β8 (*ITGB8*) expression construct sorted for high αvβ8 expression, using an anti-β8 antibody. Histogram demonstrates expression of αvβ8 only seen in *ITGB8* transfected (red curve), not non-transfected (NT) cells (black curve). **(C)** Mixing αvβ8 expressing MFB-F11 cells with L-TGF-β1/GARP presenting TMLC cells is required and sufficient to activate TGF-β signaling pathway on L-TGF-β1/GARP expressing cells but not αvβ8 expressing cells. L-TGF-β1(RGD)/GARP TMLC (filled squares), L-TGF-β1(RGE)/GARP TMLC (open triangles, characterized as Figure S3B). Results (vertical axis) normalized against standard TGF-β activation curve. **p<0.01, ***p<0.001 by one-way ANOVA followed by Sidak’s multiple comparison test. **(D)** Upper: Transfection of TMLC cells stably expressing GARP and co-transfected with either wild-type L-TGF-β1 or L-TGF-β3 IRES GFP plasmids. Surface expression of L-TGF-β1 and L-TGF-β3 are equivalent. Lower: Co-culture of wild type MFB-F11 (−), or αvβ8 transfected (+) MFB-F11 cells (indicated below graph) with wild type TMLC (black circles), or TMLC expressing L-TGF-β1/GARP (blue filled squares) or L-TGF-β3/GARP (green triangles). Left: TMLC cells have significant basal levels of active TGF-β3 even when cultured without αvβ8-expressing MFB-F11, but further increased by coculture with αvβ8-expressing MFB-F11 cells. Right: Increased SEAP only seen when TMLC L-TGF-β3/GARP cells are cocultured with αvβ8-expressing MFB-F11. Results shown as arbitrary light units (AU). **p<0.01, ***p<0.001 by one-way ANOVA followed by Sidak’s multiple comparison test, with the exception that results for L-TGF-β3/GARP cells on wild type MFB-F11 or MFB-F11 αvβ8 expressing cells are shown as a paired t-test. **(E)** TGF-βR2 binds more efficiently to αvβ8 bound L-TGF-β3/GARP compared to L-TGF-β1/GARP. Upper: cartoon showing sequential immobilization of αvβ8 ectodomain, binding of L-TGF-β1 or -β3/GARP complexes, TGF-βR2-Fc (mouse-Fc), and anti-mouse-HRP. Lower: representative experiment (n=3) shown with varying concentrations of TGF-βR2-Fc, signal reported as OD_450_.

Under the physiological conditions where αvβ8-mediated TGF-β activation occurs in vivo, αvβ8 is presented by one cell, but L-TGF-β is presented on the cell surface of a contacting cell^27^. In this scenario, αvβ8 mediated TGF-β activation could result in bidirectional signaling to both cells if TGF-β was released (paracrine), or only in unidirectional signaling on the immune cell presenting TGF-β cell if not released (autocrine). To test whether such αvβ8-mediated directional TGF-β activation occurs, we devised an *in vitro* co-culture model system where the integrin αvβ8 is expressed by TGF-β1 null embryonic fibroblasts (MFB-F11) stably expressing a TGF-β responsive secreted alkaline phosphatase (SEAP) reporter construct^54^, and TGF-β is expressed on the surface of TMLC cells (Figure 6A–C). The MFB-F11 reporter cells are highly sensitive to exogenous TGF-β, indicating possession of the full complement of TGF-β receptors and downstream signaling apparatuses^54^. When co-cultured with L-TGF-β1/GARP expressing TMLC TGF-β reporter cells, SEAP in cell supernatants reports TGF-β signaling from αvβ8 expressing cells while luciferase measured from cell lysates reports TGF-β signaling from L-TGF-β1/GARP TMLC cells (Figure 6A–C). Since MFB-F11 cells are TGF-β deficient, the only cellular source of TGF-β1 in system is from L-TGF-β1/GARP expressing TMLC TGF-β reporter cells.

Co-culture of αvβ8 expressing MFB-F11 reporter cells with L-TGF-β1/GARP expressing TMLC reporter cells results in autocrine signaling since only luciferase is detected (Figure 6A–C). Such exclusivity of autocrine signaling can be attributed to insufficient flexibility of straitjacket and lasso loops of L-TGF-β1 allowing mature TGF-β1 to be released but sufficient to be exposed within the latent ring to bind to TGF-βR2 after αvβ8 binding. The next question is whether directionality of L-TGF-β3 activation by αvβ8 is different than L-TGF-β1, since mature TGF-β3 is released upon αvβ8 binding (Figure 5C, D). Indeed, both autocrine and paracrine TGF-β3 signaling are observed since luciferase and SEAP are detected (Figure 6D). We hypothesize that within L-TGF-β, mature TGF-β3 compared to TGF-β1 would be more accessible to TGF-βR2, the first receptor binding mature TGF-β to initiate signaling^55^.

To test whether TGF-βR2 binds mature TGF-β when exposed within L-TGF-β complexes, we performed TGF-βR2 binding assays to immobilized αvβ8 bound L-TGF-β3 or L-TGF-β1/GARP complexes. In these systems, the furin cleavage site between the mature TGF-β and LAP are mutated at analogous positions as in *tgfb1*^*R278A/R278A*^ mice, and thus mature L-TGF-β cannot be released. Consistent with our structural analysis and TGF-β activation assays, we observed more robust complex formation between TGF-βR2 and L-TGF-β3/GARP than to L-TGF-β1/GARP when bound to immobilized αvβ8 (Figure 6E).

## Discussion:

### Physiological role of TGF-β1 activation without release

TGF-β1 plays major roles in mammalian biology from embryo implantation through the entire lifespan. For all roles, the dogma is TGF-β release is required for both autocrine and paracrine function^3^. This view is reinforced by numerous biochemical and structural experiments^5,53,55–58^, but is challenged by our previous study of αvβ8/L-TGF-β1 predicting mature TGF-β1 can be sufficiently exposed to bind to its receptors within L-TGF-β1 without release^22^.

Here, we provide definitive evidence that mature TGF-β without release supports autocrine signaling. In mice mature TGF-β1 covalently bound to LAP induces sufficient signaling to support immune function, since founders have so far survived 7 months without early immune lethality associated with global TGF-β1 deficiency^9,59–61^. Our ability to generate live births from homozygous *tgfb1*^*R278A/R278A*^ intercrosses in the complete absence of wild type maternal TGF-β1 from conception to adulthood, provides definitive evidence that paracrine release of mature TGF-β1 is not essential either for development or for early immune function.

Our results differ from previous reports where mice with furin conditionally deleted in T-cells develop delayed organ inflammation similar to mice with conditional deletion of TGF-β1 in T-cells^20^. However, furin potentially cleaves hundreds of substrates other than TGF-β1^62^, and likely has effects independent of TGF-β1. Mice with *tgfb1* conditionally deleted in T-cells lack autocrine TGF-β1 signaling by T-cells, whereas autocrine TGF-β1 signaling is preserved in *tgfb1*^*R278A/R278A*^ mice. Confirmation that autocrine TGF-β1 signaling without release is sufficient to prevent autoimmunity not only validate our structure-based approach to study the L-TGF-β activation mechanism but demonstrate the power of cryo-EM to reveal structural mechanisms of flexible proteins that would otherwise have been unanticipated.

### L-TGF-β activation driven by conformational entropy redistribution

The concept of conformational entropy redistribution, where entropy reduces around the ligand binding site and increases at distant sites is derived from conformational ensembles quantitatively characterized from structures obtained by X-ray crystallography or NMR spectroscopy of relatively small proteins^44,46^. With single particle cryo-EM, protein conformational dynamics correlate with local resolutions of reconstructed structures, allowing exploration of conformational entropy redistribution in much larger and complex systems. Redistribution of conformational entropy also explains dynamic allosteric communication from ligand binding sites to distant sites without involving propagation of discrete stable conformational changes^38,46^.

Here, we used cryo-EM to examine conformational entropy redistribution in large and multi-component protein complex, αvβ8/L-TGF-β/GARP, where all components have been shown to be highly flexible in our previous^22,28^ and current studies. By characterizing changes of conformational flexibility of different regions of L-TGF-β/GARP induced by integrin αvβ8 binding, we show conformational entropy redistribution is the underlying dynamic allostery mechanism of αvβ8 mediated L-TGF-β activation.

**Figure 7.**
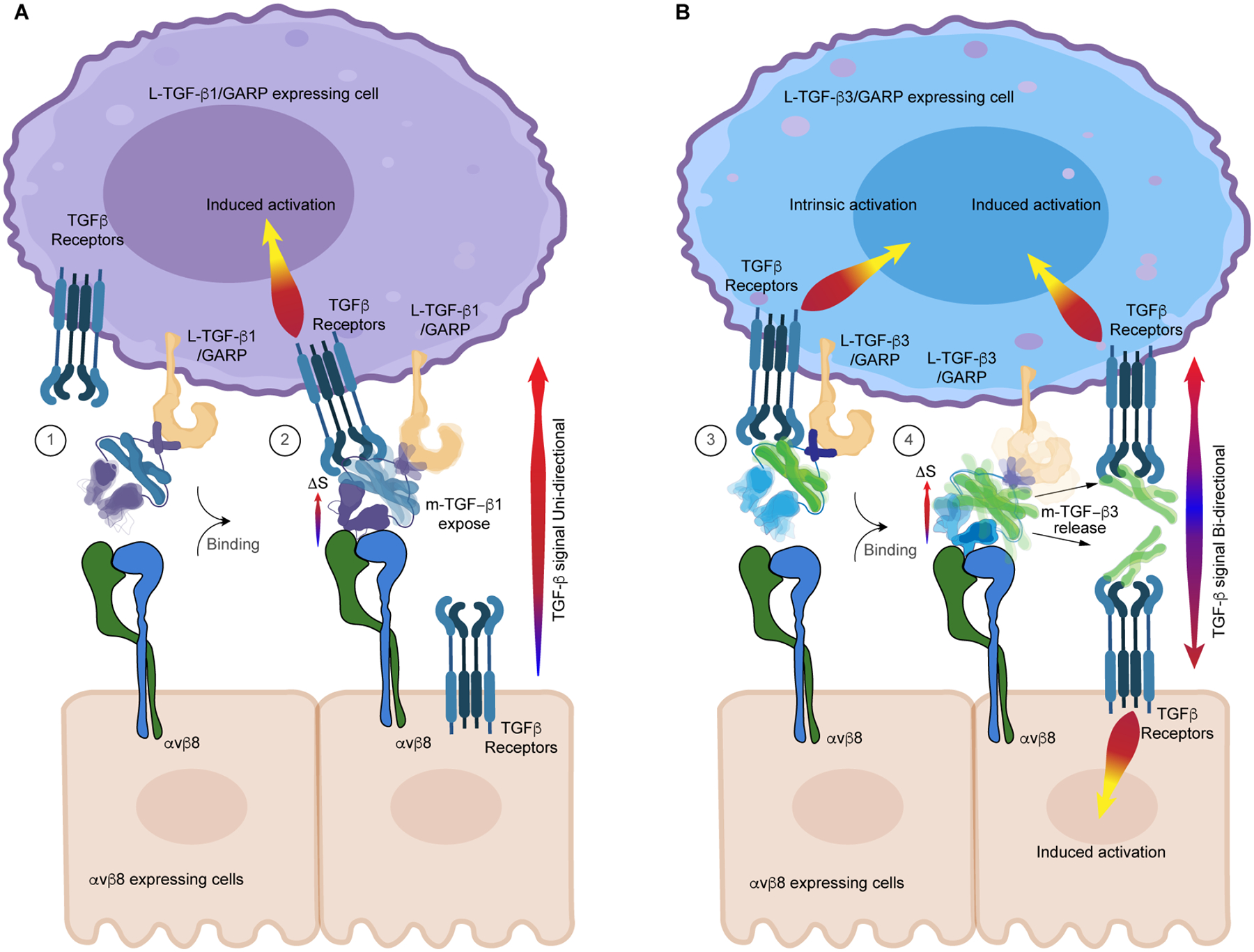
Dynamic entropy-based allosteric model of TGF-β activation **(A)** Cartoon of intrinsic and integrin-induced TGF-β1 activation. 1) L-TGF-β1/GARP has relatively low basal entropy in straitjacket/lasso, insufficient to trigger significant signaling. 2) Binding αvβ8 stabilizes arm domain but redistributes sufficient entropy to expose mature TGF-β1 to TGF-βRs to trigger signaling. **(B)** Cartoon of intrinsic and integrin-induced TGF-β3 activation. 3) TGF-β3/GARP has relatively high levels of basal entropy in straitjacket/lasso sufficient to expose mature TGF-β3 to TGF-βRs allowing basal L-TGF-β3 constitutive autocrine signaling. 4) Binding to αvβ8 stabilizes the arm domain redistributing entropy exposing mature TGF-β3 to TGF-βRs, sufficient for paracrine release of mature TGF-β3 for bidirectional signaling to L-TGF-β3 presenting, and αvβ8 expressing cells.

Specifically, we demonstrate that intrinsic flexibility, the basal conformational entropy of L-TGF-β complexes, controls TGF-β latency. In the case of the fully latent L-TGF-β1, the straitjacket and lasso loops are relatively stable (Figure 7A, panel 1), as opposed to the partial latent L-TGF-β3, where the same domains are flexible (Figure 7B, 3). Binding to αvβ8 stabilizes the flexible RGD loop on the arm domain of both L-TGF-β1 and -β3 and reduces local conformational entropy. Spatial redistribution of this entropy towards the straitjacket enhances flexibility of the respective lasso loops. For L-TGF-β1, lower basal conformational entropy results in less entropy redistribution towards the lasso domain, insufficient to release mature TGF-β but sufficient to expose it to its receptor (Figure 7A, 2). Without being released, TGF-β is restricted to autocrine signaling. In contrast, because L-TGF-β3 has higher basal conformational entropy, αvβ8 binding results in more entropy redistribution towards the lasso domain resulting in L-TGF-β3 passing a flexibility threshold sufficient for releasing mature TGF-β3 (Figure 7B, 4). Released TGF-β3 is capable of both autocrine and paracrine signaling to either αvβ8 or L-TGF-β3/GARP presenting cells.

### Physiological relevance of dynamic allostery in the activation of the TGF-β3/GARP complex

TGF-β3, as opposed to TGF-β1, is required for palatogenesis^13,16,63,64^. Our structural and cell-based findings of αvβ8/L-TGF-β3/GARP complex are likely physiological relevant since GARP and TGF-β3 form a covalent complex^9,50^, GARP and TGF-β3^50^ colocalize to critical regions involved in palatogenesis, their respective genetic deficiencies in mice and/or humans lead to cleft palate^16,49,50,65^, and human genetic cleft palate syndromes result from missense mutations in the furin or the RGD sequence of *TGFB3*^14,66^. Interestingly, the cleft palate phenotype in *itgb8* null mice is only seen in a subset of live births^67,68^. We speculate that the intrinsic flexibility of L-TGF-β3 and exposure of mature TGF-β3 provides sufficient basal TGF-β signaling for palatogenesis even without integrin αvβ8 binding.

### Broader implication of dynamic allostery mechanism in macromolecular complexes

Our data using the multicomponent αvβ8/L-TGF-β1(-β3)/GARP model system provide evidence that redistribution of conformational entropy is a mode of allosteric regulation in a highly dynamic system. Protein dynamics are quantified as conformational entropy via the Boltzmann equation^42^. It has been demonstrated that protein dynamics can tune protein function without involving discrete conformational changes^38^. However, until very recently, examples of such dynamics were limited to side chain rotamer ensembles by X-ray crystallography or methyl or amine group dynamics of small proteins by NMR spectroscopy^44^. The αvβ8/L-TGF-β/GARP provides a case study of a tunable functional endpoint (i.e. activation) correlating with protein dynamics in a relatively large complex. It is likely that dynamic allostery is a widespread mechanism to tightly regulate protein function, yet underappreciated, since methodology to decipher this new dimension in macromolecular protein function is only beginning to be applied. Single particle cryo-EM is one valuable tool to directly visualize conformational dynamics in larger protein complexes. Combining it with other technologies, such as hydrogen-deuterium exchange mass spectrometry and/or molecular dynamics simulations, etc., it is reasonable to anticipate dynamic allostery driven by conformational entropy redistribution will be found to play important mechanistic roles in many biological systems.

### Intrinsic conformational entropy in latency of the TGF-β superfamily

The three TGF-β isoforms are thought to be completely latent, along with a few others in the larger TGF-β superfamily (i.e. GDF8, and GDF11)^5,52,69,70^. The high basal activity of L-TGF-β3 was unexpected, which we attribute to the increased intrinsic entropy of straitjacket and lasso loops compared to TGF-β1. We propose the relative intrinsic entropy of the straitjacket and lasso is a general evolutionary strategy controlling the degree of latency of TGF-β superfamily members. Thus, as with L-TGF-β3, latency is clearly not absolute, but rather determined by the degree of intrinsic conformational entropy. Extending this concept to non-latent TGF-β superfamily members with available structures, all have highly flexible straitjacket and lasso loops, such as BMP9, BMP10 and ActivinA suggesting they have very high levels of intrinsic entropy allowing their growth factors to be freely exposed to receptors without requiring release (Figure S7). Overall, our data supports an alternative hypothesis where extent of latency is a continuum controlled by levels of intrinsic entropy of the straitjacket, which when sufficiently high allows receptors to bind exposed receptor binding domains of mature growth factors while still within the prodomain complex. Amongst the TGF-β superfamily, L-TGF-β1, with its relatively low entropy appears to be an exception, rather than the rule.

### Therapeutic implications

The general concept of latency of TGF-β activation is binary, it is either latent or active. The binary dogmatic view has led to therapeutic approaches targeting released paracrine mature TGF-β and have efficacy and safety issues^71–76^. The architecture and flexibility of αvβ8/L-TGF-β/GARP suggests L-TGF-β/GARP antibody binding epitopes are highly flexible and unstable, and multiple steric clashes limit access of TGF-βR traps, or antibody inhibitors to TGF-β or TGF-βRs within the complex. Indeed, we have observed poor inhibitory activity of antibody inhibitors to TGF-β, TGF-βRs, L-TGF-β1/GARP or TGF-βR traps for αvβ8-mediated activation of TGF-β^27^. Thus, it is not surprising that immuno-oncology clinical trials using approaches that target paracrine released TGF-β have been disappointing due to lack of efficacy^77^. Our results predict antibodies stabilizing L-TGF-β might also face similar efficacy challenges in clinical trials if the activation mechanism is αvβ8-dependent^9,78,79^.

Mechanistic insights revealed from our study suggest why TGF-β function can be highly context dependent, given dynamic allostery determines where and when TGF-β is activated, whether it signals as an autocrine factor while remaining associated with the latent complex or is released, and ultimately whether it mediates paracrine signaling. Such a mechanism determines if TGF-β primarily directs signaling to TGF-β-presenting or integrin-expressing cells, and cells in proximity or at a distance. Thus, targeting TGF-β activation is highly complex, but such complexity offers opportunities for targeting context-dependent directional TGF-β activation, which can be achieved at multiple levels either through targeting basal entropy, entropic redistribution, or release. Importantly, entropy redistribution can be manipulated to occur in different directions, as demonstrated by our findings (Figure 3F–O). It remains to be determined how targeting entropically driven mechanisms will affect different pathologic scenarios. Overall, our results provide a structural framework for developing therapeutic approaches to inhibit context-specific functions of different TGF-βs and argue against one-size-fits-all targeting strategies.

### Limitations of Study

The *tgfb1*^*R278A/R278A*^ mouse model is early in establishment and full immune characterization is ongoing. Thus, we cannot exclude delayed effects on tissue inflammation due to lack of paracrine release of mature TGF-β1 as mice age further.

Despite providing a conceptual framework for understanding the mechanism of dynamic allostery, our current description of conformational entropy redistribution is only partially quantitative. More comprehensive quantitative descriptions of large-scale conformational entropy redistribution require major advancements of current methodologies.

## STAR METHODS

### RESOURCE AVAILABILITY

#### Lead Contact

Further information and requests for reagents may be addressed to Yifan Cheng (yifan.cheng@ucsf.edu).

#### Materials Availability

All new materials generated in this manuscript, including mice, antibodies, cell lines and plasmids, are available on request from the lead contact with a completed Materials Transfer Agreement.

#### Data and Code Availability

- All cryo-EM density maps, coordinates for the atomic models and local-refined maps generated in this study have been deposited and are publicly available. Accession numbers (EMDB, PDB IDs) are listed in the key resources table.
- No new code was included in this study.
- Any additional information required to reanalyze the data reported in this paper is available from the lead contact upon request.

### EXPERIMENTAL MODEL AND STUDY PARTICIPANT DETAILS

#### Mice

129SX1V/J × C57BL/6 *Tgfb1 fl/+* mice (Jax) with loxP3 sites flanking *tgfb1* exon 3 were crossed to 129X1SV/J × C57BL/6 *Rosa 26*-cre mice (Ozgene) to create *tgfb1*^*+/−*^ mice which were intercrossed to produce *tgfb1* ^−/−^ mice^33,94^. *Tgfb1* mice with a mutation in the furin cleavage site M13177.1c.1184-5AG>GC (p.Arg278Ala) were created at Ozgene (Perth, WA, Australia) using a conditional knock-in strategy on a C57Bl/6 background (Figure S2). The targeting vector consisting of 5’ homology arm containing *tgfb1* exon 3 followed a murine *tgfb1* cDNA minigene spanning exon 4–7, followed by a neomycin resistance cassette flanked by flippase recognition target sites (Frt), and the entire minigene and neo cassette flanked by loxP3 sites, which was inserted into intron 3, which was followed by exon 4, intron 4 and exon 5 with a mutation (AC to GC) in R278 to change the furin cleavage motif ^275^RHRR^278^ to ^275^RHRA^278^ (R278A) followed by a 3’ homology arm. Successful targeting and germline transmission was followed by excision of the Frt flanked neo cassette to create a conditional KI (cKI) allele. Upon cre-mediated recombination, the loxP3 wild-type *tgfb1* exon 4–7 cDNA minigene can be excised and replaced with the *tgfb1* R278A mutant allele (Figure S2). C57BL/6 heterozygous (*tgfb1 cKI/+*) or homozygous (*tgfb1 cKI/cKI)* mice were crossed to C57BL/6 *Rosa 26*-cre mice (Ozgene). The resulting C57BL/6 KI/WT mice were mated to WT 129X1SV/J mice to generate 129X1SV/J × C57BL/6 *tgfb1*^*R278A/+*^ mice. Alternatively, 129X1SV/J × C57BL/6 *tgfb1 cKI* ^*R278A*^ */+* mice were crossed to 129X1SV/J × C57BL/6 ^*Rosa 26*-cre*/WT*^ mice (Ozgene) to create knock-in *tgfb1*^*R278A/+*^ mice. Initial genotyping was performed using tail genomic DNA isolated and genotyped by PCR (Kapa) using primers TGFb1 1F, and TGFB1 KI/cKI 4R which produce a 654 bp band for the KI and 620 bp band for the WT allele. Subsequent genotyping was performed using WT or KI specific primers (TGFb F WT only or TGFb F KI only, paired with TGFb WT/KI rev) *Tgfb1*^*R278A/+*^ mice breeding pairs from either strategy were intercrossed to produce *tgfb1*^*R278A*^*/tgfb1*^*R278A*^ mice. Mice were screened for flippase (Flp) rosa 26-cre using a primer mixture (ROSAWT F, ROSAFlp F, ROSAcre F, ROSA R) and flp + mice removed from the colony. WT, *tgfb1*^*R278A*^*/tgfb1*^*R278A*^or *tgfb1*^*R278A*^*/tgfb1*^*WT*^ mice heterozygous or null for Rosa 26-cre and null for Flp were intercrossed and used for survival experiments. Live litters containing KI/KI mice were produced from intercrossing *tgfb1*^*R278A*^*/tgfb1*^*R278A*^ or *tgfb1*^*R278A*^*/tgfb1*^*WT*^ mice, or crossing *tgfb1*^*R278A*^*/tgfb1*^*R278A*^ to *tgfb1*^*R278A*^*/tgfb1*^*WT*^.To confirm mutant mRNA production from the KI and knock-out alleles, RNA was extracted from tail clippings, cDNA synthesized and amplified using the respective primer pairs tgfb1 Ex3/4 cDNA F and tgfb1 ex 6/7 cDNA R, and tgfb1 Ex1/2 cDNA F and tgfb1 Ex4/5 cDNA R and the products sequenced. To confirm the absence of mature TGF-β1 protein in the KO and absence of released mature TGF-β1 in the KI mice, immunoblots were performed using an antibody to mouse mature TGF-β1 (Abcam, ab179695). Serum was precleared 3 times with Protein G Sepharose beads to deplete IgG prior to immunoblotting. Spectral flow cytometry was performed on peripheral blood, or spleen from *tgfb1*^−/−^, KI/KI (*tgfb1*^*R278A*^*/*tgfb1^R278A^) or appropriate age and littermate matched controls (WT/WT. WT/KO or WT/KI). Histologic analysis of various organs were scored on an inflammation scale of 0–3 (0 = no inflammation; 1 = scattered lymphocytes infiltrating into tissues; 2 = distinct aggregates of lymphocytes infiltrating into tissues; 3 = diffuse inflammation infiltrating tissue in dense sheets of lymphocytes) (Table S1). Total inflammation score represents the sum of all individual organ inflammation scores.

#### Cell lines

Transformed mink lung TGF-β reporter cells (TMLC)^47^ were a gift from J. Munger (New York University Medical Center, New York, NY, USA) and were stably transfected with L-TGF-β1 (RGD/RGD), L-TGF-β1 (RGD/RGE), L-TGF-β1 (RGE/RGE), L-TGF-β3 (RGD/RGD), L-TGF-β3_lasso3 with or without GARP, as previously described^22^. TMLC cells were grown in DMEM + 10% FBS + penicillin-streptomycin + amphotericin B, cultured at 37°C in a humidified incubator, 5% CO2.

MFB-F11 cells were a gift from Tony Wyss-Coray (Stanford University, School of Medicine). MFB are a mouse fibroblast line from *tgfb1*−/− mice which were stably transfected with an SBE-SEAP reporter cassette with a hygromycin resistance cassette and clone F11 isolated by limiting dilution^54^. MFB-F11 cells were stably transduced with human *ITGB8* construct using retroviral particles from the Phoenix amphotropic viral packaging cell line (Phoenix-AMPHO, ATCC). MFB-F11 cells were maintained in DMEM + 10% FBS + penicillin-streptomycin + amphotericin B, cultured at 37°C in a humidified incubator, 5% CO_2_. β8 expression was maintained by supplementing basal media with 5 μg/mL puromycin. Phoenix cells were maintained in DMEM + 10% FBS + penicillin-streptomycin + amphotericin B, cultured at 37°C in a humidified incubator, 5% CO_2_.

#### DNA constructs

The following cDNA constructs were used: β8 cDNA pBABE puro, αvfl pcDM8, αvtr pcDM8, β8tr pcDNA1neo, β8fl pcDNA1neo^22,28^; pLX307 hTGF-β1 IRES2 EGFP (h preceding protein name indicates human from here forward) was constructed from TGF-β1_pLX307 (Plasmid #98377, AddGene) to remove a c-terminal V5 tag by cloning a PCR fragment created with primers (5’- caggtgtcgtgaggctagcatcg-3’, and 5’-gcgccactagtctcgagttatcag-3’) which was used as a backbone to generate pLX307 hTGF-β1 RGE_IRES2 EGFP puro, pLX307 hTGF-β1 RGD_R249A_IRES2 EGFP puro, pLX307 hTGF-β1 RGE_R249A_IRES2 EGFP puro, as described^22^, L-TGF-β1_RGD_Lasso3 (where the A31-L44 in lasso1 loop was swapped with T31-V42 from the L-TGF-β3 lasso3 loop) was made by splice overlap extension PCR using the primers (5’-ccatttcaggtgtcgtgaggc-3’, 5’-ccctgagccaacggtgatgacccacgtccccgaggccgtgctcgc-3’, 5’- gtcatcaccgttggctcaggggggctggtgagccgcagcttggacag-3’, 5’-tggcgtagtagtcggcctc-3’). HA-GARP pcDNA3^78^ was a gift from Sophie Lucas (Institut de Duve, Belgium), HIS SBP human GARP (hGARP) pcDNA6 was made using an N-terminal rat albumin signal peptide-His Tag-Strepravidin binding protein-HRV 3C protease tag (HIS SBP) from HIS SBP tagged porcine L-TGF-β1 pcDNA6^22^ as a template using primers (5’-ctctgatatcccaagctggctagccacc-3’, 5’-cagggcactttgtcttggtgaggaccctgaaacagcacctc-3’) and joined by splice-overlap extension to a fragment amplified from HA GARP pcDNA6^95^ using primers (5’-ttagaggtgctgtttcagggtcctcaccaagacaaagtgccctg-3’, and 5’-ccgctgtacaggctgttccc-3’), HIS SBP hGARP tr pcDNA6 was made by ligation of a PCR amplified fragment (HIS SBP hGARP fl pcDNA6 as a template with the primers 5’-agggccgtgtggacgtgg, and 5’-tctcctcgagttatcagttgatgttcttcagtccccccttc-3’), HIS SBP hGARP tr pcDNA6 SpyCatcher was generated from HIS SBP-GARP tr pcDNA6 by gapping into the XhoI/XbaI cut plasmid a PCR fragment amplified from addgene-plasmid-133447 (SpyCatcher) using primers (5’-ggggactgaagaacatcaacatgtcgtactaccatcaccatc-3’; 5’-ggcttaccttcgaagggcccttagctaccactggatccagta-3’) using the Gibson Assembly Cloning Kit (NEB #E5510S). The entire human open reading frame and IRES RED cassette was transferred using PmeI/SpeI from pLVE-hTGFB3-IRES-RED (Plasmid #52580, addgene) to replace the TGF-β1 reading frame and IRES GFP (ClaI/Klenow, SpeI) into TGF-β1_pLX307 to create hTGF-β3 IRES RED, hTGF-β3 R277A IRES RED was made using splice overlap extension using PCR products (5’-ccatgtcacacctttcagccc-3’, 5’-gtccaaagccgccttcttcctctg-3’; 5’-cagaggaagaaggcggctttggac-3’, gtgttgtacagtcccagcacc), hTGF-β3 RGE_R277A_IRES RED was made using splice overlap extension using PCR products (5’- ggcgccccagttctccacgg-3’, 5’-ggcgccccagttctccacgg-3’; 5’-ggagaactggggcgcctcaag-3’, 5’-gtccaaagccgccttcttcctctg-3’; 5’-cagaggaagaaggcggctttggac-3’, 5’-gtgttgtacagtcccagcacc-3’), hTGF-β3 RGE_IRES RED was made using splice overlap extension using PCR products (5’- ggcgccccagttctccacgg-3’, 5’- gtccaaagccgccttcttcctctg-3’; 5’- ggagaactggggcgcctcaag-3’, 5’-gtgttgtacagtcccagcacc-3’), hTGF-β3 IRES GFP, hTGF-β3 RGE_IRES GFP, hTGF-β3 R277A_IRES GFP and hTGF-β3 RGE_R277A_IRES GFP were made by cloning in the PCR fragment generated using TGF-β1_pLX307 as a template (5’- ctctacgcgtactagtggcgcgccgg-3’, 5’-ttacttgtacagctcgtccatgcc-3’) and cloning into hTGF-β3 IRES RED, hTGF-β3 R277A_IRES RED, hTGF-β3 RGE_IRES RED and hTGF-β3 RGE_R277A_IRES RED, SBP HIS L-TGF-β3, SBP HIS L-TGF-β3 RGE, SBP HIS L-TGF-β3 R277A, SBP HIS L-TGF-β3 R277A_RGE, SBP HIS L-TGF-β3 C4S, SBP HIS L-TGF-β3 C4S RGE, SBP HIS L-TGF-β3 C4S R277A, and SBP HIS L-TGF-β3 C4S R277A_RGE all in pcDNA6 were made using splice overlap extension PCR to amplify the rat albumin signal peptide-HIS SBP-HRV 3C protease tag from HIS SBP L-TGF-β1 pcDNA6 using primers specific for HIS SBP L-TGF-β3 C4WT (5’-gactcactatagggagacccaagctgg-3’, 5’- gtccaaggtggtgcaagtggacagggaccctgaaac-3’; 5’-ctgtccacttgcaccaccttggac-3’, 5’- ggtgagcctaagcttgctcaagatctg-3’) or HIS SBP L-TGF-β3 C4S: 5’- gactcactatagggagacccaagctgg-3’, 5’- gtccaaggtggtgctagtggacagggaccctgaaac-3’; 5’- ctgtccactagcaccaccttggac-3’, 5’- ggtgagcctaagcttgctcaagatctg-3’), and ligating the corresponding spliced products into hTGF-β3 IRES GFP, hTGF-β3 RGE_IRES GFP, hTGF-β3 R277A_IRES GFP and hTGF-β3 RGE_R277A_IRES GFP. All cDNA constructs were verified by sequencing. TGF-βR2-Fc was previously described^27^.

### METHOD DETAILS

#### Antibody isolation, characterization, and production

The following antibodies were used: and anti-β8 clone F12, which is high-affinity derivative of the parental clone C6D4^80^ created by combining rational structure based directed evolution to create a mutagenic Vh and Vl domain library focused on amino acids to optimize the binding interface, displayed on the surface of yeast, and after multiple rounds of sorting, isolation and subcloning into murine IgG2a format, as described^80^, produced in ExpiCHO cells.

#### Expression and purification of proteins for functional assays and single particle cryo-EM

Secreted ectodomain of αvβ8 integrin was produced by transfecting ExpiCHO cells with integrin constructs^22^ using the manufacturer’s protocol. Specifically, after 5 days growth, cell culture was centrifuged to collect supernatant, which was filtered through a PES (polyether sulfone) membrane, 0.2 μm pore size (Millipore). Protein purification is carried out by affinity chromatography using a column packed with Protein G crosslinked by antibody 8B8 which binds to αv integrin^96^. Bound αvβ8 is eluded from beads by washing the column with 100 mM glycine at pH 2.5. Flow through is immediately buffer adjusted by 2 M Tris-HCl pH 8, followed by size exclusion chromatography (Superose 6 Increase 10/300 GL, GE Healthcare) in 20 mM Tris-HCl pH 7.4, 150 mM NaCl, 1 mM CaCl_2_ and 1 mM MgCl_2_.

Full length of αvβ8 integrin was produced by transfecting ExpiCHO cells with integrin constructs using the manufacturer’s protocol. Cells were harvested after 3 days growth. Cells were solubilized by rotation in 4 °C using solubilize buffer for 3 hrs (20 mM HEPES, pH 8.0, 150 mM NaCl, 1 mM CaCl2, 1 mM MgCl2, 10 mM DDM, 2 mM CHS and 2% OG, 1x Protease Inhibitor Cocktail, EDTA-Free). Supernatant containing proteins were collected by centrifuged at 4,000 g followed by ultra-speed centrifuge at 45,000 rpm. Protein purification is carried out by affinity chromatography using a column packed with Protein G crosslinked by antibody C6D4F12 which binds to αvβ8 integrin. Bound full length αvβ8 is eluded from beads by washing the column with elution buffer (100 mM glycine at pH 2.5, 0.03% DDM). Flow through is immediately buffer adjusted by 2 M Tris-HCl pH 8.0, followed by size exclusion chromatography (Superose 6 Increase 10/300 GL, GE Healthcare) in 20 mM Tris-HCl pH 7.4, 150 mM NaCl, 0.03% DDM, 1 mM CaCl_2_ and 1 mM MgCl_2_. αvβ8 in nanodisc was made by adding at a ratio of αvβ8fl: MSP-2N2: lipid equals to 1: 4: 200 in 4 °C for 3 hrs, biobeads were added to remove the residue lipids over night by gentle rotation. αvβ8 in nanodisc was collected and further purified by size exclusion chromatography (Superose 6 Increase 10/300 GL, GE Healthcare) in 20 mM Tris-HCl pH 7.4, 150 mM NaCl, 1 mM CaCl_2_ and 1 mM MgCl_2_, the pooled and concentrated protein was subjected to SDS-PAGE, each protein size was identified to be corrected (Figure S4F).

Similarly, secreted L-TGF-β1/GARP was produced by transient transfecting Expi293 cells with three different constructs, L-TGF-β1 with R249A mutation, L-TGF-β1 with R249A and RGE mutation, and ectodomain of GARP with N-terminal Strep-His tag. This strategy favors formation of L-TGF-β1/GARP with a single intact RGD integrin binding motif. Cell culture was centrifuged to collect supernatant, which was filtered through a PES (polyether sulfone) membrane, 0.2 μm pore size (Millipore). Protein purification is carried out by using Ni-NTA agarose (QIAGEN), washed with three column volumes of 0.6 M NaCl, 0.01 M Tris (pH 8.0) and eluted with 0.25 M imidazole in Tris-buffered saline (TBS). The elution was then applied to Strep-tactin agarose (IBA) and washed with TBS (pH 7.4). To cleave the tag, 3.5 μl of commercial HRV-3C protease (Novagen, 1.8–3.0 U/μl) in TBS (pH 7.4) with 20% glycerol, was applied to the column, and incubated at 4°C overnight. The flow-through was washed with two column volumes of TBS (pH 7.4), then concentrated using centrifugal concentrators (Millipore) to about 1 mg/ml in 10 mM Tris (pH 7.4), 150 mM NaCl.

L-TGF-β3 was produced by transiently transfecting 293T cells with equal amounts of human L-TGF-β3 C4S_R277A_RGD and C4S_R277A_RGE plasmids.

C6D4F12 was produced by co-transfecting F12 VH pcDNA3.1 and F12 VL pcDNA3.1 into Expi*CHO* cells and antibody purified using protein G agarose, as described^22^.

The homogeneity and purity of all protein preparations were verified by SDS-PAGE stained with Coomassie blue and protein concentrations were measured by nanodrop.

#### Mass photometry

Mass photometry experiments were performed with a Refeyn OneMP (Refeyn Ltd.). Each sample in TBS buffer with 1 mM CaCl_2_, 1 mM MgCl_2_ of 16 μl was pipetted into the reaction chambers. Calibration was carried out by BSA, apoferritin and ADH. L-TGF-β1/GARP, L-TGF-β3/GARP, αvβ8, αvβ8/L-TGF-β1/GARP, and αvβ8/L-TGF-β3/GARP sample were diluted to 0.1 mg/ml, 1 μl of each sample was added to a 15 μl TBS with 1 mM CaCl_2_ and 1 mM MgCl_2_ buffer already pipetted into the reaction chamber. Image analysis was performed and analyzed by the software provided by Refeyn Ltd., with the default settings provided by the manufacturer.

#### Cryo-EM sample preparation

We co-expressed the recombinant GARP ectodomain, with L-TGF-β1 with a wild type integrin binding motif RGD (L-TGF(RGD)-β1) or a mutant form (L-TGF(RGE)-β1) that cannot bind to integrin. The resulting purified L-TGF-β1/GARP contains about 50% L-TGF-β(RGE/RGD)-β1, which can only bind one αvβ8 integrin, 25% L-TGF(RGD/RGD)-β1, which can bind two, and 25% L-TGF(RGE/RGE)-β1, which cannot bind to αvβ8. This design allows us to maximize the population of L-TGF-β/GARP bound with one αvβ8, reducing heterogeneity of the sample and facilitates particle alignment^22^. Such L-TGF-β1/GARP was mixed with αvβ8 in 1:1 molar ratio and incubated at room temperature for 30 min, the final protein complex concentration is 0.5 mg/ml. For cryo-EM grid preparation, 3 μl of the complex was deposited onto QUANTIFOIL^®^ R 1.2/1.3 on Au 300 mesh grids and UltrAuFoil^®^ R 1.2/1.3 on Au 300 mesh grids. Grids were pre-glow-discharged for 30 s at 15 mA prior to sample application and freezing. The complexes were frozen using a FEI Vitrobot Mark IV using a 1 s blot time with blot force 1. All grids were frozen with 100% humidity at 22 °C and plunge-frozen in liquid ethane cooled by liquid nitrogen.

L-TGF-β1/GARP-SpyCatcher was mixed with αvβ8 in 1:1 molar ratio and incubated at room temperature for 30 mins, the final protein complex concentration is 0.15 mg/ml. For cryo-EM grid preparation, 3 μl of the complex was deposited onto UltrAuFoil^®^ R 1.2/1.3 on Au 300 mesh grids, covered with graphene oxide functionalized by Spy-tag^97^, washed by 10 μl TBS buffer 3 times, finally 3 μl TBS buffer was added. The complexes were frozen using a FEI Vitrobot Mark IV using a 3 s blot time, with 100% humidity at 22 °C and plunge-frozen in liquid ethane cooled by liquid nitrogen.

L-TGF-β1/GARP mixed with αvβ8fl-nd in 1:1 molar ration and incubated at room temperature for 30 min, the final protein complex concentration is 0.5 mg/ml. For cryo-EM grid preparation, 3 μl of the complex was deposited onto QUANTIFOIL^®^ R 1.2/1.3 on Au 300 mesh grids. Grids were glow-discharged for 30 s at 15 mA prior to sample application and freezing. The complexes were frozen using a FEI Vitrobot Mark IV using a 1 s blot time. All grids were frozen with 100% humidity at 22 °C and plunge-frozen in liquid ethane cooled by liquid nitrogen.

To prepare the complex of L-TGF-β3/GARP with αvβ8, the molar ratio was 1:1, and incubated at room temperature for 30 min, the final protein complex concentration is 0.5 mg/ml. For cryo-EM grid preparation, 3 μl of the complex was deposited onto QUANTIFOIL^®^ R 1.2/1.3 on Au 300 mesh grids, grids were glow-discharged for 30 s at 15 mA prior to sample application and freezing. The complexes were frozen using a FEI Vitrobot Mark IV using a 1 s blot time. All grids were frozen with 100% humidity at 22°C and plunge-frozen in liquid ethane cooled by liquid nitrogen.

To prepare the complex of L-TGF-β1/GARP and L-TGF-β3/GARP, the concentration is 0.3 mg/ml. For cryo-EM grid preparation, 3 μl of the complex was deposited onto QUANTIFOIL^®^ R 1.2/1.3 on Au 300 mesh grids, grids were glow-discharged for 30 s at 15 mA prior to sample application and freezing. The complexes were frozen using a FEI Vitrobot Mark IV using a 1 s blot time. All grids were frozen with 100% humidity at 8°C and plunge-frozen in liquid ethane cooled by liquid nitrogen.

To prepare the complex of L-TGF-β3 with αvβ8, 100 μg of recombinant αvβ8 was incubated 150 μg L-TGF-β3, incubated at room temperature for 30 min, subjected to size exclusion chromatography and concentrated to 0.45 mg/ml. For cryo-EM grid preparation, 2.5 μl of the complex was deposited onto Quantifoil grids. 0.075 mg/ml sample was onto 400 mesh 1.2/1.3 copper Quantifoil Graphene-oxide grid, 0.05 mg/ml sample was onto 400 mesh 1.2/1.3 Au Quantifoil Graphene-oxide grid. 0.25 mg/ml sample was onto 400 mesh R 1.2/1.3 copper Quantifoil grid and 400 mesh R 1.2/1.3 Au Quantifoil grid. Except for the Graphene oxide grid, grids were glow-discharged for 60 s at 15 mA prior to sample application and freezing. The Graphene oxide grids were frozen using a FEI Vitrobot Mark IV using a 6 s blot time, the rest used 4 s. All grids were frozen with 100% humidity at 20°C and plunge-frozen in liquid ethane cooled by liquid nitrogen.

#### Cryo-EM data acquisition

All the automated data collections below were carried out using the SerialEM^87^. For L-TGF-β1/GARP, the data set was collected on a Thermo Fisher 300 KeV Titan Krios G2 equipped with a GATAN K3 direct detector camera. 2,939 movies were collected at a nominal magnification of 105,000x, the defocus range was set to be between −1.1 and −2.2 μm. The detector pixel size was 0.834 Å and the dose was 46 e^−^/Å^2^.

For αvβ8/L-TGF-β1/GARP and αvβ8/L-TGF-β1/GARP-SpyCatcher, the data set was collected on a Thermo Fisher 300 KeV Titan Krios G2 equipped with a GATAN K3 direct detector camera. 18,819 movies were collected at a nominal magnification of 105,000x, the defocus range was set to be between −0.8 and −2.5 μm. The detector pixel size was 0.835 Å and the dose was 68 e^−^/Å^2^. In the data set, 4176 movies were collected on QUANTIFOIL^®^ R 1.2/1.3 on Au 300 mesh grids, 7,777 movies were collected on UltrAuFoil^®^ R 1.2/1.3 on Au 300 mesh grids, 1,867 movies were collected on UltrAuFoil^®^ R 1.2/1.3 on Au 300 mesh grids by tilting 30°, 4,999 movies were collected on αvβ8/L-TGF-β1/GARP-SpyCatcher.

For αvβ8fl-nd, the data set was collected on a ThermoFisher 300 KeV Titan Krios G2 equipped with a GATAN K3 direct detector camera. 17,550 movies were collected at a nominal magnification of 105,000x, the defocus range was set to be between −0.8 and −2.5 μm. The detector pixel size was 0.8189 Å and the dose was 47.4 e^−^/Å^2^.

For L-TGF-β3/GARP, the data set was collected on a Thermo Fisher 300 KeV Titan Krios G2 equipped with a GATAN K3 direct detector camera. 3,666 movies were collected at a nominal magnification of 130,000x, the defocus range was set to be between −1.1 and −2.2 μm. The detector pixel size was 0.664 Å and the dose was 47 e^−^/Å^2^.

For αvβ8/L-TGF-β3/GARP, the data set was collected on a Thermo Fisher 200 KeV Talos Arctica equipped with a GATAN K3 direct detector camera. 1,654 movies were collected at a nominal magnification of 28,000x, the defocus range was set to be between −1.1 and −2.2 μm. The detector pixel size was 1.430 Å and the dose was 61 e^−^/Å^2^.

For αvβ8/L-TGF-β3, the datasets were acquired on a Thermo Fisher 300 KeV Titan Krios G2 operated in nano-probe mode at 300 kV equip- ped with a Gatan Quantum GIF energy filter operated in zero-loss mode with a slit width of 20 eV and a Gatan K2 Summit direct detector. Movies were recorded in super resolution mode with a super resolution pixel size of 0.6725 Å/pix and a nominal magnification of 105 kx at a dose rate of 7.7 e^−^/pix/s. Each 16 s movies contained 80 frames of 200 ms each, which corresponds to a total dose of ~70 e^−^/Å^2^, collected in a single session with a nominal defocus range of 1.2 – 2.4 μm under focus. 400 mesh 1.2/1.3 copper Quantifoil Graphene-oxide grid collected 878 movies, 400 mesh 1.2/1.3 Au Quantifoil Graphene-oxide grid collected 747 movies, 400 mesh R 1.2/1.3 copper Quantifoil grid collected 234 movies, 400 mesh R 1.2/1.3 Au Quantifoil grid collected 2,237 movies.

#### Imaging processing

For all individual datasets, dose fractionated super-resolution image stacks were motion corrected and binned 2 by Fourier cropping using MotionCor2^85^. The entire data processing and map reconstruction was carried out with cryoSPARC^88^. CTF estimation was performed by patch CTF module in cryoSPARC.

Cryo-EM dataset of αvβ8/L-TGF-β1/GARP-SpyCatcher with graphene oxide functionalized by Spy-tag grid was collected as a control experiment to rule out the influence of air-water interface on reconstruction. Reconstruction from this dataset show the same structural features as the one from αvβ8/L-TGF-β1/GARP complex. We therefore combined these two datasets together. All micrographs collected from αvβ8/L-TGF-β1/GARP and αvβ8/L-TGF-β1/GARP-SpyCatcher are combined. Initial particle picking carried out by multi template picking (L-TGF-β1/GARP with one or two integrins) identified 6,438,291 particles (Figures S3C–G). After multiple rounds of 2D classifications to eliminate duplicate particles and obvious junk, followed by Ab-Initio Reconstruction combined with Heterogeneous Refinement procedures in cryoSPARC, two major classes were identified, i.e., L-TGF-β1/GARP bound with two integrins (643,335 particles) and one integrin (1,324,888 particles). Further NU-refinement of the first class produced a reconstruction of L-TGF-β1/GARP bound with two integrins at a nominal resolution of 3.21Å. This map was further improved by DeepEmhancer^98^.

NU-refinement of the second class produced a reconstruction of L-TGF-β1/GARP bound with one integrin at a nominal resolution of 2.54Å. To resolve GARP, all particles in this class were subjected to two more rounds of Ab-Initio Reconstruction combined with Heterogeneous Refinement, which produced four classes. One class (94,847 particles) shows a relatively stable conformation of L-TGF-β1/GARP on top of αvβ8, with a minor density of a second αvβ8 binding. This class was subjected to further 3D classification (Beta), in cryoSPARC, in which, a sphere mask on GARP is applied. This procedure isolated 45,913 particles with GARP in an up conformation, 30,190 particles with GARP in a down conformation, with the remaining ambiguous. Particles of both classes were further classified by an additional round of Ab-Initio Reconstruction combined with heterogeneous refinement to remove remaining particles bound with two αvβ8. For the class of GARP facing down and one integrin bound (15,748 particles), further refinement by NU-refinement produced a 4.12Å resolution reconstruction. For the class of GARP facing up and one integrin bound (22,815 particles), one more round of Ab-Initio reconstruction combined with heterogeneous refinement produced a reconstruction of αvβ8/L-TGF-β1/GARP with GARP facing up at 4.05 Å. For particles with GARP in the down conformation, a similar procedure was applied to produce a final reconstruction of αvβ8/L-TGF-β1/GARP at 4.11Å resolution.

All discarded particles during the above processes were pooled and subjected to multiple rounds of Ab-Initio Reconstruction combined with Heterogeneous Refinement, to rescue all particles that belong to the GARP in up conformation with one αvβ8 bound. Merging the rescued particles with the existing particles from well-resolved classes produced a total of 46,771 particles, that were used for a final round NU-refinement, producing a final reconstruction of L-TGF-β1 with GARP in the up position and bound with one αvβ8 at a nominal resolution of 3.91Å. Further local refinement with masks on either L-TGF-β1/GARP or αvβ8 produced two final reconstructions with nominal resolutions of 3.3Å, and 3.1Å, respectively. Because two masks overlap with each other, these two reconstructions were merged by joining the common region into a final combined map. A focused 3DVA was performed on this final particle stack by applying a mask on L-TGF-β1/GARP to reveal the motion of this part of the reconstruction. By random splitting this 46,771 particles data to 3 parts, each part was performed NU-refinement, producing a final reconstruction. Further local refinement with masks on either L-TGF-β1/GARP or αvβ8 produced two final reconstructions. Local resolutions were determined and compare with the full data set of 46,771 particles.

The class with 1,324,888 particles of L-TGF-β1/GARP with one αvβ8 bound underwent further guided classification by multiple rounds of Ab-Initio Reconstruction combined with Heterogeneous Refinement, using a map of L-TGF-β1/GARP as a reference. This isolates 34,117 particles of L-TGF-β1/GARP alone not bound with αvβ8. NU-refinement result of this subset produced a reconstruction of 3.47Å, to which we applied DeepEmhancer procedure to improve structural features.

All particles that produced the two clear reconstructions described above as well as the reconstruction of L-TGF-β1/GARP alone were removed from the1,324,888 particles dataset, and remaining particles were transferred to Relion 3.8^99^ for focused classification without alignment with a sphere mask applied on L-TGF-β1/GARP. Each good class was transferred back to cryoSPARC for NU-refinement. Among them is a class that is αvβ8 alone, at a resolution of 4.54 Å. Particles of all good classes with one αvβ8 were pooled and subjected to a 3DVA masked on L-TGF-β1/GARP and partially on αvβ8.

The data processing of L-TGF-β1/GARP is shown in Figure S3H. The entire data processing and map reconstruction was carried out with cryoSPARC. The initial particle picking identified 1,845,468 particles. After several rounds of 2D classifications, Ab-Initio Reconstruction combined with Heterogeneous Refinement, about 318,954 particles were isolated for NU-refinement, yielding a map with a nominal resolution of 3.0Å. By random splitting this data to 3 parts, each part was performed NU-refinement, producing a final reconstruction. Local resolutions were determined and compare with the full data.

The data processing of αvβ8/L-TGF-β1/GARP/MHG8-Fab is shown in Figure S4E. The entire data processing and map reconstruction was carried out with cryoSPARC. The initial particle picking identified 292,348 particles. After several rounds of 2D classifications, Ab-Initio Reconstruction combined with Heterogeneous Refinement, about 16,774 particles were isolated for NU-refinement, yielding a map with a nominal resolution of 7.72Å. Further classifications were applied, showing not much density could be resolved well on αvβ8.

Data processing of αvβ8fl-nd/L-TGF-β1/GARP is shown in Figure S4G. The entire data processing and map reconstruction was carried out with cryoSPARC. The initial particles picking identified 2,335,916 particles. After multi rounds of 2D classifications, Ab-Initio Reconstruction combined with Heterogeneous Refinement, about 20,547 particles were isolated for NU-refinement, yielding a map with a nominal resolution of 3.45Å. Further local refinement with masks on either L-TGF-β1/GARP or αvβ8 produced two final reconstructions with nominal resolutions of 6.3Å, and 3.1Å, respectively. Because two masks overlap with each other, these two reconstructions were merged by joining the common region into a final combined map.

The data processing of L-TGF-β3/GARP is shown in Figure S5A. The entire data processing and map reconstruction was carried out with cryoSPARC. The initial particle picking identified 522,776 particles. After several rounds of 2D classification followed by Ab-Initio Reconstruction combined with Heterogeneous Refinement, about 151,804 particles were isolated for NU-refinement, yielding a nominal resolution of 2.93Å.

The data processing of αvβ8/L-TGF-β3/GARP is shown in Figure S5E. The entire data processing and map reconstruction was carried out with cryoSPARC. The initial particle picking identified 1,007,086 particles. After several rounds of 2D classification, Ab-Initio Reconstruction combined with Heterogeneous Refinement yielded about 151,804 suitable particles which were used to separate into 3 classes, with L-TGF-β3/GARP in distinct conformations, and NU-refinement was performed for each class, yielding nominal resolutions of 5.89Å, 4.86Å and 7.19Å.

Data processing of αvβ8/L-TGF-β3 is shown in Figure S5F. The entire data processing and map reconstruction was carried out with cryoSPARC. The initial particle picking identified 779,392 particles. After several rounds of 2D classifications, Ab-Initio Reconstruction combined with Heterogeneous Refinement, about 382,107 particles were isolated and used for NU-refinement, yielding a nominal resolution of 2.73Å. In the intermediate data set, 537,399 particles were identified and extracted to Relion 3.8, masked on L-TGF-β3 for 3D classification without alignment, and each class transferred back to cryoSPARC for NU-refinement.

#### Model building and refinement

For atomic model building of αvβ8/L-TGF-β1/GARP, the atomic models of αvβ8 (PDB: 6UJA)^22^ and L-TGF-β1/GARP/MHG8 crystal structure (PDB: 6GFF)^9^ were used as an initial model. For atomic model building of L-TGF-β1/GARP, atomic model of αvβ8/L-TGF-β1/GARP with αvβ8 portion removed was used as an initial model. For atomic model building of L-TGF-β3/GARP, a homology model predicted by Swiss-model^100^ based on crystal structure (PDB: 6GFF) was used as the initial model. For atomic model building of αvβ8/L-TGF-β3, the atomic models of αvβ8 (PDB: 6UJA)^22^ and crystal structure (PDB: 4UM9)^101^ were used as initial models.

In each case, the initial model was fitted as a rigid body into corresponding cryo-EM map by UCSF Chimera followed by Phenix real_space_refinement^102^. ISOLDE^103^ in UCSF ChimeraX^100^ was used to build disulfide and to refine the side chain. The model was checked in COOT^104^with some side chains manually adjusted to fit the density, followed by a final real space refinement in Phenix with a B-factor refined for each residue.

#### B-factor comparison

For L-TGF-β1/GARP, αvβ8tr/L-TGF-β1/GARP, and L-TGF-β3/GARP, each atomic model, B-factors of individual residues were refined during the final real space refinement in Phenix^102^. In all structures, except αvβ8fl-nd, L180 in GARP is relative stable and has the relative lower B-factor, thus was chosen as a common reference point with its B-factor set to 0Å^2^. The B-factor of every other residue in the structure was normalized by the difference of its refined B-factor with that of L180.

For αvβ8tr/L-TGF-β1/GARP and αvβ8fl-nd, some relative stable resides (L106, L171, V228, A281, Y374, M408) in αv subunit were averaged in each model, and chosen to be normalized by division between two models.

The difference of B-factor of individual residue between two structures was measure by the difference of the normalized B-factor. B-factor coloring was created by UCSF ChimeraX^105^. Because of the sequence differences between L-TGF-β1 and L-TGF-β3, the B-factor differences between the two were measured by the difference of the normalized B-factor assigned to Cα atoms. B-factor Cα coloring was created by UCSF ChimeraX^105^.

#### RMSD measurement

The Root Mean Square Deviation (RMSD) between L-TGF-β1/GARP and L-TGF-β1/GARP/αvβ8 was measure by the command *rmsd sel* in Chimera, only the shared resolved residues were selected for each subunit. RMSD between GARP is 1.336 Å, RMSD between non-integrin binding subunit of L-TGF-β1 is 1.934 Å, RMSD between integrin binding subunit of L-TGF-β1 is 2.034Å.

#### Molecular dynamics simulations

The cryo-EM structure of the L-TGF-β1/GARP complex was used as a reference starting structure for the molecular dynamic simulations. The unresolved loops were modeled using Rosetta protocols^106^. The GARP β-hairpin loop, I271 to A280, was added based on homology modeling with the X-ray crystal structure (PDB ID: 6GFF)^9^. The CHARMM^107^ 36m force field was used for the protein and ions. The CHARMM-modified TIP3P forcefield^108^ was used for the water molecules. The L-TGF-β1/GARP complex was inserted in a box of dimensions 150.4 Å × 150.7 Å × 150.9 Å and solvated with 99,086 water molecules. This arrangement ensured that each protein atom was at least 20 Å away from its periodic image. To neutralize the charge of the entire system, 284 Na+ and 281 Cl− ions were added. The final system comprised a total of 318,045 atoms. The molecular dynamics simulations were performed with GROMACS 2023^109^. We employed periodic boundary conditions and calculated nonbonding interactions using the particle mesh Ewald method^110^with a 12Å cutoff. The bonds involving hydrogen atoms were restrained using the LINCS algorithm^111^. We used the Nose-Hoover thermostat and Parrinello-Rahman Barostat. First, an NVT equilibration for 125 ps at 303 K was performed with a friction coefficient of 1 ps^−1^and timestep of 1fs. Then, the timestep was set to 2 fs and 10 ns of an NPT equilibration was performed until the average pressure of the system was equilibrated to 1 atm at 303 K. The final frame of the NPT equilibration was extracted to initiate two production runs, each 1 μs long. Conformational snapshots were saved every 200 ps. We used MDAnalysis^107^to extract the root-mean-square-fluctuation (RMSF) by aligning to the first frame of the production run and using only Cɑ atoms. The average RMSF is calculated between the two replicas.

#### Sequence alignments

Multiple protein sequence alignments for L-TGF-β were generated using Clustal Omega^93^, and 2dSS (http://genome.lcqb.upmc.fr/2dss/contact.html).

#### Retroviral production and transduction

β8 retroviral particles were assembled in the Phoenix 293 amphotropic cell line via transfection of pBabe β8 using lipofectamine 3000 reagent (Invitrogen) per the manufacturer’s instructions. To maximize transfection efficiency phoenix cells were plated onto Poly-L-Lysine (Sigma Aldrich) coated 6-well culture plates at a density of 3.5×10^5^ cells/ml 1 day before transfection. Media was replaced 3 hrs prior to transfection, and retroviral supes were harvested 48 hrs after transfection. MFB-F11 cells were then transduced with retroviral supernatants by incubating 1×10^5^ MFB-F11 cells with 100 μL of retroviral supernatant for 5 mins in suspension before plating out onto 24 well cell culture plates containing 500 μL basal media. Puromycin resistant colonies were then selected by supplementation of basal media with 5 μg/mL puromycin dihydrochloride (Sigma-Aldrich, #P8833). Cells were screened for the presence of cell surface β8 by flow cytometry.

#### Flow cytometry and immune cell analysis

To normalize expression levels of cell surface L-TGF-β proteins, TMLC cell lines were stained with anti-HA (clone 5E11D8) and green fluorescent protein (GFP) expression as a surrogate marker for L-TGF-β expression. Cells were stained in MACS staining buffer on ice for 15 min, before washing and staining with anti-mouse-APC conjugated secondary antibody (Biolegend # 405308). Cells were then sorted for uniform cell surface expression. To confirm the expression of human β8, MFB-F11 cells were stained with anti-β8 antibody (Clone F12) for 15 mins on ice at a concentration (1 μg/ml). Cells were then washed and subsequently stained with anti-mouse-APC conjugated secondary antibody (Biolegend #405308).

To measure Treg frequency in mice, peripheral blood and splenocyte cell preparations were collected. Briefly, whole blood was collected into EDTA-containing tubes, centrifuged at 700xg for 20 minutes and the subsequent cell pellet lysed with ACK lysis buffer for 10 min and washed twice with >5x volumes of staining buffer (PBS + 2.0% BSA + 0.5 mM EDTA). Splenocytes were manually dissociated, passed through a 70 μm filter, and red blood cells lysed in ACK buffer for 5 min before washing and resuspending in staining buffer. Cells were stained with viability dye for 20 minutes at room temperature followed by blocking Fc receptors with anti-CD16/CD32 for 10 min at 4°C. Cells were then stained with an antibody cocktail diluted in Brilliant Staining Buffer (BD biosciences) with anti-mouse surface antibodies (see Key Resources Table) for 15 min at 4°C. Cells were fixed and permeabilized using the FoxP3/Transcription Factor Fixation/Permeabilization kit (eBioscience) for 45 min at 4°C. Cells were stained overnight with intracellular antibodies, washed and ultimately resuspended in staining buffer before acquiring data the next day on an Aurora spectral flow cytometer (Cytek) with SpectroFlo (Cytek) software. Data was analyzed using SpectroFlow (CyTek) for pre-processing of spillover matrices and FlowJo (BD biosciences) for population gating. CD4+ Treg were defined by FSC × SSC profile (lymphocyte gate), single cells, live, CD45+, Ly6g−, CD90+, CD19−, TCR_β_+, CD4+, CD8a−, CD25+, FoxP3+.

#### Assays to measure TGF-β1 activation by different forms of αvβ8.

TMLC reporter cells expressing GARP/L-TGF-β1 were cultured in the presence of plate-bound (immobilized) _α_v_β_8 ectodomain, soluble αvβ8 ectodomain, soluble αvβ8fl-nd or empty (Control) nanodisc. All integrins were plated using a 2-fold dilution series ranging from 10 μg/mL-0.039 μg/mL). αvβ8 ectodomain or BSA control were incubated on 96 well culture plates in the presence of PBS containing 1 mM MgCl_2_ and 1 mM CaCl_2_ for 1 hr at 37 °C. Wells were then washed 2x in basal media before αvβ8 coated wells and all other assay wells were blocked for 1 hr in basal media at 37 °C. After blocking, soluble αvβ8 ectodomain, αvβ8fl-nd or empty nanodisc were plated in basal media using the same 2-fold dilution series described above. TMLC cells were then plated at an equal density (1×105 cells/mL in basal media) in triplicate in 96-well cell culture plates (Corning, # 3599). After attachment cells were incubated for 16 hours under standard cell culture conditions (37 °C humidified incubator, 5% CO_2_). After 16 hrs cells were lysed and assessed for luciferase activity as previously described^27,95^.

#### Assays to measure TGF-β3 activation

The αvβ8 ectodomain was coated along with the negative control, BSA (Sigma-Aldrich) (1 μg/ml) onto 96 well cell culture plates (Corning) in PBS (1mM Ca^2+^ and 1mM Mg^2+^) for 1 hour at RT as described^22^. Wells were then washed 1x in PBS and blocked in 1% BSA in PBS for 1 hr at RT. TMLC L-TGF-β1/GARP, TMLC L-TGF-β3/GARP or TMLC L-TGF-β1_lasso3_chimera/GARP were then plated onto coated wells at density of 1×10^5^ cells/mL. After attachment cells were incubated for 16 hours under standard cell culture conditions (37 °C humidified incubator, 5% CO_2_). To measure diffusible TGF-β, media was removed from culture wells and plated onto fresh wells containing 1.5×10^4^ TMLC WT cells/mL for a further 16 hrs before lysis in luciferase assay lysis buffer and measurement of luciferase activity as described above. To measure cell intrinsic flexibility of L-TGF-β3, TMLC L-TGF-β1/ GARP, TMLC L-TGF-β3/GARP or TMLC L-TGF-β1_lasso3_chimera/GARP cells were lysed and assayed for luciferase activity as above.

#### Assays to measure the signaling direction of the αvβ8/L-TGF-β1/GARP complex

MFB-F11 TGF- β reporter cells expressing human β8 were co-cultured with TMLC TGF-β reporter cells expressing cell surface L-TGF-β1 via the adaptor molecule GARP. Cells were plated at an equal density (1×10^5^ cells/mL in basal media) in triplicate in 96-well cell culture plates (Corning, # 3599). To quantify TGF-β signaling, TMLC or MFB-F11 cells were also plated alone at a density of 1×10^5^ cells/mL in basal media supplemented with known concentrations of rhTGF-β1 (R&D systems, #240-B-002/CF). After attachment cells were incubated for 16 hours under standard cell culture conditions (37°C humidified incubator, 5% CO_2_). After 16 hours conditioned media was removed for assessment of SEAP activity. Attached cells were washed once in PBS and subsequently lysed in an equal volume of luciferase assay lysis buffer for 30 mins at room temperature to detect luciferase activity (Biotium, # 26140-079). Measurement of SEAP activity was achieved using the SEAP reporter gene assay kit (Abcam, #133077). Briefly, conditioned media was heated at 65 °C to denature endogenous alkaline phosphatases. Conditioned media samples were then transferred to 96-well cell culture white plates (Costar, # 3917) and an equal volume of SEAP substrate was added to each well. Samples were incubated for 5 min before Luminescence was recorded using a luminescence assay plate reader (Promega: Glomax explorer). Measurement of luciferase activity was performed using a luciferase assay kit as described above. SEAP or luciferase activity was converted to ng/ml TGF-β signaling by interpolation from the standard curve of known rhTGF-β1 treatments. These assays were repeated with the following cell lines: TMLC L-TGF-β1 (RGD)/GARP, TMLC L-TGF-β1 (RGE)/GARP and TMLC L-TGF-β3/GARP.

#### TGFβR2 binding assay

ELISA plates were coated with 10 μg/ml recombinant αvβ8 in 20 mM HEPES pH 8.0, 150 mM NaCl (HBS)with 1mM CaCl_2_ and 1 mM MgCl_2_ for 1 hr at RT. Wells were then washed in HBST (0.5% Tween-20) and blocked in HBS with 2.5% BSA for 1 hr at RT. Wells were then washed in HBS-T (0.5% Tween-20) and applied by 8 μg/ml TGF- β1/GARP, 4 μg/ml TGF-β3/GARP or in HBS with 1 mM CaCl_2_ and 1 mM MgCl_2_, after 5 minutes incubation at RT, serial dilutions of recombinant TGF-βR2-Fc in HBS with 1 mM CaCl_2_ and 1 mM MgCl_2_ were added for 1 hr at RT. Wells were then washed in HBST (0.5% Tween-20) and antibody were added (2 μg/ml) in HBS with 1 mM CaCl_2_ and 1 mM MgCl_2_ for 1 hr at RT. After washing in in HBST (0.5% Tween-20), bound antibodies were detected using anti-mouse-HRP using TMB substrate and colorimetric detection (Glomax Explorer, Promega).

#### iTreg conversion assay

CD4+ T cells isolated from whole mouse spleen using a CD4+ T cell isolation kit (Miltenyi, Cat:130-104-454 as previously described^27^). Purified CD4+ T-cells were then cultured in Serum Free Medium, containing IMDM (Gibco, Cat:12440-053), 1% Insulin-Transferrin-Selenium (Gibco, Cat:41400-045), 50 uM 2-Mercaptoethanol (Gibco, Cat:31350-010), 2mM Retinoic acid (Sigma, Cat:R2625), and 135 U/ml IL-2 (R&D, Cat:402-ML/CF) for 72 h in 24-well plates with at a density of 1.0 × 10^6^/ml. Cells were plated onto wells coated with 6 μg/mL anti-Mouse-CD3 (Biolegend, Cat:10020) with 12 μg/mL recombinant αvβ8 ectodomain or BSA control. Recombinant human TGF-b1 (1 ng/mL, R&D, Cat:240-B) was also used as a positive control. After 72 h, the cells were immunostained with anti-mouse-CD25-APC (Biolegend, Cat:102012) and anti-mouse-Foxp3-PE (Biolegend, Cat:126404) and detected by flow cytometry.

#### Immunoblots

Cells were lysed with RIPA buffer (Sigma, Cat:R0278) supplemented with protease inhibitor cocktail (Thermo Scientific^™^, Cat:87786) and phosphatase inhibitor (Thermo Scientific^™^, Cat:A32957). Protein concentration was determined by BCA assay (Thermo Scientific^™^, Cat:23228). The precast gels (Cat:456-1084) and transfer pack (Cat:1704158) were bought from Bio-Rad. SDS-PAGE and WB were performed according to the manufacturer’s instructions. Anti-TGF-β1 (Cat:ab179695), anti-p-Smad 2/3 (CST, Cat:8828S), anti-Smad 2/3 (CST, Cat:3102S), anti-Na.K-ATPase (Invitorgen, Cat:MA5-32184), and anti-Actin (Sigma, Cat:A2228-200ul) were all used with a dilution factor of 1:1000. anti-Mouse-IgG (Jackson Immunoresearch, cat:711-035-152) and anti-Rabbit-IgG (Jackson Immunoresearch, cat:715-035-150) were used with a dilution factor of 1:5000. When assessing protein expression of TGF-β1 in mouse plasma, it was necessary to pre-clear endogenous IgGs using 3 sequential overnight Sepharose G incubations at 4 C. The supernatant was then used for WB detection of TGF-β1expression levels in plasma.

#### Cell surface biotinylation

WT CD4+ T cells were harvested after activation, as above, from culture wells by gentle pipetting. After 3 washes with cold PBS, cells were resuspended with ice-cold PBS at a density of 2.5 ×10^**7**^/ml. NHS-LC-Biotin was (Thermo Scientific^™^, cat: A39257) was used following the manufacturer’s instructions. WT CD4+ T-cells were lysed before or after surface biotinylation. Lysate from non-biotinylated control cells were compared to eluates from equal amounts of biotinylated and non-biotinylated lysates applied to streptavidin agarose (SA), with anti-mature TGF-β1. A band the same size of cleaved mature TGF-β1 was seen in eluates from SA beads incubated with non-biotinylated lysate, making quantitative comparisons of cleaved to uncleaved forms of TGF-β1 difficult. Membranes were stripped and reprobed with anti-Na+/K+ ATPase, as a cell membrane marker.

#### Quantification and statistical analysis

Assays are reported as means ± s.e.m., (≥3). Statistical analyses were performed using Prism 9 (GraphPad Software, San Diego, CA).

## Supplementary Material

1 **Figure S1. Domain organization of L-TGF-β1 and -β3, related to Introduction,** Figure 2 and Figure 4. Sequence alignment with secondary structure prediction of L-TGF-β1 and L-TGF-β3. Domains are colored as in (Figure 2A**)** and secondary structure prediction marked. Sequence numbering does not include signal peptide and is consistent in all figures and text.

2 **Figure S2. Engineering of *tgfb1 R278A* KI/KI mice and immune characterization of *tgfb1* −/− mice, related to** Figure 1. (**A**) From top to bottom, schematic of the *tgfb1* targeted locus, targeting construct after germline integration, after crossing to mice expressing flippase (flp), after crossing to rosa 26-cre. (**B**) Scatter plots showing Treg from PBMC isolated from post-natal day 18 *tgfb1 −/−* (KO/KO) mice compared with day 18 littermate controls (WT/KO). Staining done as in Figure 1. (**C**) Scatter bar graphs showing Treg as a percentage of CD4+ T-cells (n=3 KO/KO; n=3 WT/KO). Shown is SE. *** p<0.001.

3 **Figure S3. Single particle cryo-EM and image processing of L-TGF-β1/GARP and αvβ8/L-TGF-β1/GARP complex, related to** Figure 2. **(A)** Mass photometry analysis of L-TGF-β1/GARP mixed with ectodomain of αvβ8. Three peaks correspond to L-TGF-β1/GARP or αvβ8 alone at ~180kD, one L-TGF-β1/GARP bound with one αvβ8 at ~380kD, and one L-TGF-β1/GARP bound with two αvβ8 integrins at ~560kD.
(**B**) Co-culture of MFB-F11 αvβ8 expressing cells with TMLC L-TGF-β1/GARP presenting cells is sufficient to activate the TGF-β signaling pathway by TMLC. To test whether one RGD site is sufficient for αvβ8-mediated TGF-β activation, TMLC-L- TGF-β1/GARP cells were made using a 1:1 transfection ratio of TGF- β1(RGD) and TGF- β1(RGE) (pink squares). TMLC: L- TGF-β1(RGE/RGE)/GARP cells are also included to demonstrate the requirement of RGD for αvβ8/L-TGF- β1 binding and activation (green triangles). The results (vertical axis) are normalized against a standard TGF-β activation curve. **p<0.01 by one-way ANOVA followed by Sidak’s multiple comparison test for the indicated comparisons
**(C)** Representative electron micrograph of frozen hydrated sample of mixing αvβ8 with L-TGF-β1/GARP in 1:1 molar ratio.
**(D)** Representative 2D class averages calculated from particles selected for further processing.
**(E)** Flow-chart of cryo-EM data processing. FSC curves for resolution estimations are included at various steps of the flow-chart.
**(F)** Motions captured in αvβ8/L-TGF-β1/GARP complex along five eigenvectors from 3DVA are illustrated.
**(G)** 3DVA of particles in Class 1 in Figure 2F.
**(H)** Image processing flow-chart from representative electron micrograph to the final reconstruction and FSC for final resolution estimation of L-TGF-β1/GARP.

4 **Figure S4. Single particle cryo-EM, molecular dynamics of L-TGF-β1/GARP, and image processing of αvβ8/L-TGF-β1/GARP bound with Fab MHG8, related to** Figure 3. **(A)** The B-factor from cryo-EM analysis (red) and the average RMSF (black) from long molecular dynamics simulations are shown for each residue of L-TGF-β1/GARP where flexible residues correlate with high B factors.
**(B)** The local sharpen maps (colored) superimposed in related low passed 8 Å maps (Grey in transparent) of class 3–8 from Figure 2F. **(C)** B factor analysis of models for (B)
**(D)** The weighted averaged B factor of the models in (B) (Left) compared to apo L-TGF-β1/GARP (Right).
**(E)** Image processing flow-chart from representative electron micrograph to the final reconstruction and FSC for final resolution estimation of αvβ8/L-TGF-β1/GARP bound with Fab MHG8.
**(F)** SDS-PAGE of αvβ8fl-nd.
**(G)** Image processing flow-chart from representative electron micrograph to the final reconstruction and FSC for final resolution estimation of αvβ8fl-nd/L-TGF-β1/GARP.
**(H)** The binding affinity assay of L-TGF-β1/GARP with αvβ8tr, αvβ8tr-clasped and αvβ8fl-nd showing no difference.

5 **Figure S5. Single particle cryo-EM and image processing of L-TGF-β3/GARP, αvβ8/L-TGF-β3/GARP complex and comparison of binding interface between GARP and L-TGF-β, related to** Figure 4. **(A)** Image processing flow-chart from representative electron micrograph to the final reconstruction and FSC for final resolution estimation for L-TGF-β3/GARP.
**(B)** Three regions of cryo-EM density map and docked atomic model selected from the interface between L-TGF-β1 (magenta) and GARP (yellow).
**(C)** Three regions of cryo-EM density map and docked atomic model selected from the interface between L-TGF-β3 (green) and GARP (yellow).
**(D)** Overlay of the atomic models of L-TGF-β1/GARP (magenta/yellow) and L-TGF-β3/GARP (green/yellow) from the same selected regions.
**(E)** Flow-chart of processing single particle cryo-EM dataset of αvβ8/L-TGF-β3/GARP complex.
**(F)** Flow-chart of processing single particle cryo-EM dataset of αvβ8/L-TGF-β3 complex.

6 Figure S6. Representative flow cytometry scatter plots demonstrating the percentage of cell surface L-TGF- β1, L-TGF-β3 and L-TGF- β 1(lasso3) respectively, related to Figure 5.

7 **Figure S7. B-factor for each pro-TGF-β superfamily crystal structure, related to Discussion and** Figure 5. Ribbon diagram of BMP9 (PDB: 4YCG), BMP10 (PDB: 7POI), and Activin A (PDB: 5HLY). Residues are colored by B-factors. Residues with lower B-factor (blue) are relatively stable, while those with higher B-factor (red) are more flexible. An enlarged view within dashed box for each show that the area around TGF-βR binding site, in which each lasso loop (dashed oval) is flexible.

8 **Figure S8. Raw immunoblots, related to** Figure 1. (**A**) Raw immunoblot of plasma data depicted in Figure 1M. Plasma was subject to 3 sequential rounds of IgG pre-clearance using Sepharose G beads. Lanes 9 (WT/WT), 10 (KO/KO) and 11(KI/KI) are depicted in Figure 1M. Samples in Lanes 1,2 and 3, and 5,6 and 7 represent the samples after 1 or 2 rounds of pre-clearing respectively. (**B**) Raw immunoblot of TGF-β1 expression in murine organ homogenates (Figure 1M). Lanes 1,2 and 3 show TGF-β1 staining from WT/WT, KO/KO and KI/KI mice respectively in kidney, Lanes 4, 5 and 6 in liver, Lanes 7, 8 and 9 in lung, and 10, 11 and 12 in spleen. (**C**) Raw immunoblot representing the β-actin expression depicted in figure 1M as a loading control. (**D**) Raw immunoblot data for Figure 1N demonstrating the expression of uncleaved TGF-β1 (upper blot) after 0.9 seconds exposure, and cleaved TGF-β1 (lower blot) after 14.9 seconds exposure. Included below the TGF-β1 blots is a raw immunoblot stained with anti-β-actin to ensure equal protein loading. (**E**) raw immunoblot of anti-pSMAD2/3 (upper blot) and anti-SMAD2/3 (lower blot) presented in Figure 1P. Included below the SMAD2/3 blot is an immunoblot using anti-β-actin to ensure equal protein loading. (**F**) Raw immunoblots for data depicted in Figure 1O. The upper blot displays anti-TGF-β1 staining. Lane 1 (non-biotinylated lysate), Lane 2 (non-biotinylated lysate after Sepharose pre-clearance), Lane 3 (eluate from pre-cleared lysate incubated with streptavidin beads), Lane 4 (blank), Lane 5 (biotinylated lysate) Lane 6 (biotinylated lysate after Sepharose pre-clearance), Lane 7 (eluate from pre-cleared biotinylated lysate incubated with streptavidin beads). The lower image depicts the raw immunoblot data depicting anti-Na+/K+ ATPase staining to demonstrate cell surface protein enrichment. Figure 1O depicts Lane 5, lane 7 and Lane 3 respectively.

9 **Movie S1. *tgfb1***^***R278A/R27A***^ **mice are indistinguishable from WT, related to** Figure 1. 30 second movie comparing the movement and grooming of homozygous *tgfb1*^*R278A/R27A*^ mice (right) compared to littermate controls (left). At the time of recording mice were 146–154 days old. A scale bar is included in the movie.

10 **Movie S2**: **Movie of motions revealed by 3D variation analysis, related to** Figure 2 and 3. Movie shows motion revealed by 3D variation analysis (3DVA) on particles of Class 1 of αvβ8/L-TGF-β1/GARP complex along the first eigenvector.

11

## Figures and Tables

**Table:** KEY RESOURCES TABLE

REAGENT or RESOURCE	SOURCE	IDENTIFIER
Antibodies		
Anti-mouse TGF-β1	Abcam	ab179695
Anti-human, mouse αvβ8, C6D4	Takasaka et al., 2018^80^	N/A
Anti-human αv, 8B8	Mu et al., 2002^18^	N/A
Anti-mouse HRP	GE Healthcare	Cat. # NA931V; RRID:AB_772210
Anti-LAP-β1-biotin or APC conjugated	R&D Systems	Cat. # BAF246; RRID:AB_356332
Anti-LAP-β1	R&D Systems	Cat. # AF426; RRID:AB_354419
Anti-GARP	BioLegend	Cat. # 352502
Anti-human, mouse αvβ8	This paper	C6D4F12
anti-mouse-APC	Biolegend	Cat. # 405308
anti-HA (clone 5E11D8)	Thermo Fisher	Cat. # A01244-100
Anti-Na+/K+ ATPase	Invitrogen	Cat#MA5-32184
Anti-Actin	Sigma	Cat#A2228
Anti-pSMAD2/3	Cell Signaling	Cat#8828S
Anti-SMAD2/3	Cell Signaling	Cat#3102S
Live Dead Fixable Blue	Thermo	Cat#L23105
ACK lysis buffer	Thermo	Cat#A1049201
Fc receptor block (CD16/32)	BD biosciences	Cat# 553141
Brilliant Staining Buffer	BD biosciences	Cat# 563794
Foxp3 / Transcription Factor Staining Buffer Set	eBioscience	Cat#00-5523-00
Anti-mouse CD3	Biolegend	Cat#10020
Anti-mouse CD45 (clone 30-F11)	Biolegend	Cat. # 103128
Anti-mouse CD45 (clone 30-F11)	Biolegend	Cat. # 103128
Anti-CD90.2 (clone 53-2.1)	BD biosciences	Cat. # 565257
Anti-mouse CD19 (clone 6D5)	Biolegend	Cat. # 115566
Anti-mouse TCRβ (clone H57-597)	Biolegend	Cat. # 109249
Anti-mouse CD4 (clone RM4-5)	Biolegend	Cat. # 100559
Anti-mouse CD8a (clone 53-6.7)	Biolegend	Cat. # 100740
Anti-mouse CD25 (clone PC61)	Biolegend	Cat. # 102072
Anti-mouse FoxP3 (clone MF-14)	Biolegend	Cat. # 126405
CD4+ T cell isolation kit	Miltenyi	Cat#130-104-454
Serum Free Medium, containing IMDM	Gibco	Cat#12440-053
1% Insulin-Transferrin-Selenium	Gibco	Cat#41400-045
2-Mercaptoethanol	Gibco	Cat#31350-010
Retinoic acid	Sigma	Cat#R2625
IL-2	R&D Systems	Cat#402-ML/CF
Recombinant TGF-β1, human	R&D Systems	Cat#240-B
Anti-mouse CD25-APC	Biolegend	Cat#102012
anti-mouse Foxp3-PE	Biolegend	Cat#126404
RIPA buffer	Sigma	Cat#R0278
Protease inhibitor cocktail	Thermo Scientific	Cat#87786
Phosphatase inhibitor	Thermo Scientific	Cat#A32957
BCA assay	Thermo Scientific	Cat#23228
Anti-Mouse-IgG	Jackson Immunoresearch	Cat#711-035-152
Anti-Rabbit-IgG	Jackson Immunoresearch	Cat#715-035-150
Bacterial and virus strains		
DH5a Chemically Competent E. coli	Thermo Fisher	Cat. # 18265017
Biological samples		
None		
Chemicals, peptides, and recombinant proteins		
Puromycin	Sigma Aldrich	Cat. #P8833
Hygromycin	Thermo Fisher	Cat. # 10687010
G418 sulfate	Thermo Fisher	Cat. # 10131035
HRV-3C protease	Millipore Sigma	Cat. # 71493-3
Gibson Assembly Cloning Kit	NEB	Cat. # #E5510S
KAPA Mouse Genotyping Kit	Sigma Aldrich	KR0385_S – v3.20
Protein-G Agarose	Pierce	Cat. # 20398
Strep-tactin agarose	IBA	Cat. # 2-1204-001
Ni-NTA agarose	Qiagen	Cat. # 30210
Lipofectamine 3000	Thermo Fisher	Cat. # L3000001
rhTGF-β1	R&D systems	Cat. # #240-B-002/CF
NHS-LC Biotin	Thermo	Cat#A39257
Critical commercial assays		
Luciferase Assay System	Promega	Cat. # E1500
SEAP Assay System	Invitrogen	Cat. # T1017
Experimental models: Cell lines		
Mink: TMLC	Abe et al., 1994^47^	N/A
Mouse: MFB-F11	Tesseur, et al., 2006^54^	N/A
Human: Expi293F	Thermo Fisher	Cat. # A14527
Hamster: ExpiCHO-S	Thermo Fisher	Cat. # A29127
Mouse: MFB-F11 with ITGB8	This paper	N/A
Mouse: MFB-F11 with GARP and L-TGF-β1	This paper	N/A
Mouse: MFB-F11 with GARP and L-TGF-β3	This paper	N/A
Mouse: MFB-F11 with GARP and L-TGF-β1_lasso3 chimera	This paper	N/A
Mouse: MFB-F11 with GARP and L-TGF-β1(RGE)	This paper	N/A
Phoenix-AMPHO	ATCC	CRL-3213
Experimental models: Mice		
Mouse: B6(*Gt(ROSA)26Sortm1(PGK1-cre)*Ozg	Ozgene	MGI:5435692
Mouse: 129X1/SvJ	The Jackson Laboratory	Strain #:000691
Mouse: C57BL/6J-*tgfb1em2Lutzy/Mmjax*	The Jackson Laboratory	Strain #:000691
Mouse: B6.129-*tgfb1*^*R278A/R278A*^	This paper	N/A
Oligonucleotides		
5’- tgcacagtacctcatgcaca-3’	This paper	JaxTGFB1F
5’-gaacacagtgctaggcagg-3’	This paper	mTGFB1Ex3R1
5’-ctgtcctggaactcactctgtag-3’	This paper	TGFb1 1F
5’-gtttggatgttgtggtgaagga-3’	This paper	TGFB1 KI/cKI 4R
5’-ccacatttggagaaggac-3’	This paper	TGFb F WT only
5’-catacattatacgaagttatgatctaag-3’	This paper	TGFb F KI only
5’-gacatacacacacttagagg-3’	This paper	TGFb WT/KI rev
5’-gctcagttgggctgttttggag-3’	This paper	ROSAWT F
5’-tagaacagctaaagggtagtgc-3’	This paper	ROSAFlp F
5’-atttacggcgctaaggatgactc-3’	This paper	ROSAcre F
5’-ttacacctgttcaattcccctg-3’	This paper	ROSA R
5’-ctgaaccaaggagacggaatac-3’	This paper	tgfb1 Ex3/4 cDNA F
5’-gttgtagagggcaaggaccttg-3’	This paper	tgfb1 ex 6/7 cDNA R
5’-gcaacaacgccatctatgag-3’	This paper	tgfb1 Ex1/2 cDNA F
5’-gctgatcccgttgatttccac-3’	This paper	tgfb1 Ex4/5 cDNA R
5’-caggtgtcgtgaggctagcatcg-3’	This paper	hTGFB1 Stop F
5’-gcgccactagtctcgagttatcag-3’	This paper	hTGFB1 Stop R
5’-ccctgagccaacggtgatgacccacgtccccgaggccgtgctcgc-3’	This paper	Lasso3 SOE F
5’-tggcgtagtagtcggcctc-3’	This paper	hTGFB1 Bsu36I R
5’-ccatttcaggtgtcgtgaggc-3’	This paper	hTGFB1 F
5’-gtcatcaccgttggctcaggggggctggtgagccgcagcttggacag-3’	This paper	Lasso3 SOE R
5’-ctctgatatcccaagctggctagccacc-3’	This paper	SBPHIS F
5’-cagggcactttgtcttggtgaggaccctgaaacagcacctc-3’	This paper	SBPHIS SOE R
5’-ttagaggtgctgtttcagggtcctcaccaagacaaagtgccctg-3’	This paper	HAGARP SOE F
5’-ccgctgtacaggctgttccc-3’	This paper	HAGARP R
5’-agggccgtgtggacgtgg-3’	This paper	HAGARP Tr F
5’-tctcctcgagttatcagttgatgttcttcagtccccccttc-3’	This paper	HAGARP Tr R
5’-ggggactgaagaacatcaacatgtcgtactaccatcaccatc-3’	This paper	GARP Spy F
5’-ggcttaccttcgaagggcccttagctaccactggatccagta-3’	This paper	GARP Spy R
5’-ccatgtcacacctttcagccc-3’	This paper	TGFB3 R277A F
5’-gtccaaagccgccttcttcctctg-3’	This paper	TGFB3R277ASOER
5’-cagaggaagaaggcggctttggac-3’	This paper	TGFB3R277ASOEF
5’-gtgttgtacagtcccagcacc-3’	This paper	TGFB3 R277A R
5’-ggagaactggggcgcctcaag-3’	This paper	TGFB3 RGE SOE F
5’-ggcgccccagttctccacgg-3’	This paper	TGFB3 RGE SOE R
5’-gtgttgtacagtcccagcacc-3’	This paper	TGFB3 R2
5’-ctctacgcgtactagtggcgcgccgg-3’	This paper	GFP F
5’-ttacttgtacagctcgtccatgcc-3’	This paper	GFP R
5’-gactcactatagggagacccaagctgg-3’	This paper	TGFB3 N Term F
5’-gtccaaggtggtgcaagtggacagggaccctgaaac-3’	This paper	TGFB3SOER
5’-ctgtccacttgcaccaccttggac-3’	This paper	TGFB3SOEF
5’-ggtgagcctaagcttgctcaagatctg-3’	This paper	TGFB3 N Term R
5’-gtccaaggtggtgctagtggacagggaccctgaaac-3’	This paper	TGFB3C4SSOER
5’-ctgtccactagcaccaccttggac-3’	This paper	TGFB3C4SSOEF
Recombinant DNA		
Human TGF-β1_pLX307	Rosenbluh et al., 2016^81^	Addgene, Plasmid #98377
Human TGF-β1 RGE_IRES2 EGFP puro pLX307	This paper	N/A
Human TGF-β1 RGD_R249A_IRES2 EGFP puro pLX307	This paper	N/A
Human TGF-β1 RGE_R249A_IRES2 EGFP puro pLX307	This paper	N/A
Human L-TGF-β1_RGD_Lasso3 puro pLX307	This paper	N/A
		
Integrin αv truncated, αvTr pcDM8	Nishimura et al., 1994^25^	N/A
Integrin β8 truncated, β8Tr pcDNA6	Nishimura et al., 1994^82^	N/A
β8 cDNA pBABE puro	Cambier, et al., 20 00^24^	N/A
HA-GARP pcDNA3	Cuende et al., 2015^78^	N/A
HIS SBP-GARP tr pcDNA6	This paper	N/A
HIS SBP-GARP tr SpyCatcher pcDNA6	This paper	N/A
Integrin αv full length, αvfl pCDVnRa	Nishimura et al., 1994^25^	N/A
Integrin β8 full length, pCDβ8FlNeo	Nishimura et al., 1994^82^	N/A
SpyCatcher	Keeble, et al., 2019^83^	Addgene, Plasmid #133447
pLVE-hTGFβ3-IRES-RED	Brunger, et al., 2014^84^	Addgene, Plasmid #52580
Human TGF-β3 RGD IRES RED pLX307	This paper	N/A
Human TGF-β3 RGD R277A IRES RED pLX307	This paper	N/A
Human TGF-β3 RGE R277A IRES RED pLX307	This paper	N/A
Human TGF-β3 RGE IRES RED pLX307	This paper	N/A
Human TGF-β3 RGD IRES GFP pLX307	This paper	N/A
Human TGF-β3 RGD R277A IRES GFP pLX307	This paper	N/A
Human TGF-β3 RGE R277A IRES GFP pLX307	This paper	N/A
Human TGF-β3 RGE IRES GFP pLX307	This paper	N/A
Human TGF-βR2-Fc	Seed, et al, 2021^27^	N/A
Software and algorithms		
MotionCor2	Zheng et al., 2017^85^	https://msg.ucsf.edu/software; RRID:SCR_016499
Relion 3.0	Zivanov et al., 2018^86^	https://cam.ac.uk/relion; RRID:SCR_016274
SerialEM	Mastronarde, 2005^87^	http://bio3d.colorado.edu/SerialEM/; RRID:SCR_017293
cryoSPARC	Punjani, et al., 2017^88^	https://cryosparc.com; RRID:SCR_016501
PyEM	Daniel Asarnow, Yifan Cheng Lab	https://github.com/asarnow/pyem; https://doi.org/10.5281/zenodo.3576630
UCSF Chimera	Pettersen, et al., 2004^89^	https://www.cgl.ucsf.edu/chimera; RRID:SCR_004097
UCSF ChimeraX	Meng, et al, 2023^90^	https://www.cgl.ucsf.edu/chimerax/ RRID:SCR_015872
COOT	Emsley, et al, 2010^91^	https://www2.mrclmb.cam.ac.uk/personal/pemsley/coot/; RRID:SCR_014222
PHENIX	Adams, et al., 2010^92^	http://www.phenixonline.org; RRID:SCR_014224
Clustal Omega	Madeira, et al., 2019^93^	https://www.ebi.ac.uk/Tools/msa/clustalo/; RRID:SCR_001591
Prism 9	(GraphPad Software, San Diego, CA).	https://www.graphpad.com/scientific-software/prism/; RRID:SCR_002798
SpectroFlo	CyTek Biosciences	https://cytekbio.com/pages/spectro-flo
FlowJo^™^ v10.10	Becton Dickinson and Company	https://www.flowio.com RRID:SCR_008520
Deposited data		
αvβ8/L-TGF-β1/GARP	This study	PDB: 8E4B, EMD-27886, EMD-28061, EMD-28062
L-TGF-β1/GARP	This study	PDB: 8EG9, EMD-2811
L-TGF-β3/GARP	This study	PDB: 8EGC, EMD-28114
αvβ8/L-TGF-β3	This study	PDB: 8EGA, EMD-28112
Other		
QUANTIFOIL^®^ R 1.2/1.3 on Au 300 mesh grids Holey Carbon Film	Quantifoil	Product: N1-C14nAu30-01
UltrAuFoil^®^ R 1.2/1.3 on Au 300 mesh grids Holey Gold Supports	Quantifoil	N1-A14nAu30-01
Quantifoil 400 mesh 1.2/1.3 holey carbon gold grid	Ted Pella	Q425AR-14
Quantifoil 400 mesh 1.2/1.3 holey carbon copper grid	Ted Pella	658-300-CU

## Highlight:

- Mice survive with only autocrine but no paracrine TGF-β1 signaling.
- αvβ8 binding activates L-TGF-β1 for autocrine signaling without release.
- Conformational entropy redistribution drives allosteric activation of L-TGF-β by αvβ8.
- Direction of entropy redistribution can be manipulated by stabilizing flexible domains.

## References

[1] Massagué J, and Sheppard D (2023). TGF-β signaling in health and disease. Cell 186, 4007–4037. 10.1016/j.cell.2023.07.036. 37714133 PMC10772989

[2] Derynck R, Turley SJ, and Akhurst RJ (2020). TGFβ biology in cancer progression and immunotherapy. Nat Rev Clin Oncol. 10.1038/s41571-020-0403-1. PMC9721352 32710082

[3] Akhurst RJ (2017). Targeting TGF-beta Signaling for Therapeutic Gain. Cold Spring Harb Perspect Biol 9. 10.1101/cshperspect.a022301. PMC5630004 28246179

[4] Annes JP, Munger JS, and Rifkin DB (2003). Making sense of latent TGFbeta activation. J Cell Sci 116, 217–224. 12482908 10.1242/jcs.00229

[5] Shi M, Zhu J, Wang R, Chen X, Mi L, Walz T, and Springer TA (2011). Latent TGF-beta structure and activation. Nature 474, 343–349. 10.1038/nature10152. 21677751 PMC4717672

[6] Dubois CM, Blanchette F, Laprise MH, Leduc R, Grondin F, and Seidah NG (2001). Evidence that furin is an authentic transforming growth factor-beta1-converting enzyme. Am J Pathol 158, 305–316. 11141505 10.1016/s0002-9440(10)63970-3 PMC1850265

[7] Wang R, Zhu J, Dong X, Shi M, Lu C, and Springer TA (2012). GARP regulates the bioavailability and activation of TGFbeta. Mol Biol Cell 23, 1129–1139. 10.1091/mbc.E11-12-1018. 22278742 PMC3302739

[8] Stockis J, Colau D, Coulie PG, and Lucas S (2009). Membrane protein GARP is a receptor for latent TGF-beta on the surface of activated human Treg. Eur J Immunol 39, 3315–3322. 10.1002/eji.200939684. 19750484

[9] Lienart S, Merceron R, Vanderaa C, Lambert F, Colau D, Stockis J, van der Woning B, De Haard H, Saunders M, Coulie PG, (2018). Structural basis of latent TGF-beta1 presentation and activation by GARP on human regulatory T cells. Science 362, 952–956. 10.1126/science.aau2909. 30361387

[10] Miyazono K, Olofsson A, Colosetti P, and Heldin CH (1991). A role of the latent TGF-beta 1-binding protein in the assembly and secretion of TGF-beta 1. EMBO J 10, 1091–1101. 2022183 10.1002/j.1460-2075.1991.tb08049.x PMC452762

[11] Aluwihare P, Mu Z, Zhao Z, Yu D, Weinreb PH, Horan GS, Violette SM, and Munger JS (2009). Mice that lack activity of alphavbeta6- and alphavbeta8-integrins reproduce the abnormalities of Tgfb1- and Tgfb3-null mice. J Cell Sci 122, 227–232. 122/2/2 [pii] 10.1242/jcs.035246. 19118215 PMC2714418

[12] Moses HL, Roberts AB, and Derynck R (2016). The Discovery and Early Days of TGF-beta: A Historical Perspective. Cold Spring Harb Perspect Biol 8. 10.1101/cshperspect.a021865. PMC4930926 27328871

[13] Qian SW, Burmester JK, Tsang ML, Weatherbee JA, Hinck AP, Ohlsen DJ, Sporn MB, and Roberts AB (1996). Binding affinity of transforming growth factor-beta for its type II receptor is determined by the C-terminal region of the molecule. J Biol Chem 271, 30656–30662. 8940041 10.1074/jbc.271.48.30656

[14] Bertoli-Avella AM, Gillis E, Morisaki H, Verhagen JMA, de Graaf BM, van de Beek G, Gallo E, Kruithof BPT, Venselaar H, Myers LA, (2015). Mutations in a TGF-β ligand, TGFB3, cause syndromic aortic aneurysms and dissections. J Am Coll Cardiol 65, 1324–1336. 10.1016/j.jacc.2015.01.040. 25835445 PMC4380321

[15] Shull MM, Ormsby I, Kier AB, Pawlowski S, Diebold RJ, Yin M, Allen R, Sidman C, Proetzel G, Calvin D, and (1992). Targeted disruption of the mouse transforming growth factor-beta 1 gene results in multifocal inflammatory disease. Nature 359, 693–699. 10.1038/359693a0. 1436033 PMC3889166

[16] Kaartinen V, Voncken JW, Shuler C, Warburton D, Bu D, Heisterkamp N, and Groffen J (1995). Abnormal lung development and cleft palate in mice lacking TGF-beta 3 indicates defects of epithelial-mesenchymal interaction. Nat Genet 11, 415–421. 10.1038/ng1295-415. 7493022

[17] Schepers D, Tortora G, Morisaki H, MacCarrick G, Lindsay M, Liang D, Mehta SG, Hague J, Verhagen J, van de Laar I, (2018). A mutation update on the LDS-associated genes TGFB2/3 and SMAD2/3. Hum Mutat 39, 621–634. 10.1002/humu.23407. 29392890 PMC5947146

[18] Mu D, Cambier S, Fjellbirkeland L, Baron JL, Munger JS, Kawakatsu H, Sheppard D, Broaddus VC, and Nishimura SL (2002). The integrin alpha(v)beta8 mediates epithelial homeostasis through MT1-MMP-dependent activation of TGF-beta1. J Cell Biol 157, 493–507. 10.1083/jcb.200109100. 11970960 PMC2173277

[19] Munger JS, Huang X, Kawakatsu H, Griffiths MJ, Dalton SL, Wu J, Pittet JF, Kaminski N, Garat C, Matthay MA, (1999). The integrin alpha v beta 6 binds and activates latent TGF beta 1: a mechanism for regulating pulmonary inflammation and fibrosis. Cell 96, 319–328. 10025398 10.1016/s0092-8674(00)80545-0

[20] Li MO, Wan YY, and Flavell RA (2007). T cell-produced transforming growth factor-beta1 controls T cell tolerance and regulates Th1- and Th17-cell differentiation. Immunity 26, 579–591. 10.1016/j.immuni.2007.03.014. 17481928

[21] Bhowmick NA, Chytil A, Plieth D, Gorska AE, Dumont N, Shappell S, Washington MK, Neilson EG, and Moses HL (2004). TGF-beta signaling in fibroblasts modulates the oncogenic potential of adjacent epithelia. Science 303, 848–851. 10.1126/science.1090922. 14764882

[22] Campbell MG, Cormier A, Ito S, Seed RI, Bondesson AJ, Lou J, Marks JD, Baron JL, Cheng Y, and Nishimura SL (2020). Cryo-EM Reveals Integrin-Mediated TGF-β Activation without Release from Latent TGF-β. Cell 180, 490–501.e416. 10.1016/j.cell.2019.12.030. 31955848 PMC7238552

[23] Dong X, Zhao B, Iacob RE, Zhu J, Koksal AC, Lu C, Engen JR, and Springer TA (2017). Force interacts with macromolecular structure in activation of TGF-beta. Nature 542, 55–59. 10.1038/nature21035. 28117447 PMC5586147

[24] Cambier S, Mu DZ, O’Connell D, Boylen K, Travis W, Liu WH, Broaddus VC, and Nishimura SL (2000). A role for the integrin alphavbeta8 in the negative regulation of epithelial cell growth. Cancer research 60, 7084–7093. 11156415

[25] Nishimura SL, Sheppard D, and Pytela R (1994). Integrin alpha v beta 8. Interaction with vitronectin and functional divergence of the beta 8 cytoplasmic domain. J Biol Chem 269, 28708–28715. 7525578

[26] Annes JP, Rifkin DB, and Munger JS (2002). The integrin alphaVbeta6 binds and activates latent TGFbeta3. FEBS Lett 511, 65–68. 10.1016/s0014-5793(01)03280-x. 11821050

[27] Seed RI, Kobayashi K, Ito S, Takasaka N, Cormier A, Jespersen JM, Publicover J, Trilok S, Combes AJ, Chew NW, (2021). A tumor-specific mechanism of Treg enrichment mediated by the integrin αvβ8. Sci Immunol 6. 10.1126/sciimmunol.abf0558. PMC8425767 33771888

[28] Cormier A, Campbell MG, Ito S, Wu S, Lou J, Marks J, Baron JL, Nishimura SL, and Cheng Y (2018). Cryo-EM structure of the alphavbeta8 integrin reveals a mechanism for stabilizing integrin extension. Nat Struct Mol Biol 25, 698–704. 10.1038/s41594-018-0093-x. 30061598 PMC6214843

[29] Wang J, Dong X, Zhao B, Li J, Lu C, and Springer TA (2017). Atypical interactions of integrin alphaVbeta8 with pro-TGF-beta1. Proc Natl Acad Sci U S A 114, E4168–E4174. 10.1073/pnas.1705129114. 28484027 PMC5448207

[30] Kulkarni AB, Huh CG, Becker D, Geiser A, Lyght M, Flanders KC, Roberts AB, Sporn MB, Ward JM, and Karlsson S (1993). Transforming growth factor beta 1 null mutation in mice causes excessive inflammatory response and early death. Proc Natl Acad Sci U S A 90, 770–774. 10.1073/pnas.90.2.770. 8421714 PMC45747

[31] Marie JC, Liggitt D, and Rudensky AY (2006). Cellular mechanisms of fatal early-onset autoimmunity in mice with the T cell-specific targeting of transforming growth factor-beta receptor. Immunity 25, 441–454. 10.1016/j.immuni.2006.07.012. 16973387

[32] Li MO, Sanjabi S, and Flavell RA (2006). Transforming growth factor-beta controls development, homeostasis, and tolerance of T cells by regulatory T cell-dependent and - independent mechanisms. Immunity 25, 455–471. 10.1016/j.immuni.2006.07.011. 16973386

[33] Tang Y, McKinnon ML, Leong LM, Rusholme SA, Wang S, and Akhurst RJ (2003). Genetic modifiers interact with maternal determinants in vascular development of Tgfb1(−/−) mice. Hum Mol Genet 12, 1579–1589. 10.1093/hmg/ddg164. 12812985

[34] Letterio JJ, Geiser AG, Kulkarni AB, Roche NS, Sporn MB, and Roberts AB (1994). Maternal rescue of transforming growth factor-beta 1 null mice. Science 264, 1936–1938. 10.1126/science.8009224. 8009224

[35] Martinez L (2015). Automatic identification of mobile and rigid substructures in molecular dynamics simulations and fractional structural fluctuation analysis. PLoS One 10, e0119264. 10.1371/journal.pone.0119264. 25816325 PMC4376797

[36] Monod J, Wyman J, and Changeux JP (1965). On The nature of allosteric transitions: a plausible model. J Mol Biol 12, 88–118. 10.1016/s0022-2836(65)80285-6. 14343300

[37] Cooper A, and Dryden DT (1984). Allostery without conformational change. A plausible model. Eur Biophys J 11, 103–109. 10.1007/BF00276625. 6544679

[38] Saavedra HG, Wrabl JO, Anderson JA, Li J, and Hilser VJ (2018). Dynamic allostery can drive cold adaptation in enzymes. Nature 558, 324–328. 10.1038/s41586-018-0183-2. 29875414 PMC6033628

[39] Petit CM, Zhang J, Sapienza PJ, Fuentes EJ, and Lee AL (2009). Hidden dynamic allostery in a PDZ domain. Proc Natl Acad Sci U S A 106, 18249–18254. 10.1073/pnas.0904492106. 19828436 PMC2775317

[40] Fuentes EJ, Der CJ, and Lee AL (2004). Ligand-dependent dynamics and intramolecular signaling in a PDZ domain. J Mol Biol 335, 1105–1115. 10.1016/j.jmb.2003.11.010. 14698303

[41] Motlagh HN, Wrabl JO, Li J, and Hilser VJ (2014). The ensemble nature of allostery. Nature 508, 331–339. 10.1038/nature13001. 24740064 PMC4224315

[42] Doig AJ, and Sternberg MJ (1995). Side-chain conformational entropy in protein folding. Protein Sci 4, 2247–2251. 10.1002/pro.5560041101. 8563620 PMC2143028

[43] Tzeng SR, and Kalodimos CG (2012). Protein activity regulation by conformational entropy. Nature 488, 236–240. 10.1038/nature11271. 22801505

[44] Wankowicz SA, de Oliveira SH, Hogan DW, van den Bedem H, and Fraser JS (2022). Ligand binding remodels protein side-chain conformational heterogeneity. Elife 11. 10.7554/eLife.74114. PMC9084896 35312477

[45] Capdevila DA, Braymer JJ, Edmonds KA, Wu H, and Giedroc DP (2017). Entropy redistribution controls allostery in a metalloregulatory protein. Proc Natl Acad Sci U S A 114, 4424–4429. 10.1073/pnas.1620665114. 28348247 PMC5410788

[46] Wankowicz SA, and Fraser JS (2023). Making sense of chaos: uncovering the mechanisms of conformational entropy. ChemRxiv. 10.26434/chemrxiv-2023-9b5k7. PMC12103944 40275100

[47] Abe M, Harpel JG, Metz CN, Nunes I, Loskutoff DJ, and Rifkin DB (1994). An assay for transforming growth factor-beta using cells transfected with a plasminogen activator inhibitor-1 promoter-luciferase construct. Anal Biochem 216, 276–284. 10.1006/abio.1994.1042. 8179182

[48] Li Z (2019). Truncation of TGF-beta docking receptor GARP is linked to human disease. Eur J Hum Genet. 10.1038/s41431-019-0411-8. PMC6777629 31053781

[49] Harel T, Levy-Lahad E, Daana M, Mechoulam H, Horowitz-Cederboim S, Gur M, Meiner V, and Elpeleg O (2019). Homozygous stop-gain variant in LRRC32, encoding a TGFβ receptor, associated with cleft palate, proliferative retinopathy, and developmental delay. Eur J Hum Genet 27, 1315–1319. 10.1038/s41431-019-0380-y. 30976112 PMC6777458

[50] Wu BX, Li A, Lei L, Kaneko S, Wallace C, Li X, and Li Z (2017). Glycoprotein A repetitions predominant (GARP) positively regulates transforming growth factor (TGF) beta3 and is essential for mouse palatogenesis. J Biol Chem 292, 18091–18097. 10.1074/jbc.M117.797613. 28912269 PMC5672034

[51] Okamura T, Sumitomo S, Morita K, Iwasaki Y, Inoue M, Nakachi S, Komai T, Shoda H, Miyazaki J, Fujio K, and Yamamoto K (2015). TGF-β3-expressing CD4+CD25(−)LAG3+ regulatory T cells control humoral immune responses. Nat Commun 6, 6329. 10.1038/ncomms7329. 25695838 PMC4346620

[52] Le VQ, Iacob RE, Zhao B, Su Y, Tian Y, Toohey C, Engen JR, and Springer TA (2022). Protection of the Prodomain α1-Helix Correlates with Latency in the Transforming Growth Factor-β Family. J Mol Biol 434, 167439. 10.1016/j.jmb.2021.167439. 34990654 PMC8981510

[53] Radaev S, Zou Z, Huang T, Lafer EM, Hinck AP, and Sun PD (2010). Ternary complex of transforming growth factor-beta1 reveals isoform-specific ligand recognition and receptor recruitment in the superfamily. J Biol Chem 285, 14806–14814. 10.1074/jbc.M109.079921. 20207738 PMC2863181

[54] Tesseur I, Zou K, Berber E, Zhang H, and Wyss-Coray T (2006). Highly sensitive and specific bioassay for measuring bioactive TGF-beta. BMC Cell Biol 7, 15. 10.1186/1471-2121-7-15. 16549026 PMC1479809

[55] Hart PJ, Deep S, Taylor AB, Shu Z, Hinck CS, and Hinck AP (2002). Crystal structure of the human TbetaR2 ectodomain--TGF-beta3 complex. Nat Struct Biol 9, 203–208. 10.1038/nsb766. 11850637

[56] Batlle E, and Massague J (2019). Transforming Growth Factor-beta Signaling in Immunity and Cancer. Immunity 50, 924–940. 10.1016/j.immuni.2019.03.024. 30995507 PMC7507121

[57] Miyazono K, Hellman U, Wernstedt C, and Heldin CH (1988). Latent high molecular weight complex of transforming growth factor beta 1. Purification from human platelets and structural characterization. J Biol Chem 263, 6407–6415. 3162913

[58] Shi Y, and Massague J (2003). Mechanisms of TGF-beta signaling from cell membrane to the nucleus. Cell 113, 685–700. 12809600 10.1016/s0092-8674(03)00432-x

[59] Rifkin DB (2005). Latent transforming growth factor-beta (TGF-beta) binding proteins: orchestrators of TGF-beta availability. J Biol Chem 280, 7409–7412. 10.1074/jbc.R400029200. 15611103

[60] Yoshinaga K, Obata H, Jurukovski V, Mazzieri R, Chen Y, Zilberberg L, Huso D, Melamed J, Prijatelj P, Todorovic V, (2008). Perturbation of transforming growth factor (TGF)-beta1 association with latent TGF-beta binding protein yields inflammation and tumors. Proc Natl Acad Sci U S A 105, 18758–18763. 10.1073/pnas.0805411105. 19022904 PMC2596235

[61] Qin Y, Garrison BS, Ma W, Wang R, Jiang A, Li J, Mistry M, Bronson RT, Santoro D, Franco C, (2018). A Milieu Molecule for TGF-β Required for Microglia Function in the Nervous System. Cell 174, 156–171.e116. 10.1016/j.cell.2018.05.027. 29909984 PMC6089614

[62] He Z, Khatib AM, and Creemers JWM (2022). The proprotein convertase furin in cancer: more than an oncogene. Oncogene 41, 1252–1262. 10.1038/s41388-021-02175-9. 34997216

[63] Yang LT, and Kaartinen V (2007). Tgfb1 expressed in the Tgfb3 locus partially rescues the cleft palate phenotype of Tgfb3 null mutants. Dev Biol 312, 384–395. 10.1016/j.ydbio.2007.09.034. 17967447 PMC2174429

[64] Fitzpatrick DR, Denhez F, Kondaiah P, and Akhurst RJ (1990). Differential expression of TGF beta isoforms in murine palatogenesis. Development 109, 585–595. 10.1242/dev.109.3.585. 2401212

[65] Marsili L, Overwater E, Hanna N, Baujat G, Baars MJH, Boileau C, Bonneau D, Brehin AC, Capri Y, Cheung HY, (2020). Phenotypic spectrum of TGFB3 disease-causing variants in a Dutch-French cohort and first report of a homozygous patient. Clin Genet 97, 723–730. 10.1111/cge.13700. 31898322

[66] Perik MHAM, Govaerts E, Laga S, Goovaerts I, Saenen J, Van Craenenbroeck E, Meester JAN, Luyckx I, Rodrigus I, Verstraeten A, (2023). Variable clinical expression of a Belgian. Front Genet 14, 1251675. 10.3389/fgene.2023.1251675. 37719708 PMC10500191

[67] Zhu J, Motejlek K, Wang D, Zang K, Schmidt A, and Reichardt LF (2002). beta8 integrins are required for vascular morphogenesis in mouse embryos. Development 129, 2891–2903. 12050137 10.1242/dev.129.12.2891 PMC2710098

[68] Bader BL, Rayburn H, Crowley D, and Hynes RO (1998). Extensive vasculogenesis, angiogenesis, and organogenesis precede lethality in mice lacking all alpha v integrins. Cell 95, 507–519. 10.1016/s0092-8674(00)81618-9. 9827803

[69] Ge G, Hopkins DR, Ho WB, and Greenspan DS (2005). GDF11 forms a bone morphogenetic protein 1-activated latent complex that can modulate nerve growth factor-induced differentiation of PC12 cells. Mol Cell Biol 25, 5846–5858. 10.1128/MCB.25.14.5846-5858.2005. 15988002 PMC1168807

[70] Thies RS, Chen T, Davies MV, Tomkinson KN, Pearson AA, Shakey QA, and Wolfman NM (2001). GDF-8 propeptide binds to GDF-8 and antagonizes biological activity by inhibiting GDF-8 receptor binding. Growth Factors 18, 251–259. 10.3109/08977190109029114. 11519824

[71] Lan Y, Zhang D, Xu C, Hance KW, Marelli B, Qi J, Yu H, Qin G, Sircar A, Hernandez VM, (2018). Enhanced preclinical antitumor activity of M7824, a bifunctional fusion protein simultaneously targeting PD-L1 and TGF-beta. Sci Transl Med 10. 10.1126/scitranslmed.aan5488. 29343622

[72] Lacouture ME, Morris JC, Lawrence DP, Tan AR, Olencki TE, Shapiro GI, Dezube BJ, Berzofsky JA, Hsu FJ, and Guitart J (2015). Cutaneous keratoacanthomas/squamous cell carcinomas associated with neutralization of transforming growth factor beta by the monoclonal antibody fresolimumab (GC1008). Cancer Immunol Immunother 64, 437–446. 10.1007/s00262-015-1653-0. 25579378 PMC6730642

[73] Mitra MS, Lancaster K, Adedeji AO, Palanisamy GS, Dave RA, Zhong F, Holdren MH, Turley SJ, Liang WC, Wu Y, (2020). A Potent Pan-TGFβ Neutralizing Monoclonal Antibody Elicits Cardiovascular Toxicity in Mice and Cynomolgus Monkeys. Toxicol Sci. 10.1093/toxsci/kfaa024. 32077954

[74] Tolcher AW, Berlin JD, Cosaert J, Kauh J, Chan E, Piha-Paul SA, Amaya A, Tang S, Driscoll K, Kimbung R, (2017). A phase 1 study of anti-TGFbeta receptor type-II monoclonal antibody LY3022859 in patients with advanced solid tumors. Cancer Chemother Pharmacol 79, 673–680. 10.1007/s00280-017-3245-5. 28280971 PMC5893148

[75] Anderton MJ, Mellor HR, Bell A, Sadler C, Pass M, Powell S, Steele SJ, Roberts RR, and Heier A (2011). Induction of heart valve lesions by small-molecule ALK5 inhibitors. Toxicol Pathol 39, 916–924. 0192623311416259 [pii] 10.1177/0192623311416259. 21859884

[76] Cho BC, Lee JS, Wu YL, Cicin I, Dols MC, Ahn MJ, Cuppens K, Veillon R, Nadal E, Dias JM, (2023). Bintrafusp Alfa Versus Pembrolizumab in Patients With Treatment-Naive, Programmed Death-Ligand 1-High Advanced NSCLC: A Randomized, Open-Label, Phase 3 Trial. J Thorac Oncol 18, 1731–1742. 10.1016/j.jtho.2023.08.018. 37597750

[77] Ciardiello D, Elez E, Tabernero J, and Seoane J (2020). Clinical development of therapies targeting TGFβ: current knowledge and future perspectives. Ann Oncol 31, 1336–1349. 10.1016/j.annonc.2020.07.009. 32710930

[78] Cuende J, Lienart S, Dedobbeleer O, van der Woning B, De Boeck G, Stockis J, Huygens C, Colau D, Somja J, Delvenne P, (2015). Monoclonal antibodies against GARP/TGF-beta1 complexes inhibit the immunosuppressive activity of human regulatory T cells in vivo. Sci Transl Med 7, 284ra256. 10.1126/scitranslmed.aaa1983. 25904740

[79] Gabriely G, da Cunha AP, Rezende RM, Kenyon B, Madi A, Vandeventer T, Skillin N, Rubino S, Garo L, Mazzola MA, (2017). Targeting latency-associated peptide promotes antitumor immunity. Sci Immunol 2. 10.1126/sciimmunol.aaj1738. PMC5657397 28763794

[80] Takasaka N, Seed RI, Cormier A, Bondesson AJ, Lou J, Elattma A, Ito S, Yanagisawa H, Hashimoto M, Ma R, (2018). Integrin alphavbeta8-expressing tumor cells evade host immunity by regulating TGF-beta activation in immune cells. JCI Insight 3. 10.1172/jci.insight.122591. PMC6237456 30333313

[81] Rosenbluh J, Mercer J, Shrestha Y, Oliver R, Tamayo P, Doench JG, Tirosh I, Piccioni F, Hartenian E, Horn H, (2016). Genetic and Proteomic Interrogation of Lower Confidence Candidate Genes Reveals Signaling Networks in β-Catenin-Active Cancers. Cell Syst 3, 302–316.e304. 10.1016/j.cels.2016.09.001. 27684187 PMC5455996

[82] Araya J, Cambier S, Markovics JA, Wolters P, Jablons D, Hill A, Finkbeiner W, Jones K, Broaddus VC, Sheppard D, (2007). Squamous metaplasia amplifies pathologic epithelial-mesenchymal interactions in COPD patients. J Clin Invest 117, 3551–3562. 10.1172/JCI32526. 17965775 PMC2040320

[83] Keeble AH, and Howarth M (2019). Insider information on successful covalent protein coupling with help from SpyBank. Methods Enzymol 617, 443–461. 10.1016/bs.mie.2018.12.010. 30784412

[84] Brunger JM, Huynh NP, Guenther CM, Perez-Pinera P, Moutos FT, Sanchez-Adams J, Gersbach CA, and Guilak F (2014). Scaffold-mediated lentiviral transduction for functional tissue engineering of cartilage. Proc Natl Acad Sci U S A 111, E798–806. 10.1073/pnas.1321744111. 24550481 PMC3948308

[85] Zheng SQ, Palovcak E, Armache JP, Verba KA, Cheng Y, and Agard DA (2017). MotionCor2: anisotropic correction of beam-induced motion for improved cryo-electron microscopy. Nat Methods 14, 331–332. 10.1038/nmeth.4193. 28250466 PMC5494038

[86] Zivanov J, Nakane T, Forsberg BO, Kimanius D, Hagen WJ, Lindahl E, and Scheres SH (2018). New tools for automated high-resolution cryo-EM structure determination in RELION-3. Elife 7. 10.7554/eLife.42166. PMC6250425 30412051

[87] Mastronarde DN (2005). Automated electron microscope tomography using robust prediction of specimen movements. J Struct Biol 152, 36–51. 10.1016/j.jsb.2005.07.007. 16182563

[88] Punjani A, Rubinstein JL, Fleet DJ, and Brubaker MA (2017). cryoSPARC: algorithms for rapid unsupervised cryo-EM structure determination. Nat Methods 14, 290–296. 10.1038/nmeth.4169. 28165473

[89] Pettersen EF, Goddard TD, Huang CC, Couch GS, Greenblatt DM, Meng EC, and Ferrin TE (2004). UCSF Chimera--a visualization system for exploratory research and analysis. J Comput Chem 25, 1605–1612. 10.1002/jcc.20084. 15264254

[90] Meng EC, Goddard TD, Pettersen EF, Couch GS, Pearson ZJ, Morris JH, and Ferrin TE (2023). UCSF ChimeraX: Tools for structure building and analysis. Protein Sci 32, e4792. 10.1002/pro.4792. 37774136 PMC10588335

[91] Emsley P, Lohkamp B, Scott WG, and Cowtan K (2010). Features and development of Coot. Acta Crystallogr D Biol Crystallogr 66, 486–501. 10.1107/S0907444910007493. 20383002 PMC2852313

[92] Adams PD, Afonine PV, Bunkóczi G, Chen VB, Davis IW, Echols N, Headd JJ, Hung LW, Kapral GJ, Grosse-Kunstleve RW, (2010). PHENIX: a comprehensive Python-based system for macromolecular structure solution. Acta Crystallogr D Biol Crystallogr 66, 213–221. 10.1107/S0907444909052925. 20124702 PMC2815670

[93] Madeira F, Park YM, Lee J, Buso N, Gur T, Madhusoodanan N, Basutkar P, Tivey ARN, Potter SC, Finn RD, and Lopez R (2019). The EMBL-EBI search and sequence analysis tools APIs in 2019. Nucleic Acids Res. 10.1093/nar/gkz268. PMC6602479 30976793

[94] Yang Z, Mu Z, Dabovic B, Jurukovski V, Yu D, Sung J, Xiong X, and Munger JS (2007). Absence of integrin-mediated TGFbeta1 activation in vivo recapitulates the phenotype of TGFbeta1-null mice. J Cell Biol 176, 787–793. 10.1083/jcb.200611044. 17353357 PMC2064053

[95] Anthis NJ, Wegener KL, Ye F, Kim C, Goult BT, Lowe ED, Vakonakis I, Bate N, Critchley DR, Ginsberg MH, and Campbell ID (2009). The structure of an integrin/talin complex reveals the basis of inside-out signal transduction. EMBO J 28, 3623–3632. 10.1038/emboj.2009.287. 19798053 PMC2782098

[96] Weinacker A, Chen A, Agrez M, Cone RI, Nishimura S, Wayner E, Pytela R, and Sheppard D (1994). Role of the integrin alpha v beta 6 in cell attachment to fibronectin. Heterologous expression of intact and secreted forms of the receptor. J Biol Chem 269, 6940–6948. 8120056

[97] Wang F, Liu Y, Yu Z, Li S, Feng S, Cheng Y, and Agard DA (2020). General and robust covalently linked graphene oxide affinity grids for high-resolution cryo-EM. Proc Natl Acad Sci U S A 117, 24269–24273. 10.1073/pnas.2009707117. 32913054 PMC7533693

[98] Sanchez-Garcia R, Gomez-Blanco J, Cuervo A, Carazo JM, Sorzano COS, and Vargas J (2021). DeepEMhancer: a deep learning solution for cryo-EM volume post-processing. Commun Biol 4, 874. 10.1038/s42003-021-02399-1. 34267316 PMC8282847

[99] Scheres SH (2012). RELION: implementation of a Bayesian approach to cryo-EM structure determination. J Struct Biol 180, 519–530. 10.1016/j.jsb.2012.09.006. 23000701 PMC3690530

[100] Waterhouse A, Bertoni M, Bienert S, Studer G, Tauriello G, Gumienny R, Heer FT, de Beer TAP, Rempfer C, Bordoli L, (2018). SWISS-MODEL: homology modelling of protein structures and complexes. Nucleic Acids Res 46, W296–W303. 10.1093/nar/gky427. 29788355 PMC6030848

[101] Dong X, Hudson NE, Lu C, and Springer TA (2014). Structural determinants of integrin beta-subunit specificity for latent TGF-beta. Nat Struct Mol Biol 21, 1091–1096. 10.1038/nsmb.2905. 25383667 PMC4717663

[102] Liebschner D, Afonine PV, Baker ML, Bunkóczi G, Chen VB, Croll TI, Hintze B, Hung LW, Jain S, McCoy AJ, (2019). Macromolecular structure determination using X-rays, neutrons and electrons: recent developments in Phenix. Acta Crystallogr D Struct Biol 75, 861–877. 10.1107/S2059798319011471. 31588918 PMC6778852

[103] Croll TI (2018). ISOLDE: a physically realistic environment for model building into low-resolution electron-density maps. Acta Crystallogr D Struct Biol 74, 519–530. 10.1107/S2059798318002425. 29872003 PMC6096486

[104] Emsley P, and Cowtan K (2004). Coot: model-building tools for molecular graphics. Acta Crystallogr D Biol Crystallogr 60, 2126–2132. 10.1107/S0907444904019158. 15572765

[105] Goddard TD, Huang CC, Meng EC, Pettersen EF, Couch GS, Morris JH, and Ferrin TE (2018). UCSF ChimeraX: Meeting modern challenges in visualization and analysis. Protein Sci 27, 14–25. 10.1002/pro.3235. 28710774 PMC5734306

[106] Rohl CA, Strauss CE, Chivian D, and Baker D (2004). Modeling structurally variable regions in homologous proteins with rosetta. Proteins 55, 656–677. 10.1002/prot.10629. 15103629

[107] Michaud-Agrawal N, Denning EJ, Woolf TB, and Beckstein O (2011). MDAnalysis: a toolkit for the analysis of molecular dynamics simulations. J Comput Chem 32, 2319–2327. 10.1002/jcc.21787. 21500218 PMC3144279

[108] Jorgensen WL, Chandrasekhar J, and Madura JD (1983). JOURNAL OF CHEMICAL PHYSICS. 79, 926.

[109] Abraham MJ, Murtola T, Schulz R, Páll S, Smith JC, Hess B, and Lindahl E (2015). GROMACS: High performance molecular simulations through multi-level parallelism from laptops to supercomputers. Elsevier.

[110] Essmann U, Perera L, Berkowitz ML, Darden T, Lee H, and Pedersen LG (1995). A smooth particle mesh Ewald method.

[111] Hess B (2008). P-LINCS: A Parallel Linear Constraint Solver for Molecular Simulation. J Chem Theory Comput 4, 116–122. 10.1021/ct700200b. 26619985

