# Supplementary figures and images for: Dynamic allostery drives autocrine and paracrine TGF-β signaling
Source: Cell. Author manuscript; Available in PMC 2024 Nov 3. (PMC11531391; doi:10.1016/j.cell.2024.08.036)

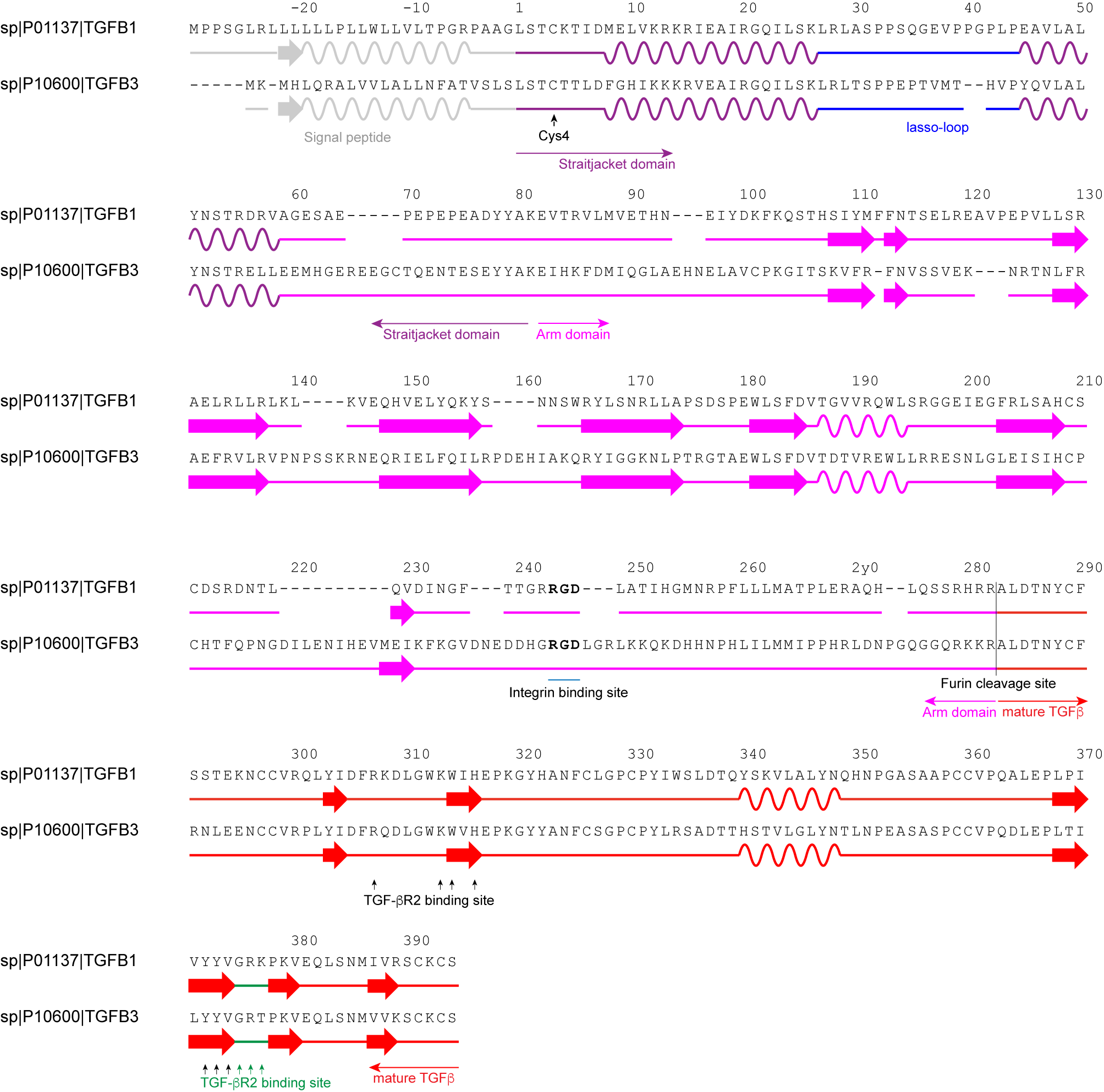

Supplement: 1 — Figure S1. Domain organization of L-TGF-β1 and -β3, related to Introduction, Figure 2 and Figure 4. Sequence alignment with secondary structure prediction of L-TGF-β1 and L-TGF-β3. Domains are colored as in (Figure 2A) and secondary structure prediction marked. Sequence numbering does not include signal peptide and is consistent in all figures and text. [file NIHMS2024290-supplement-1.tif]

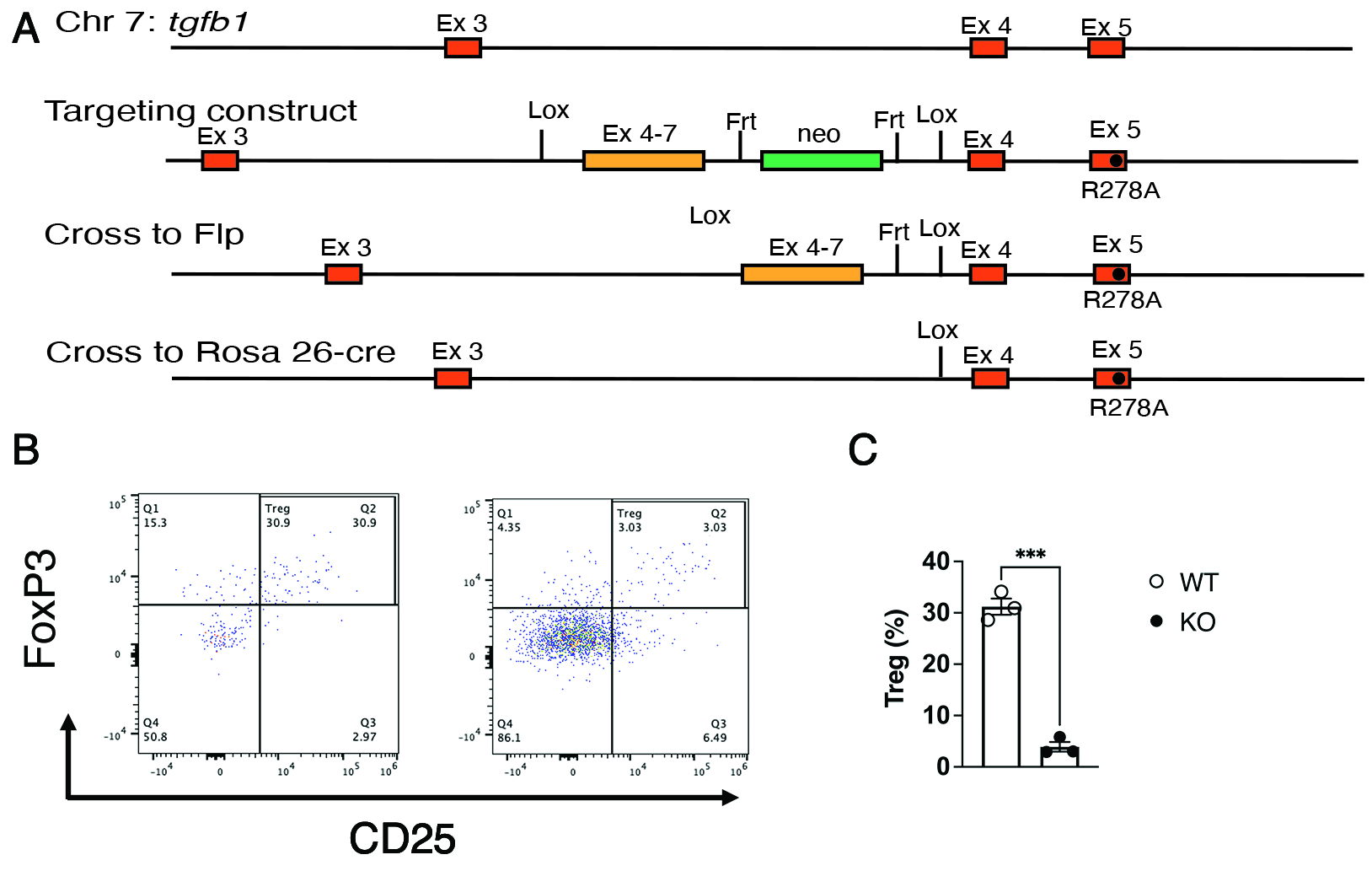

Supplement: 2 — Figure S2. Engineering of tgfb1 R278A KI/KI mice and immune characterization of tgfb1 −/− mice, related to Figure 1. (A) From top to bottom, schematic of the tgfb1 targeted locus, targeting construct after germline integration, after crossing to mice expressing flippase (flp), after crossing to rosa 26-cre. (B) Scatter plots showing Treg from PBMC isolated from post-natal day 18 tgfb1 −/− (KO/KO) mice compared with day 18 littermate controls (WT/KO). Staining done as in Figure 1. (C) Scatter bar graphs showing Treg as a percentage of CD4+ T-cells (n=3 KO/KO; n=3 WT/KO). Shown is SE. *** p<0.001. [file NIHMS2024290-supplement-2.tif]

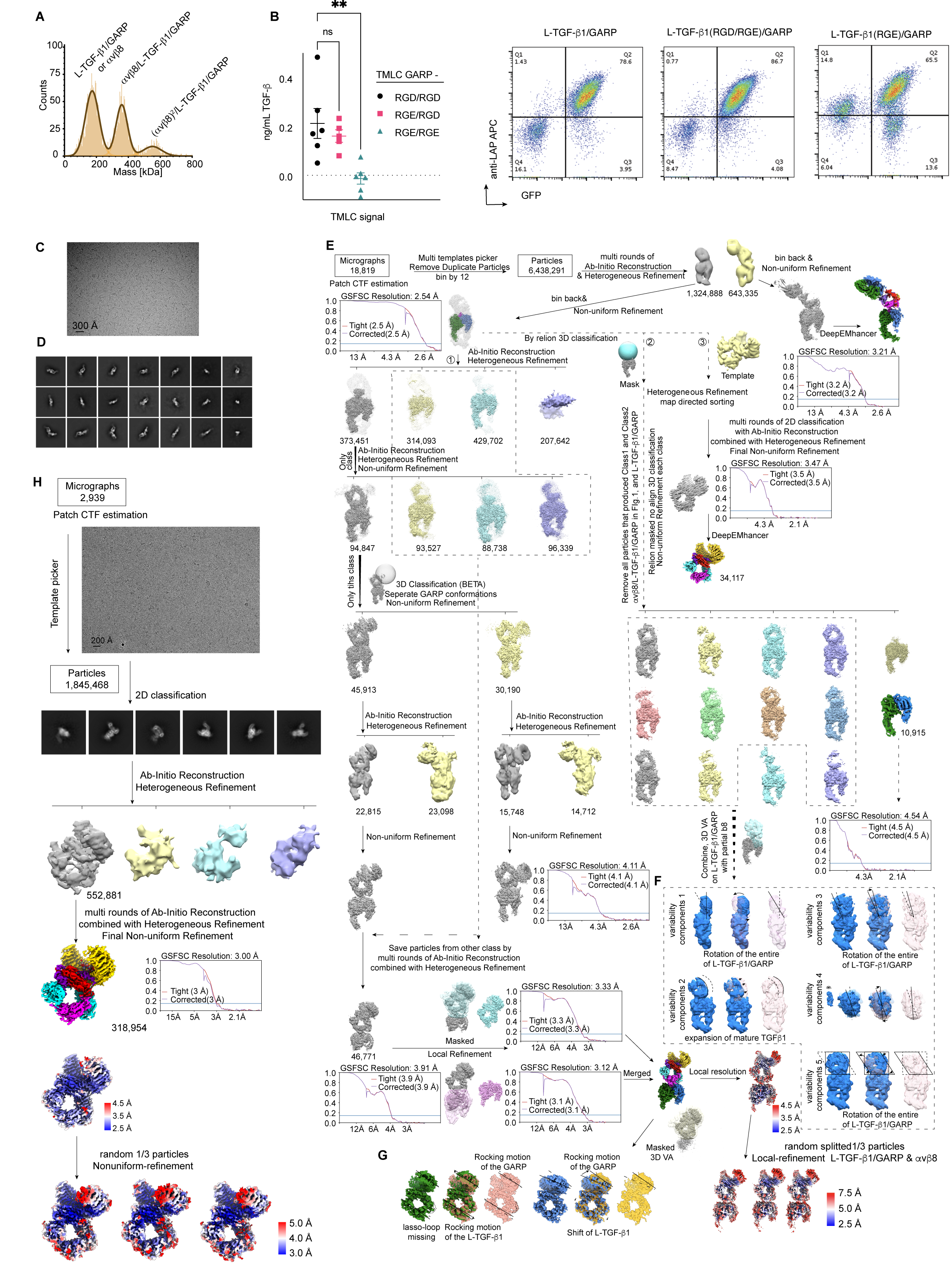

Supplement: 3 — Figure S3. Single particle cryo-EM and image processing of L-TGF-β1/GARP and αvβ8/L-TGF-β1/GARP complex, related to Figure 2. (A) Mass photometry analysis of L-TGF-β1/GARP mixed with ectodomain of αvβ8. Three peaks correspond to L-TGF-β1/GARP or αvβ8 alone at ~180kD, one L-TGF-β1/GARP bound with one αvβ8 at ~380kD, and one L-TGF-β1/GARP bound with two αvβ8 integrins at ~560kD. (B) Co-culture of MFB-F11 αvβ8 expressing cells with TMLC L-TGF-β1/GARP presenting cells is sufficient to activate the TGF-β signaling pathway by TMLC. To test whether one RGD site is sufficient for αvβ8-mediated TGF-β activation, TMLC-L- TGF-β1/GARP cells were made using a 1:1 transfection ratio of TGF- β1(RGD) and TGF- β1(RGE) (pink squares). TMLC: L- TGF-β1(RGE/RGE)/GARP cells are also included to demonstrate the requirement of RGD for αvβ8/L-TGF- β1 binding and activation (green triangles). The results (vertical axis) are normalized against a standard TGF-β activation curve. **p<0.01 by one-way ANOVA followed by Sidak’s multiple comparison test for the indicated comparisons (C) Representative electron micrograph of frozen hydrated sample of mixing αvβ8 with L-TGF-β1/GARP in 1:1 molar ratio. (D) Representative 2D class averages calculated from particles selected for further processing. (E) Flow-chart of cryo-EM data processing. FSC curves for resolution estimations are included at various steps of the flow-chart. (F) Motions captured in αvβ8/L-TGF-β1/GARP complex along five eigenvectors from 3DVA are illustrated. (G) 3DVA of particles in Class 1 in Figure 2F. (H) Image processing flow-chart from representative electron micrograph to the final reconstruction and FSC for final resolution estimation of L-TGF-β1/GARP. [file NIHMS2024290-supplement-3.tif]

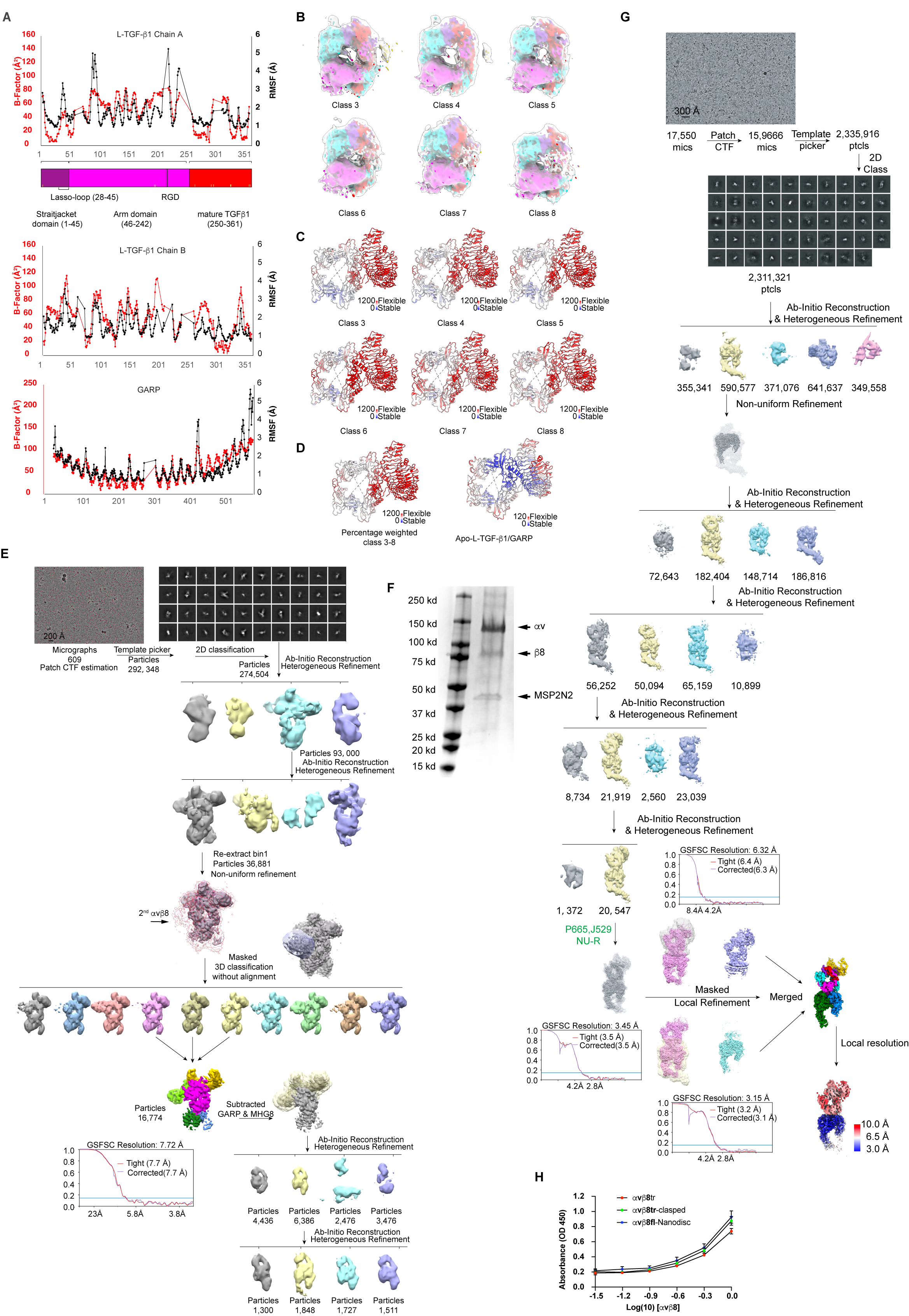

Supplement: 4 — Figure S4. Single particle cryo-EM, molecular dynamics of L-TGF-β1/GARP, and image processing of αvβ8/L-TGF-β1/GARP bound with Fab MHG8, related to Figure 3. (A) The B-factor from cryo-EM analysis (red) and the average RMSF (black) from long molecular dynamics simulations are shown for each residue of L-TGF-β1/GARP where flexible residues correlate with high B factors. (B) The local sharpen maps (colored) superimposed in related low passed 8 Å maps (Grey in transparent) of class 3–8 from Figure 2F. (C) B factor analysis of models for (B) (D) The weighted averaged B factor of the models in (B) (Left) compared to apo L-TGF-β1/GARP (Right). (E) Image processing flow-chart from representative electron micrograph to the final reconstruction and FSC for final resolution estimation of αvβ8/L-TGF-β1/GARP bound with Fab MHG8. (F) SDS-PAGE of αvβ8fl-nd. (G) Image processing flow-chart from representative electron micrograph to the final reconstruction and FSC for final resolution estimation of αvβ8fl-nd/L-TGF-β1/GARP. (H) The binding affinity assay of L-TGF-β1/GARP with αvβ8tr, αvβ8tr-clasped and αvβ8fl-nd showing no difference. [file NIHMS2024290-supplement-4.tif]

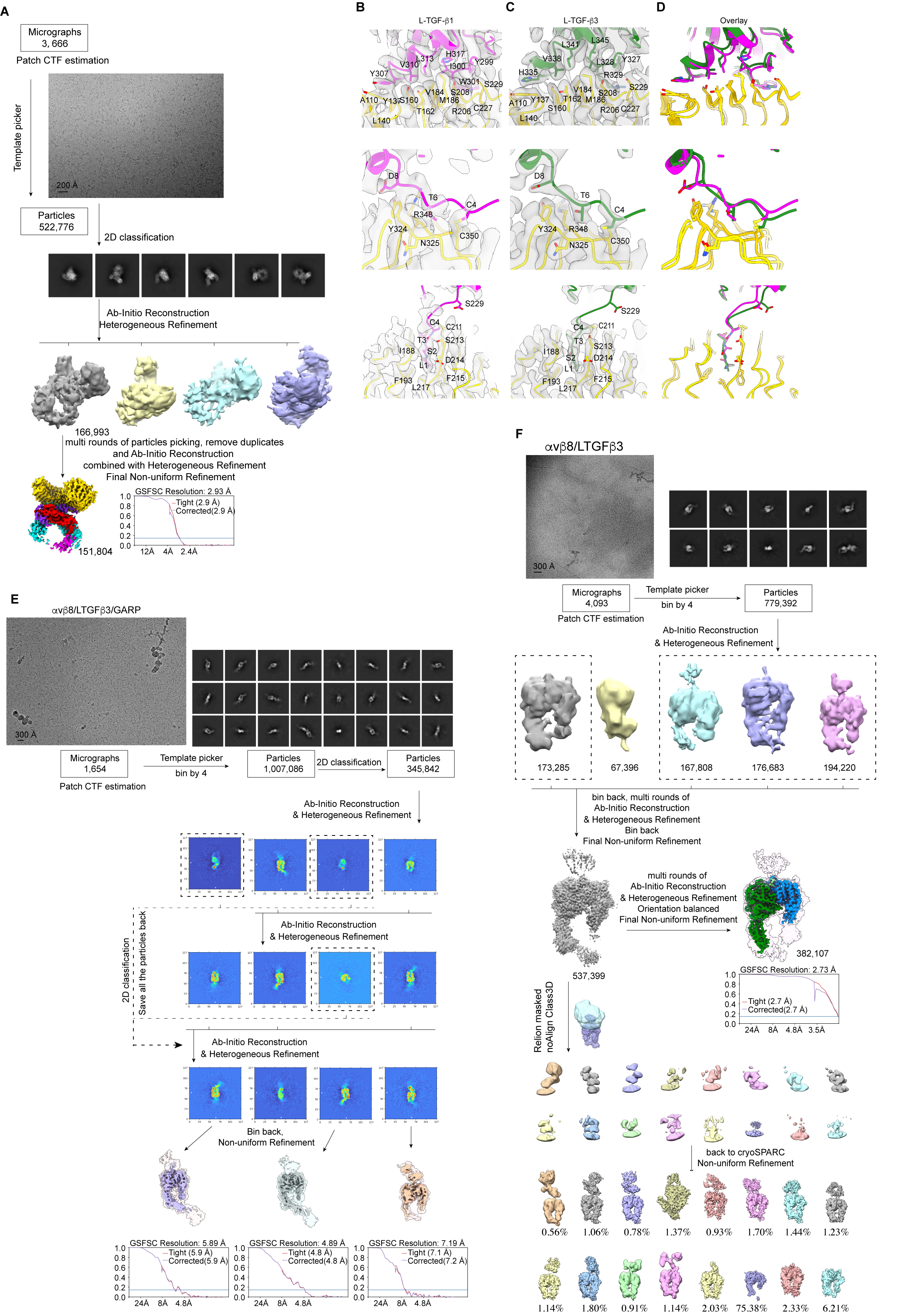

Supplement: 5 — Figure S5. Single particle cryo-EM and image processing of L-TGF-β3/GARP, αvβ8/L-TGF-β3/GARP complex and comparison of binding interface between GARP and L-TGF-β, related to Figure 4. (A) Image processing flow-chart from representative electron micrograph to the final reconstruction and FSC for final resolution estimation for L-TGF-β3/GARP. (B) Three regions of cryo-EM density map and docked atomic model selected from the interface between L-TGF-β1 (magenta) and GARP (yellow). (C) Three regions of cryo-EM density map and docked atomic model selected from the interface between L-TGF-β3 (green) and GARP (yellow). (D) Overlay of the atomic models of L-TGF-β1/GARP (magenta/yellow) and L-TGF-β3/GARP (green/yellow) from the same selected regions. (E) Flow-chart of processing single particle cryo-EM dataset of αvβ8/L-TGF-β3/GARP complex. (F) Flow-chart of processing single particle cryo-EM dataset of αvβ8/L-TGF-β3 complex. [file NIHMS2024290-supplement-5.tif]

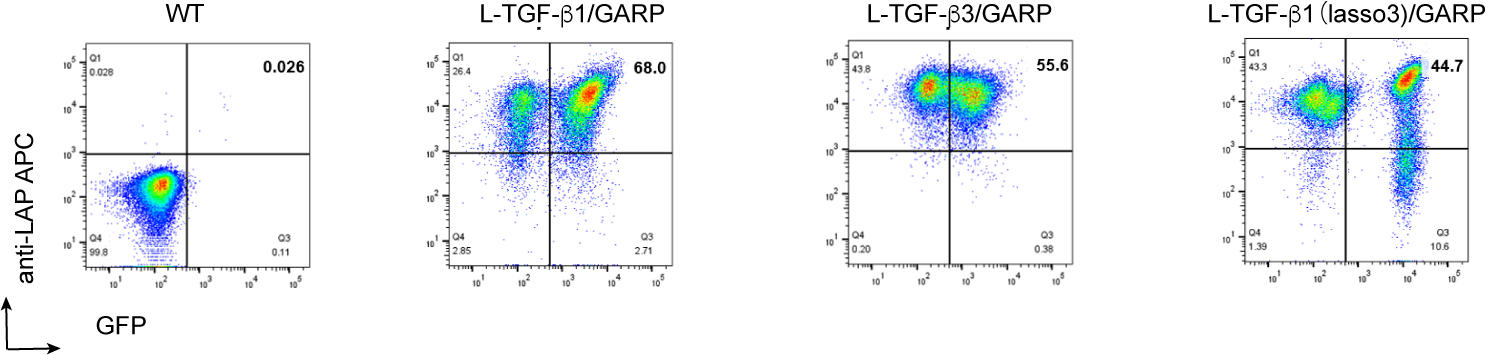

Supplement: 6 — Figure S6. Representative flow cytometry scatter plots demonstrating the percentage of cell surface L-TGF- β1, L-TGF-β3 and L-TGF- β 1(lasso3) respectively, related to Figure 5. [file NIHMS2024290-supplement-6.tif]

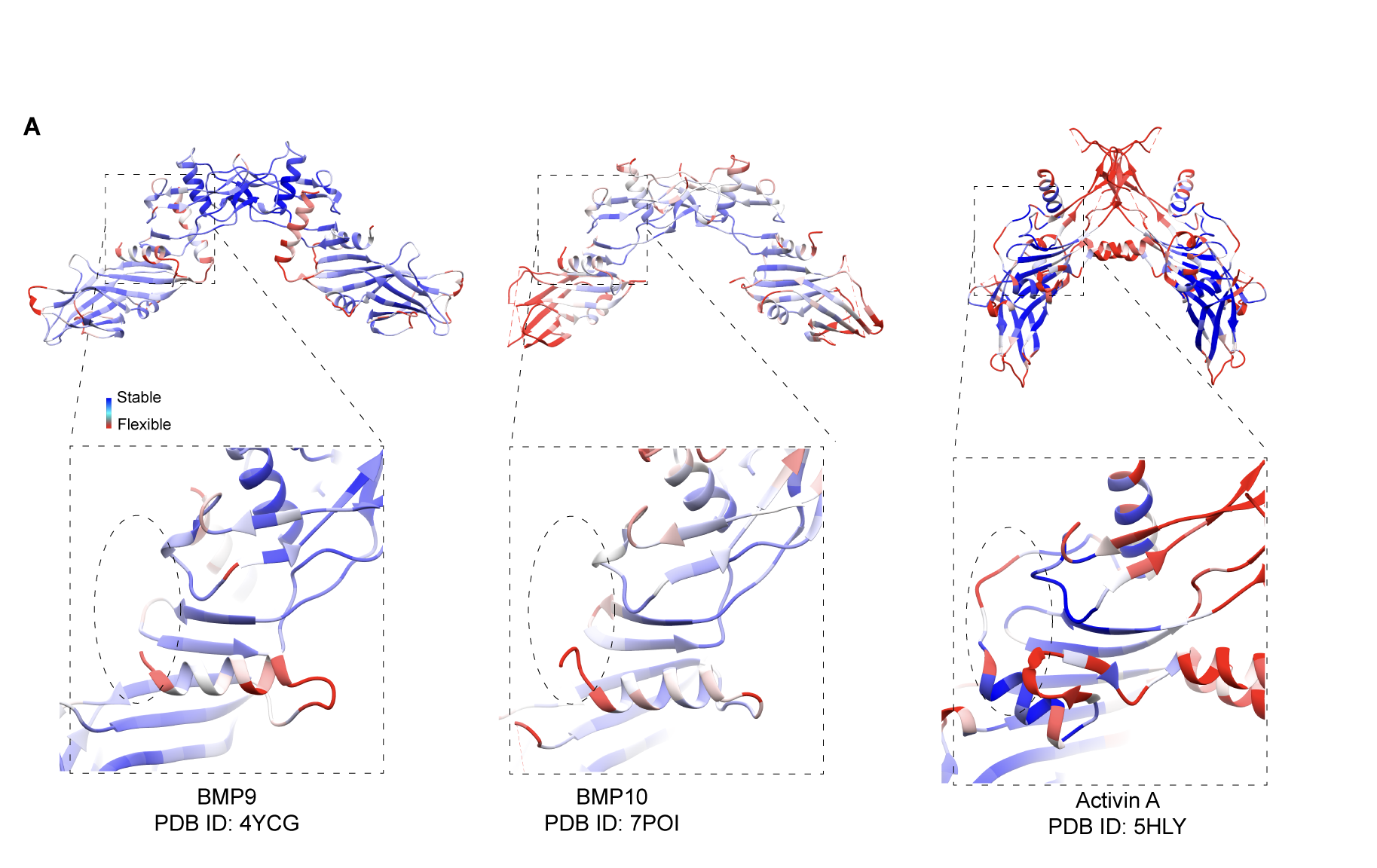

Supplement: 7 — Figure S7. B-factor for each pro-TGF-β superfamily crystal structure, related to Discussion and Figure 5. Ribbon diagram of BMP9 (PDB: 4YCG), BMP10 (PDB: 7POI), and Activin A (PDB: 5HLY). Residues are colored by B-factors. Residues with lower B-factor (blue) are relatively stable, while those with higher B-factor (red) are more flexible. An enlarged view within dashed box for each show that the area around TGF-βR binding site, in which each lasso loop (dashed oval) is flexible. [file NIHMS2024290-supplement-7.tif]

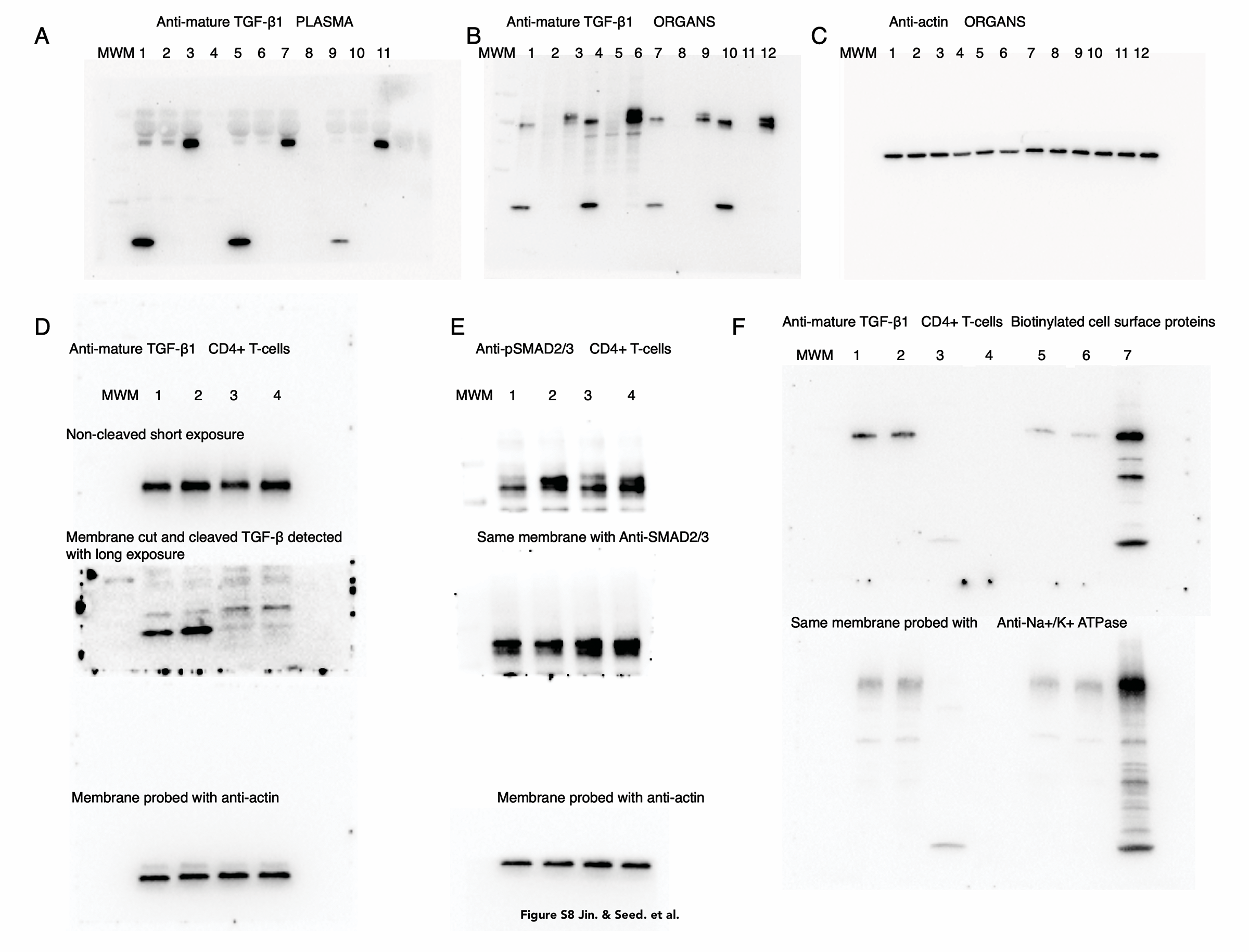

Supplement: 8 — Figure S8. Raw immunoblots, related to Figure 1. (A) Raw immunoblot of plasma data depicted in Figure 1M. Plasma was subject to 3 sequential rounds of IgG pre-clearance using Sepharose G beads. Lanes 9 (WT/WT), 10 (KO/KO) and 11(KI/KI) are depicted in Figure 1M. Samples in Lanes 1,2 and 3, and 5,6 and 7 represent the samples after 1 or 2 rounds of pre-clearing respectively. (B) Raw immunoblot of TGF-β1 expression in murine organ homogenates (Figure 1M). Lanes 1,2 and 3 show TGF-β1 staining from WT/WT, KO/KO and KI/KI mice respectively in kidney, Lanes 4, 5 and 6 in liver, Lanes 7, 8 and 9 in lung, and 10, 11 and 12 in spleen. (C) Raw immunoblot representing the β-actin expression depicted in figure 1M as a loading control. (D) Raw immunoblot data for Figure 1N demonstrating the expression of uncleaved TGF-β1 (upper blot) after 0.9 seconds exposure, and cleaved TGF-β1 (lower blot) after 14.9 seconds exposure. Included below the TGF-β1 blots is a raw immunoblot stained with anti-β-actin to ensure equal protein loading. (E) raw immunoblot of anti-pSMAD2/3 (upper blot) and anti-SMAD2/3 (lower blot) presented in Figure 1P. Included below the SMAD2/3 blot is an immunoblot using anti-β-actin to ensure equal protein loading. (F) Raw immunoblots for data depicted in Figure 1O. The upper blot displays anti-TGF-β1 staining. Lane 1 (non-biotinylated lysate), Lane 2 (non-biotinylated lysate after Sepharose pre-clearance), Lane 3 (eluate from pre-cleared lysate incubated with streptavidin beads), Lane 4 (blank), Lane 5 (biotinylated lysate) Lane 6 (biotinylated lysate after Sepharose pre-clearance), Lane 7 (eluate from pre-cleared biotinylated lysate incubated with streptavidin beads). The lower image depicts the raw immunoblot data depicting anti-Na+/K+ ATPase staining to demonstrate cell surface protein enrichment. Figure 1O depicts Lane 5, lane 7 and Lane 3 respectively. [file NIHMS2024290-supplement-8.tif]
