# Supplementary material for: Dynamic allostery drives autocrine and paracrine TGF-β signaling
Source: Cell. Author manuscript; Available in PMC 2024 Nov 3. (PMC11531391; doi:10.1016/j.cell.2024.08.036)
Supplement: 11 [file NIHMS2024290-supplement-11.pdf]

Table S1. Histology scores of mice, related to Figure 1K and 1L. The table represents histologic analysis and disease scoring of hematoxylin and eosin stained tissue sections of heart, lung, and liver in WT/WT, KI/KI and KO/KO.

Mouse	Genotype	Days	Weight loss?	Skin	Lung	Heart	Liver	Spleen	Kidney	Small bowel	Colon	Score (Total)
1	KO/KO	23	Yes	0	3	3	3	ND[1]	0	0	0	9
2	KO/KO	23	Yes	0	3	3	2	ND	0	0	0	8
3	KO/KO	23	Yes	1	2	3	2	ND	0	0	0	7
4	KO/KO	18	Yes	0	1	0	2	0	0	0	3	3
5	KO/KO	18	Yes	0	2	2	2	0	0	0	3	6
6	KO/KO	18	Yes	0	1	1	1	0	0	0	1	3
7	WT/WT	18	No	0	0	0	0	0	0	0	0	0
8	WT/WT	18	No	0	0	0	0	0	0	0	3	0
9	WT/WT	18	No	0	0	0	1	0	0	ND	0	1
10	WT/WT	23	No	0	0	0	0	0	0	0	0	0
11	WT/WT	21	No	0	0	0	1	0	0	0	0	1
12	WT/WT	21	No	0	0	0	0	0	0	0	0	0
13	WT/WT	23	No	0	0	0	0	ND	ND	0	0	0
14	KI/WT	19	No	0	0	0	0	0	0	0	0	0
15	KI/WT	21	No	0	0	0	0	0	0	ND	ND	0
16	KI/WT	21	No	0	0	0	0	0	0	0	0	0
17	KI/WT	63	No	0	0	0	0	0	0	0	0	0
18	KI/KI	31	Yes	0	0	0	0	0	0	0	0	0
19	KI/KI	21	No	0	0	0	0	0	0	0	0	0
20	KI/KI	19	No	0	0	0	1	0	0	0	0	1
21	KI/KI	19	No	0	0	0	0	0	0	0	0	0
22	KI/KI	22	Yes	0	0	1	1	ND	0	0	0	2
23	KI/KI	22	No	0	1	1	1	ND	0	0	0	3
24	KI/KI	122	No	0	1	0	0	ND	0	0	0	1

[1] Not Done
